# Electrochemical CO_2_ Reduction to Multicarbon Fuels and Chemicals: Progress and Prospects of Tandem Electrolyzer Strategies

**DOI:** 10.1021/acs.energyfuels.5c04464

**Published:** 2025-10-22

**Authors:** Jin Feng, Ahmed Badreldin, Ying Li

**Affiliations:** † J. Mike Walker ’66 Department of Mechanical Engineering, 14736 Texas A&M University, College Station, Texas 77843, United States; ‡ Department of Chemical Engineering, University of Mississippi, Oxford, Mississippi 38677, United States; § Artie McFerrin Department of Chemical Engineering, Texas A&M University, College Station, Texas 77843, United States

## Abstract

Tandem electrolyzer strategies represent an innovative and stepwise evolution toward the commercialization of electrochemical CO_2_ reduction. This review provides a comprehensive introduction to the fundamentals of tandem electrolyzers in catalysis, highlighting their significant advantages over traditional single-cell approaches, particularly in terms of electrical energy efficiency and cost-effectiveness. A detailed discussion is presented on experimental designs, including electrolyzer configurations and optimal reaction environments. Contemporary tandem research is categorized based on targeted products, clearly illustrating how tandem designs enhance performance metrics. By deconvoluting the complex direct conversion of CO_2_ into two or more simplified, independently optimized steps, tandem strategies effectively mitigate or eliminate the persistent challenges of carbon loss and carbonate formation inherent in alkaline CO_2_ reduction. This approach enhances the efficiency of individual reaction steps, improves overall process performance, extends the product range, and enables innovative versatile sequential reaction designs powered by renewable energy. Such strategies offer promising routes to overcoming longstanding barriers in CO_2_ electrolysis, with implications for both industrial deployment and broader decarbonization efforts. Looking ahead, further advances will depend on addressing single-cell issues in catalyst selectivity and stability and on rigorous, tandem-level scale-up demonstrations, accompanied by comprehensive techno-economic analyses.

## Graphical abstract

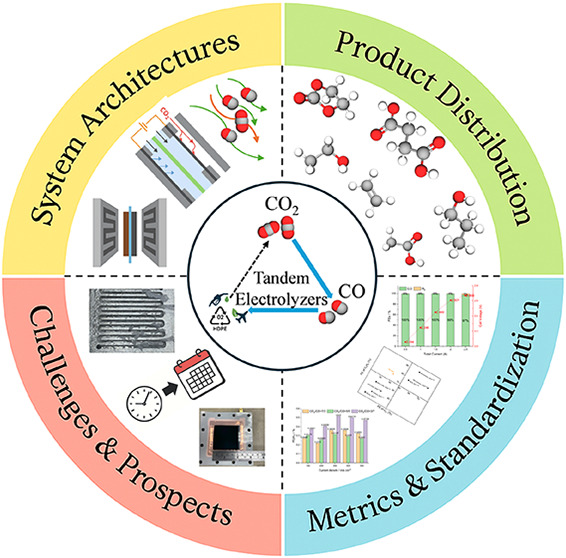

## 1 Introduction

Carbon materials are extremely important as the key to energy storage and conversion in ecological balance; their oxidation and reduction constitute the core of our energy and chemical industries.^1,2^ But compared with the natural carbon cycle, our human industry lacks a part that can reduce CO_2_ like photosynthesis in plants. Since the Industrial Revolution, the sharp increase in the use of fossil fuels has led to an uncontrolled increase in the greenhouse gas (GHG) CO_2_ in the atmosphere. The global atmospheric CO_2_ concentration has increased from a preindustrial level of approximately 278 ppm in 1750 to around 422 ppm in 2024, representing an increase of about 52%.^3^ This has led to a series of environmental problems such as global warming and ocean acidification,^4^ more extreme weather events,^5^ sea level rise,^6^ and challenges to crop yields.^7^ Although the signatories of the Paris Climate Agreement have pledged to achieve the goal of “achieving a balance between anthropogenic GHG emissions by sources and removals by sinks in the second half of this century”, the issue of how to fulfill the commitment needs to be urgently addressed and implemented.

Electrochemical engineers now view CO_2_ as an abundant carbon feedstock, especially with the growing availability of renewable energy for producing fuels and chemicals.^8–12^ In 2024 alone, global renewable power capacity grew by a record 585 Gigawatts (GW), accounting for over 90% of all new power capacity additions, with an annual growth rate of 15.1%, and is projected to continue expanding rapidly through 2030.^13^ With the global shift toward renewable energy, in part due to their near cost-parity with conventional grid electricity analogues, the integration of CO_2_ utilization technologies with clean electricity sources, such as solar, wind, and hydropower, has opened promising pathways for decarbonizing industrial processes.^14^ In particular, atmospheric CO_2_ can be captured^15^ through a plethora of regimes including, but not limited to, direct air capture, point-source capture, and seawater capture, and subsequently converted into value-added products like carbon monoxide (CO),^16^ methane (CH_4_),^17,18^ formic acid (HCOOH),^19^ methanol (CH_3_OH),^20–22^ ethylene (C_2_H_4_),^23^ acetic acid (CH_3_COOH),^24^ ethanol (CH_3_CH_2_OH),^25^ acetaldehyde (CH_3_CHO),^26^ and propanol (C_3_H_8_O),^27^ allyl alcohol,^28^ aromatics,^29^ and polymers,^30^ solid carbon products,^31^ and long-chain liquid hydrocarbon^32,33^ via electrochemical or combined with thermochemical processes. These energy-intensive conversions become environmentally benign and foreseeably attain a pathway toward becoming economically viable when powered by renewable energy, creating a synergy between CO_2_ utilization and clean power that reduces emissions and supports circular carbon and energy storage strategies.

Rapid progress has been seen in the industrial deployment of electrochemical CO_2_ reduction reaction (eCO_2_RR). Notably, in May 2024, CarbonFree signed a definitive agreement with U.S. Steel to deploy its SkyCycle technology at U.S. Steel’s Gary Works in Indiana, one of the largest integrated steel mills in North America.^34^ This partnership represents one of the first large-scale applications of CO_2_ capture and utilization at an American steel plant, with the goal of capturing and mineralizing up to 50,000 metric tons of CO_2_ annually. In 2024, the California startup Twelve reached a significant milestone in advancing CO_2_-to-chemicals technology by completing the installation, commissioning, and validation of a low-rate initial production pilot line for manufacturing membrane electrode assemblies (MEAs).^35^ This plant is designed to produce sustainable aviation fuel, delivering more than a 90% reduction in well-to-wing greenhouse gas emissions compared with conventional Jet A fuel. When deployed at full scale, Twelve’s carbon conversion platform has the potential to process between 2 and 3 billion metric tons of CO_2_ annually, representing a transformative opportunity within the petrochemicals sector, a market valued at over $600 billion. In May 2025, OCOchem, based in Richland, WA, successfully brought online and commissioned the world’s first pilot facility capable of producing hydrogen formate and potassium formate at an industrial scale, which is designed for an annual output of 60 tons and operates using only CO_2_ and water as feedstocks through a CO_2_ electrolysis process that employs commercial-grade gas diffusion electrodes (GDEs) with individual surface areas of 1.5 m^2^.^36^ Independent analysis by EcoEngineers indicates that for every ton of formate generated by this system, a total of 7.2 tons of CO_2_ emissions are eliminated, both through direct utilization of captured CO_2_ and by replacing fossil-fuel-derived feedstocks and energy sources traditionally used in chemical manufacturing.^36^ These efforts mark significant industrial progress across the entire CO_2_ utilization chain, from capture to electrolyzer development and CO_2_ electrolysis product formation, representing major steps toward the commercialization of eCO_2_RR technologies.

Besides U.S.-based initiatives, substantial progress has also been made internationally. In Europe, the UK company Carbon Clean in May 2025 successfully completed factory tests of its CycloneCC C1 modular carbon-capture system at commercial scale, demonstrating the capture of up to 285 tonnes CO_2_ per day.^37^ In Belgium, D-CRBN launched a CO_2_-recycling pilot line in October 2023 that converts CO_2_ into CO using plasma technology, with an annual capacity of approximately 1000 tonnes.^38^ In Germany, Greenlyte Carbon Technologies began in September 2025 constructing a pilot plant at Düsseldorf Airport, partially powered by on-site photovoltaics, to produce CO_2_-based sustainable aviation fuel (≈150 tonnes per year, equivalent to roughly 60 short-haul flights under current blending limits).^39^ In Asia, while Korea and China have published extensive fundamental research on eCO_2_RR, few pilot-scale or industrial-level deployments have been reported to date. Notably, in China, Carbon Energy Co., Ltd. operates a commercial CO_2_ electrolysis syngas demonstration plant, reportedly producing tens of tonnes per year, underscoring the nation’s commitment to industrial-scale CO_2_ utilization infrastructure.^40^ In Japan, Toshiba Energy Systems & Solutions, in collaboration with Cosmo Energy and ENEOS, is advancing CO_2_-to-CO electrolysis toward refinery integration.^41,42^ Although fully commercial eCO_2_RR plants have not yet been verified in the region, these programs collectively signal increasing industrial readiness.

Furthermore, the development of eCO_2_RR technologies aligns with expanding commercial opportunities driven by growing consumer awareness and demand for sustainable products. A new survey reveals 74% of Americans would choose a CO_2_-based product over conventional oil-derived products if they are equal in quality.^43,44^ This trend is reinforced by the rise of carbon monetization.^45^ In 2023, global carbon pricing initiatives covered approximately 24% of global GHG emissions and generated over 104 billion USD in revenue through compliance and voluntary markets.^46^ In parallel, environmental, social, and governance (ESG) investing has surged, with global ESG assets expected to exceed 40 trillion USD by 2030, equivalent to more than 25% of total projected $140 trillion assets under management, signaling strong financial incentives for corporations adopting low-carbon technologies such as CO_2_ electroreduction.^47^

The future of turning CO_2_ into valuable products by electrochemical methods is becoming more attractive and practical, particularly in light of recent research that has deepened the fundamental understanding of catalyst–intermediate interactions, addressed key bottlenecks such as mass transport limitations, and demonstrated promising approaches for scale-up and techno-economic viability.^48–56^ The conversion of CO_2_ into value-added products can be achieved through several distinct pathways, including electrochemical, biochemical, photochemical, thermochemical, plasma-assisted, and emerging hybrid approaches that integrate multiple mechanisms. Among these, electrochemical pathway offers a scalable and flexible platform for industrial CO_2_ utilization. Compared to other chemical CO_2_ reduction pathways, the electrochemical approach offers several notable advantages: (1) it operates under ambient temperature and pressure conditions; (2) the reduction products can be selectively controlled by adjusting parameters such as applied potentials, electrolyte composition, and employed electrocatalysts; (3) it enables the transformation and storage of renewable electricity in the form of chemical products; and (4) carbon-containing fuels, traditionally obtained from fossil resources, can instead be synthesized via eCO_2_RR, thereby reducing global dependence on fossil fuels and enhancing energy security.

However, unavoidable predicaments exist in eCO_2_RR which push back on the technology’s application potential. For instance, salt precipitation problems, related to the unavoidable formation of (bi)­carbonate in conventional alkaline conditions used in eCO_2_RR, result in increased cell resistance. Further, this inadvertently blocks the catalyst surface and gas diffusion channel, hampering mass transport, and accelerates electrolyte flooding, which ultimately shuts down the cell.^57^ The salt precipitation process and mechanism in MEA electrolyzer are illustrated in Figure 1a–d. First, at the catalyst/anion exchange membrane (AEM) interface, eCO_2_RR produces OH^–^ in situ which reacts with excess CO_2_ molecules, resulting in the formation of carbonate ions (Figure 1b). Then, K^+^ crosses the AEM and combines with carbonate ions, forming K_2_CO_3_/KHCO_3_, which move away from the catalyst/AEM interface and precipitate out on the GDE backside (Figure 1c) due to concentration saturation limits. Finally, after long operation, the continuous accumulation of precipitated KHCO_3_ on the back side of GDE blocks the gas flow channels (Figure 1d).

**Figure 1:**
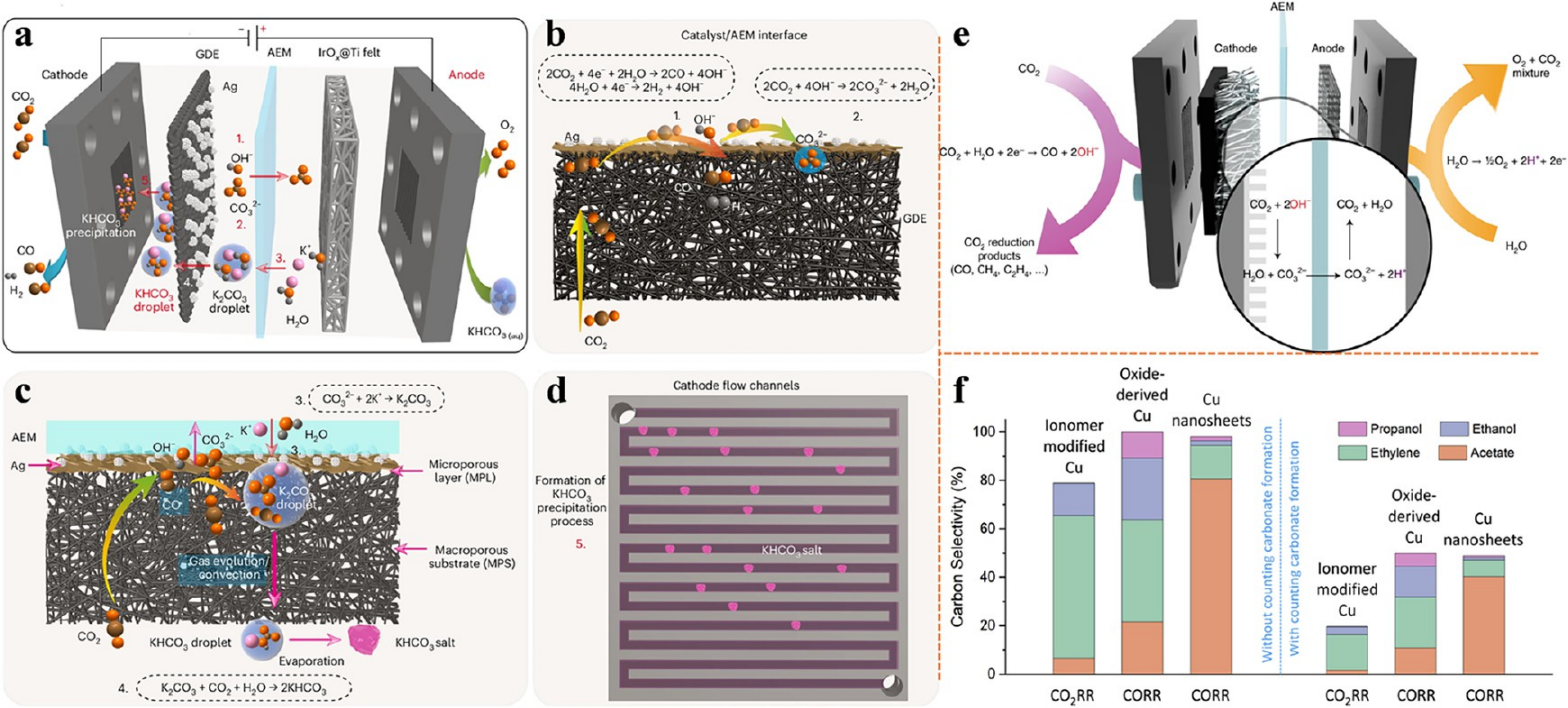
Schematic of possible carbonate formation mechanism, salt precipitation process, and CO_2_ crossover phenomenon in MEA. (a) Salt precipitation process. Reproduced from ref 16. Copyright 2025 Springer Nature. (b) Potential reactions occurring at the catalyst/AEM interface. Reproduced from ref 16. Copyright 2025 Springer Nature. (c) Formation and migration of K_2_CO_3_/KHCO_3_ droplet. Reproduced from ref 16. Copyright 2025 Springer Nature. (d) Continuous accumulation of KHCO_3_ precipitations on the GDE backside. Reproduced from ref 16. Copyright 2025 Springer Nature. (e) CO_2_ crossover phenomenon. Reproduced from ref 61. Copyright 2022 Springer Nature. (f) Carbon selectivity (Carbon loss) results with/without considering carbonate formation. Reproduced from ref 62. Copyright 2021 Elsevier.

The unavoidable formation of (bi)­carbonate not only causes salt precipitation problems but subsequently results in a symbiotic loss of carbon. Substantial CO_2_ loss into the electrolyte translates to a non-negligible electrolyte regeneration fee,^58–60^ low carbon utilization rate, and relatively low CO_2_ single-pass conversion efficiency (SPCE).^59,60^ In congruence to the salt precipitation process, after carbonate ions are generated at the cathode, they migrate across the typical anion exchange membrane into the anode compartment, rather than solely combining with K^+^ and diffusing to the backside of the GDE. Within the anode, carbonate ions either remain in ionic form or react with in situ generated protons to form CO_2_, which is subsequently released from the anode side. A schematic of the CO_2_ crossover phenomenon in anionic MEA cells is depicted in Figure 1e. These CO_2_ molecules do not participate in the eCO_2_RR and are effectively lost from the cathode CO_2_ stream. When this loss is considered in the calculation of carbon selectivity, the resulting values are significantly reduced (Figure 1f). Additionally, anodically present CO_2_ results in increased downstream anodic separation costs from pure O_2_ produced from water oxidation.

Moreover, direct eCO_2_RR results in low selectivity to desired multicarbon products, due to complex reaction pathway and kinetics involving CO_2_ molecules,^63,64^ and competing cathodic hydrogen evolution reaction (HER). The intricate reaction pathways, encompassing numerous bond-breaking and bond-forming steps, multiple electron transfer events, and overlapping thermodynamic potential windows, create substantial obstacles to achieving high selectivity for highly reduced products. Although simple compounds such as CO and HCOOH can be efficiently produced using one kind of catalyst with one kind of active site, directly converting CO_2_ into more complex molecules from the conventional eCO_2_RR process, including C_2_H_4_, C_2_H_5_OH, CH_3_COOH, and C_3_H_7_OH, remains a significant challenge.

Thus, tandem eCO_2_RR has emerged as a promising approach to overcome or alleviate the aforementioned challenges. Traditionally, tandem catalysis refers to two primary configurations. (1) Tandem catalysts: at the microscopic or atomic scale, distinct catalyst sites are dispersed such that different active sites are adjacent and can interact through mechanisms such as CO spillover (Figure 2a–c).^65–70^ (2) Tandem electrodes: at the macroscopic or electrode scale, distinct catalysts sites are arranged in separate, well-defined regions, such as layers or segments, within a single reactor, enabling spatially separated tandem reactions (Figure 2d,e).^71,72^ In addition, a related and increasingly explored concept is that of tandem reactors, where two or more independent reactors are sequentially connected—each under independently optimized conditions.^73^ The term tandem reactors broadly refers to the emerging concept of sequentially linked independent reactors, encompassing any arrangement in which two or more reactors, such as electrolyzers, thermochemical reactors, biochemical reactors, or combinations thereof are connected in series so that the product stream from the first reactor directly serves as the feed for the next. In contrast, tandem electrolyzer strategies describe systems in which two or more electrolyzers are coupled in sequence, representing a specialized subset of tandem reactor strategies where the combination of reactors is limited to electrolyzers.

**Figure 2:**
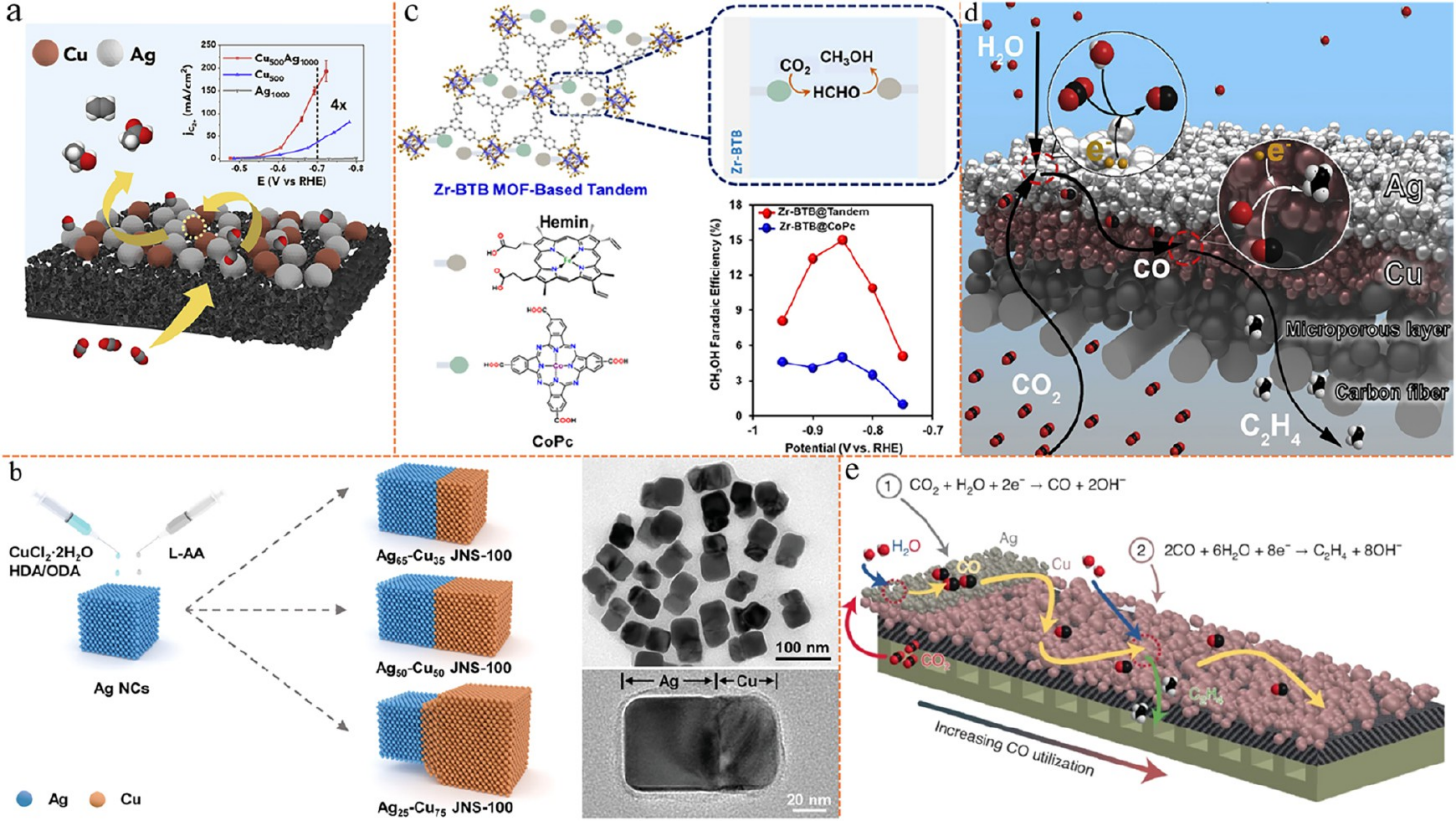
Schematic illustration of traditional tandem catalysis, including the microscopic atomic scale and macroscopic electrode scale. (a) Microscopic scale Cu–Ag two different catalysts tandem platform. Reproduced from ref 68. Copyright 2020 Elsevier. (b) Synthesis of the microscopic scale Ag–Cu JNS-100 tandem catalyst. And TEM images of Ag_65_–Cu_35_ JNS-100. Reproduced from ref 69. Copyright 2022 John Wiley and Sons. (c) Microscopic scale multifunctional CoPc and hemin-modified Zr-BTB MOF tandem catalyst. And representation of the conversion principles and performance. Reproduced from ref 70. Copyright 2025 American Chemical Society. (d) Macroscopic scale spatially decoupled Cu–Ag bilayer tandem electrode structure. Reproduced from ref 72. Copyright 2020 Elsevier. (e) Macroscopic scale of CO_2_–CO–C_2_H_4_ tandem conversion of Ag and Cu catalysts over stacked segmented GDE. Reproduced from ref 74. Copyright 2022 Springer Nature.

Among all eCO_2_RR methods, tandem strategies offer several potential benefits.^73^ First, they break down the original complex single-step process into multiple, simpler reactions, enabling the optimization of each step, including the dynamic tuning of operating conditions such as pH, temperature, and potential between stages. This flexibility maximizes selectivity and efficiency for each transformation, often yielding a synergistic effect where overall system performance surpasses the single-step process. Second, tandem approaches expand the design space for reaction chemistries, making it possible to couple unique thermodynamic and kinetic processes to synthesize more complex or higher-value products that may be impractical or impossible to access via direct single-step reduction. Third, intermediate separation and purification can often be simplified, particularly for gaseous intermediates like CO, reducing safety risks, operational costs, and environmental impacts related to hazardous material storage and transport. Fourth, in tandem catalyst designs, these systems can harness short-lived or highly reactive surface and gaseous intermediates that would otherwise dissipate in single-catalyst direct eCO_2_RR, thus opening new mechanistic pathways. Lastly, tandem reactor strategies, whether electrochemical-electrochemical (EC-EC), electrochemical-thermochemical (EC-TC), or other hybrids, provide modularity and scalability, facilitating future integration with both centralized or decentralized power sources or further coupling with thermochemical or biochemical processes to expand the portfolio of sustainable and economically viable products.

Despite the promising advantages of tandem strategies in eCO_2_RR, this field is still in its early stages, particularly for tandem electrolyzer strategies. To address key limitations of direct eCO_2_RR, such as carbon loss and complex reaction networks, tandem electrolyzer strategies are drawing increasing attention and are now often favored over tandem catalyst or tandem electrode strategies that operate in a single cell. Also, tandem electrolyzer configurations (EC-EC) are more aligned with current research trends in eCO_2_RR, representing a direct improvement and logical progression from conventional single-step eCO_2_RR systems, whereas hybrid tandem systems introduce entirely new directions and considerations for the field. The holistic engineering benefit in decoupling multistep proton-coupled electron transfer steps in electrolyzers in series is not only applicable to eCO_2_RR, but to other electrosynthesis techniques and multielectron transfer electrochemical reactions of interest. Notwithstanding, most existing reviews focus on tandem catalysts in single-cell systems, and only a few cover the broader field of tandem strategies for eCO_2_RR. These reviews are either narrowly centered on catalyst design in tandem reactors, overly general in introducing all tandem systems from catalysts to reactors to reactions, or were published before the recent rapid advances in tandem electrolyzers for eCO_2_RR. Moreover, the latest research on tandem electrolyzers, regardless of methodology or objectives, remains inconsistent, difficult to compare, and lacks deeper-level guidance for practicing researchers.

To the best of our knowledge, this article is the first comprehensive review devoted exclusively to tandem electrolyzers for eCO_2_RR. It integrates (i) the introduction and fundamentals of tandem electrolyzer strategies, including their rational evolution and detailed evidence-supported comparison with other eCO_2_RR strategies or possible alternatives; (ii) experimental design perspectives, covering major components such as electrolyzers, catalysts, and reaction conditions; (iii) product-specific progress and contemporary breakthroughs in most recent years; (iv) performance metrics and benchmarking, with an emphasis on standardization relevant to both academia and industry; and (v) key challenges such as carbonate formation, separation, and scale-up, together with potential solutions.

This review fills a critical gap by providing a unified perspective that combines scientific and engineering viewpoints, linking fundamental theory, laboratory studies, experimental processes, system-level engineering, and practical operation considerations. It aims to deliver a timely toolkit of strategies and resources at a pivotal moment preceding the boom toward large-scale adoption and commercialization of eCO_2_RR beyond small-scale spinoff efforts.

## 2 Fundamentals of Tandem Electrolyzer Strategies for CO_2_ Reduction

The tandem electrolyzer strategies have gained attention as effective means of addressing the inherent drawbacks of single-step eCO_2_RR, particularly in solving the carbon loss/carbonate formation problem and improving selectivity, electrical energy efficiency (eEE), and product complexity. In conventional eCO_2_RR, directly converting CO_2_ into multicarbon products is hindered by major challenges such as low eEE and poor selectivity, arising from high overpotentials, complex reduction pathways, competing side reactions, and overlapping potential ranges. Regarding the intricate reduction pathways, typical eCO_2_RR products include four C_1_ compounds, CO, CH_4_, HCOOH, CH_3_OH; four representative C_2_ products C_2_H_4_, CH_3_COOH, CH_3_CH_2_OH, and CH_3_CHO; and additionally, C_3_ products such as CH_3_CH_2_CH_2_OH, not to mention a variety of minor products like CH_3_CH_2_CHO and CH_3_CH_3_. In total, dozens of possible products can be formed, with many sharing common reaction intermediates, further complicating the mechanistic landscape. The possible reaction pathways leading to C_1_–C_4_ carbon-containing products from CO_2_ electroreduction are illustrated in Figure 3a. In addition to the complex reaction pathways discussed above, the electrochemical reactions and their corresponding equilibrium potentials are very similar and fall within a narrow potential window, as shown in Figure 3b, which further exacerbates the challenge of achieving high product selectivity. Tandem designs address these issues by decoupling the overall reduction pathway into multiple sequential steps, breaking down the complex electron transfer reaction into multiple reaction steps with fewer electron transfers, enabling the independent optimization of active sites and electrolysis conditions for each stage, tailored to specific reaction intermediates and desired end products.^64^

**Figure 3:**
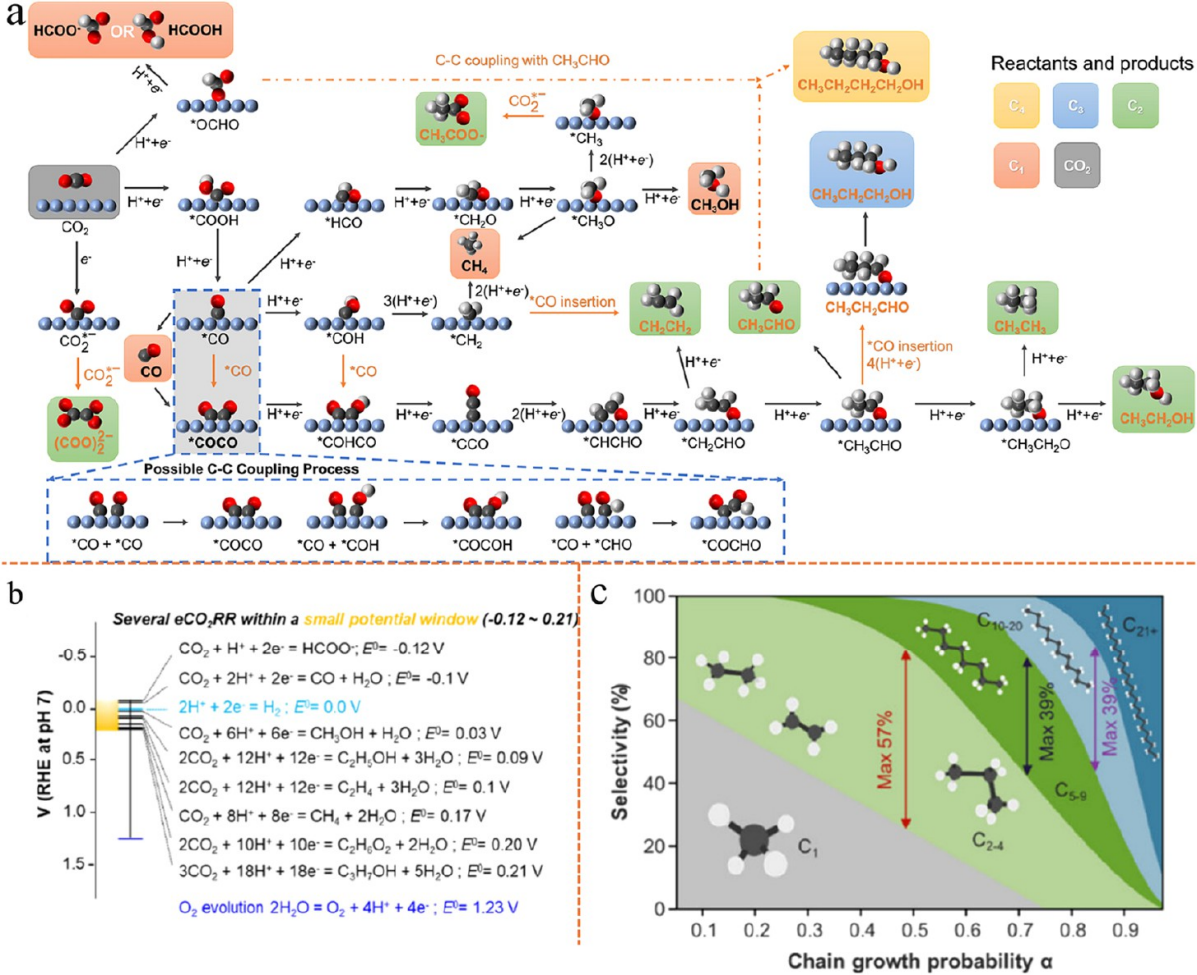
Intrinsic challenges that hinder single-cell eCO_2_RR. (a) Possible reaction route to C_1_–C_4_ carbon-containing products from CO_2_ electroreduction. Reproduced from ref 77. Copyright 2023 American Chemical Society. (b) Electrochemical reactions and their corresponding equilibrium potentials. Reproduced from ref 83. Copyright 2025 American Chemical Society. (c) Relationship between chain growth probability and product selectivity based on Anderson–Schulz–Flory (ASF) distribution. Reproduced from ref 83. Copyright 2025 American Chemical Society.

Generally, tandem electrolyzer strategies offer several significant benefits. First, it decouples the complex single-step CO_2_ reduction pathway into multiple sequential and simple steps, enabling individual optimization of each stage to achieve the overall target. Second, because both stages employ similar electrochemical cells, tandem electrolyzer systems allow for flexible integration of modular units, which greatly eases scalability and adaptation to larger-scale processes. Third, this approach leverages the strengths of established techniques for each step, for example, mature CO_2_-to-CO conversion methods^75,76^ and the growing body of electrochemical CO reduction reaction (eCORR), ensuring high performance at every stage. Fourth, compared with stand-alone eCORR, the tandem design allows for the safe stabilization and immediate utilization of hazardous intermediates such as CO, without the risks and costs of intermediate purification, storage, or transport. Fifth, the use of purely electrochemical methods means these processes operate under mild conditions, with no need for harsh temperatures or pressures of typical conventional approaches. Sixth, tandem electrolyzer systems are driven entirely by electricity and thus can take full advantage of renewable energy sources to greatly reduce the carbon footprint of chemical manufacturing. Finally, this strategy opens new opportunities to synthesize conventional, fossil-derived chemicals directly from CO_2_ and water, enabling greener, more sustainable production pathways for a wide range of valuable products.

Tandem electrolyzer strategies offer distinct advantages over tandem catalyst and tandem electrode designs in a single electrolyzer. Tandem catalyst systems often face significant challenges in identifying compatible catalysts that can operate efficiently under the same reaction conditions without being deactivated or poisoned by reaction intermediates. For example, in typical bimetallic catalysts used in tandem catalyst eCO_2_RR, the optimal potentials for C–C coupling on the Cu surface and for CO intermediate production at the cocatalyst usually do not coincide, which results in unsatisfactory selectivity toward C_2+_ products. Beyond this, for tandem electrode systems, while the concept of integrating CO-producing and CO-consuming active sites within the same electrode or structure is conceptually attractive, studies have demonstrated its intrinsic sensitivity to the spatial arrangement of catalysts, a parameter that is difficult to precisely control or maintain consistently over prolonged operation or across larger cell areas.^64^ This limitation becomes particularly prominent as systems scale up, representing a significant bottleneck for practical, large-scale applications.

Compared with tandem catalyst or tandem electrode designs in a single electrolyzer or direct eCO_2_RR (collectively referred to as single-cell eCO_2_RR), tandem electrolyzers design provides a potential benefit, the probably narrow product distribution in the second step that eCORR, is advantageous for practical use because it reduces the costs associated with product separation, resulting from the absence of other common lowly reduced CO_2_ reduction products such as CH_4_ and HCOOH, which only seen be obtained directly from CO_2_. However, this approach requires an additional gas separation step to obtain relatively pure CO from the gas mixture produced by the first-step CO_2_-to-CO electrolyzer. Additionally, single-cell eCO_2_RR continues facing persistent challenges related to carbon loss/carbonate formation. Although high-alkaline environments promote the formation of C_2+_ products, they significantly increase the energy demand for CO_2_ recovery and electrolyte regeneration. By contrast, tandem electrolyzer strategies can largely circumvent these issues: the initial CO_2_-to-CO step is performed in a mild neutral electrolyte (or even in a solid oxide electrolysis cell), effectively minimizing carbonate formation and associated carbon loss. Subsequently, the use of highly alkaline electrolytes in the second-step eCORR enables high selectivity and reaction rates for multicarbon products. Moreover, tandem design provides added flexibility in catalyst selection, electrolyzer operation, and overall system integration, making them a versatile platform for advancing eCO_2_RR technologies. Compared to single-cell eCO_2_RR, the tandem electrolyzers approach enables the synthesis of more complex compounds that are otherwise unattainable at any relevant selectivity or scale, broadening the scope of products accessible through eCO_2_RR.

Tandem electrolyzer strategies offer unique advantages compared with single-cell or hybrid tandem approaches. Currently, most eCO_2_RR focuses on C_1_ and C_2_ products, with C_1_ (CO, HCOO^–^) production already nearing commercialization and rapid progress being made on C_2_ products (C_2_H_4_, C_2_H_5_OH, and CH_3_COO^–^). However, for high-value or longer-chain hydrocarbons and oxygenates such as propanol, the growing complexity of proton-coupled electron transfer steps significantly hinders single-cell processes. Their reaction to product selectivity dramatically lowers as the length of carbon chains increases owing to the well-known scaling relations among different reaction intermediates.^77,78^ As the number of carbon atoms increases, the selectivity toward longer-chain products gradually declines, as illustrated in Figure 3c. Beyond that, very few compounds more complex than propanol and no cyclic compounds can be directly synthesized from single-cell eCO_2_RR at meaningful rates.^79,80^ In order to circumvent these limitations and access structurally more complex products, tandem strategies, featuring sequential tandem steps or the integration of different techniques such as electrochemical, thermochemical, or biochemical processes, have been proposed and explored.^29,81,82^

Most recent studies have proof-of-concept experiments highlighting that products previously unattainable via single-cell eCO_2_RR, such as high-purity ethylene,^84^ ethylene carbonate^85,86^ (EC, C_3_H_4_O_3_), and succinic acid^87^ (SA, C_4_H_6_O_4_), can now be synthesized from only CO_2_ and water feedstocks using fully electricity-driven tandem electrolyzer strategies. When coupled with renewable energy sources, this approach offers a viable route to replace traditional, high-energy-intensity, and carbon-emission fossil-fuel-based synthesis processes, opening pathways toward cleaner and more sustainable chemical production. Also studies showcased that products unattainable from the single-cell eCO_2_RR such as propionaldehyde,^88^ butane,^81^ aromatic hydrocarbons (benzene, toluene, ethylbenzene, and xylene isomers) (BTEX),^29^ poly ketone plastics,^89^ and carbon nanofibers^31^ could be yielded in good quantities following hybrid tandem reactor strategies, see Figure 4a–f. Despite their promise, hybrid tandem reactor approaches often face practical challenges, including mismatched space-time yields and differing optimal operating conditions linking the upstream stage with the downstream stage of the reaction sequence, which leads to energy-intensive separations and additional equipment costs.^90,91^ By contrast, achieving deep CO_2_ reduction to C_3+_ industrial building blocks through the cascade of two or more electrochemical devices can simplify process integration and greatly enhance industrial viability, providing a significant edge over hybrid tandem reactor strategies. It is also noteworthy that tandem electrolyzers and hybrid tandem reactor strategies are fundamentally different in both their research approaches and application targets, most notably in their product profiles, which show few overlaps. The tandem electrolyzer strategy represents an evolution of single-cell eCO_2_RR, building on the established principles of traditional electrochemical methods while introducing new innovations for the continuous, green, and sustainable production of C_2+_ chemicals. This continuity and inheritance enable the tandem electrolyzer strategy to retain all the advantages of single-cell eCO_2_RR while taking a further step toward directly addressing longstanding challenges in C_2+_ synthesis. By contrast, while hybrid tandem reactor systems have their own unique advantages and create new opportunities for products beyond the reach of conventional eCO_2_RR, they are generally less applicable to the direct production of core C_2+_ targets such as ethylene, ethanol, acetate, or propanol. Given the focus of this review and the inherent benefits of fully electricity-driven operation under mild conditions, tandem electrolyzer strategies clearly offer greater promise and relevance for advancing sustainable CO_2_ conversion.

**Figure 4:**
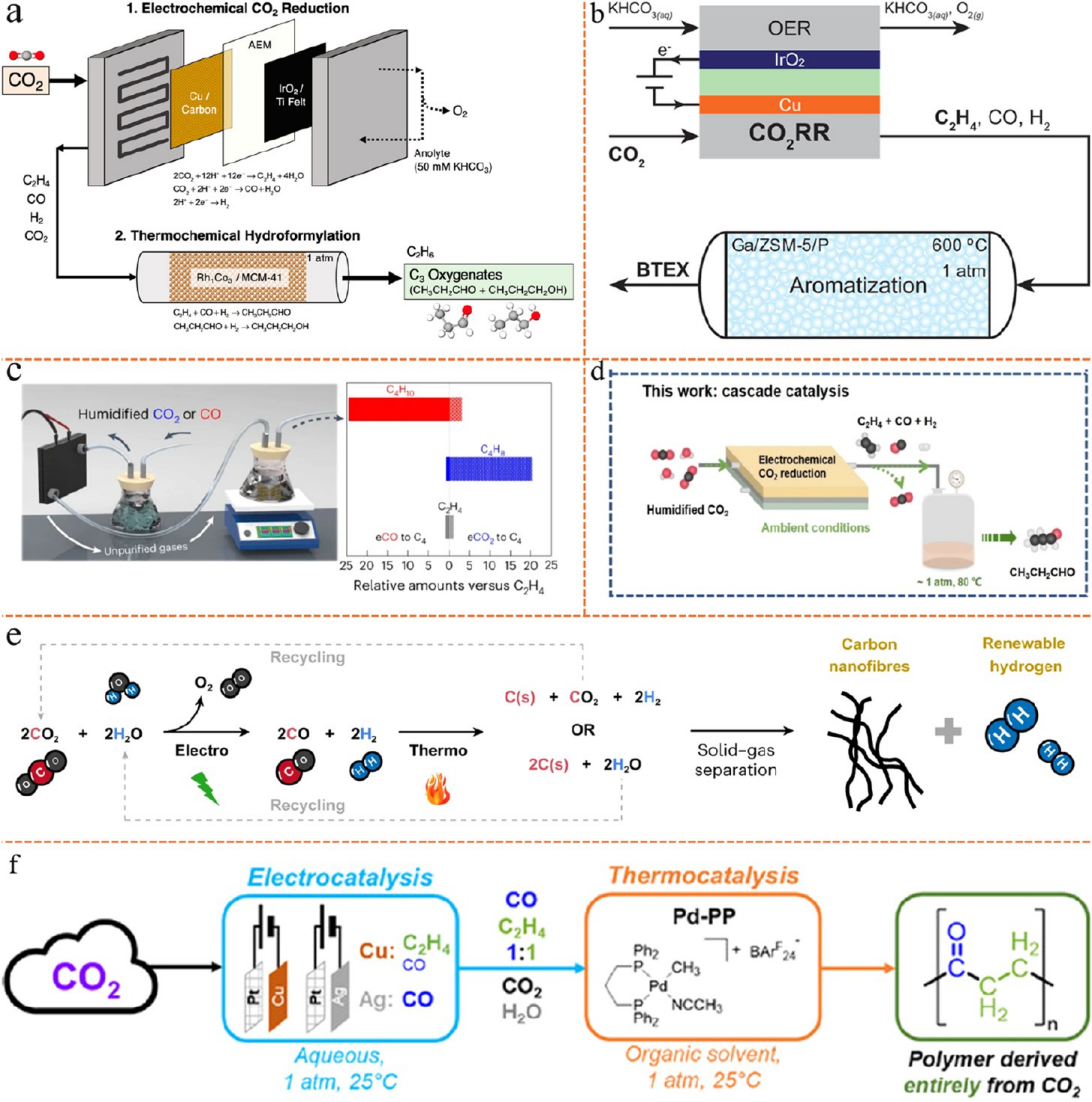
Tandem EC-TC reactors for CO_2_ reduction. (a) Tandem EC-TC reactors for CO_2_ conversion to C_3_ oxygenate products. Reproduced from ref 82. Copyright 2022 American Chemical Society. (b) Tandem EC-TC conversion of CO_2_ to aromatic hydrocarbons. Reproduced from ref 29. Copyright 2024 American Chemical Society. (c) Selective synthesis of butane from CO using cascade EC-TC at ambient conditions. Reproduced from ref 81. Copyright 2023 Springer Nature. (d) Cascade EC-TC CO_2_ reduction to propionaldehyde. Reproduced from ref 88. Copyright 2024 John Wiley and Sons. (e) Tandem EC-TC process for producing CNFs and renewable H_2_. Reproduced from ref 31. Copyright 2024 Springer Nature. (f) Tandem EC-TC system for generating entirely CO_2_-generated abiotic polymers. Reproduced from ref 30. Copyright 2025 John Wiley and Sons.

Tandem electrolyzer strategies are recognized for their high efficiency in producing C_2+_ products at relatively lower cell voltages for the second step of CO-to-C_2+_ and less overall energy consumption.^92–94^ Under alkaline conditions, eCO_2_RR has been widely demonstrated to enhance both the selectivity and production rate for C_2+_ products; however, its performance is often limited by the substantial formation of (bi)­carbonate species resulting from the reaction between CO_2_ and hydroxide ions. This side reaction not only reduces carbon efficiency but also imposes a significant energy penalty, particularly in the production of multicarbon products. Examples of typical single-cell eCO_2_RR system and tandem electrolyzer system are illustrated in Figure 5a,b. To address this issue, the two-step tandem approach has been proposed, as we can see from Figure 5b, wherein CO_2_ is first reduced to CO in a nonalkaline CO_2_ electrolyzer to minimize carbonate formation. The generated CO is then fed into a second electrolyzer operating under alkaline conditions, where it is further reduced to C_2+_ products with significantly enhanced selectivity and activity. Unlike CO_2_, CO does not readily react with hydroxide ions, enabling CO electrolysis to proceed more effectively in alkaline environments without the complication of carbonate formation.^50^ These advantages make tandem configurations more favorable than single-cell eCO_2_RR, which often suffers from low selectivity, high overpotential, and significant carbonate formation-related penalty in alkaline media.^95^

**Figure 5:**
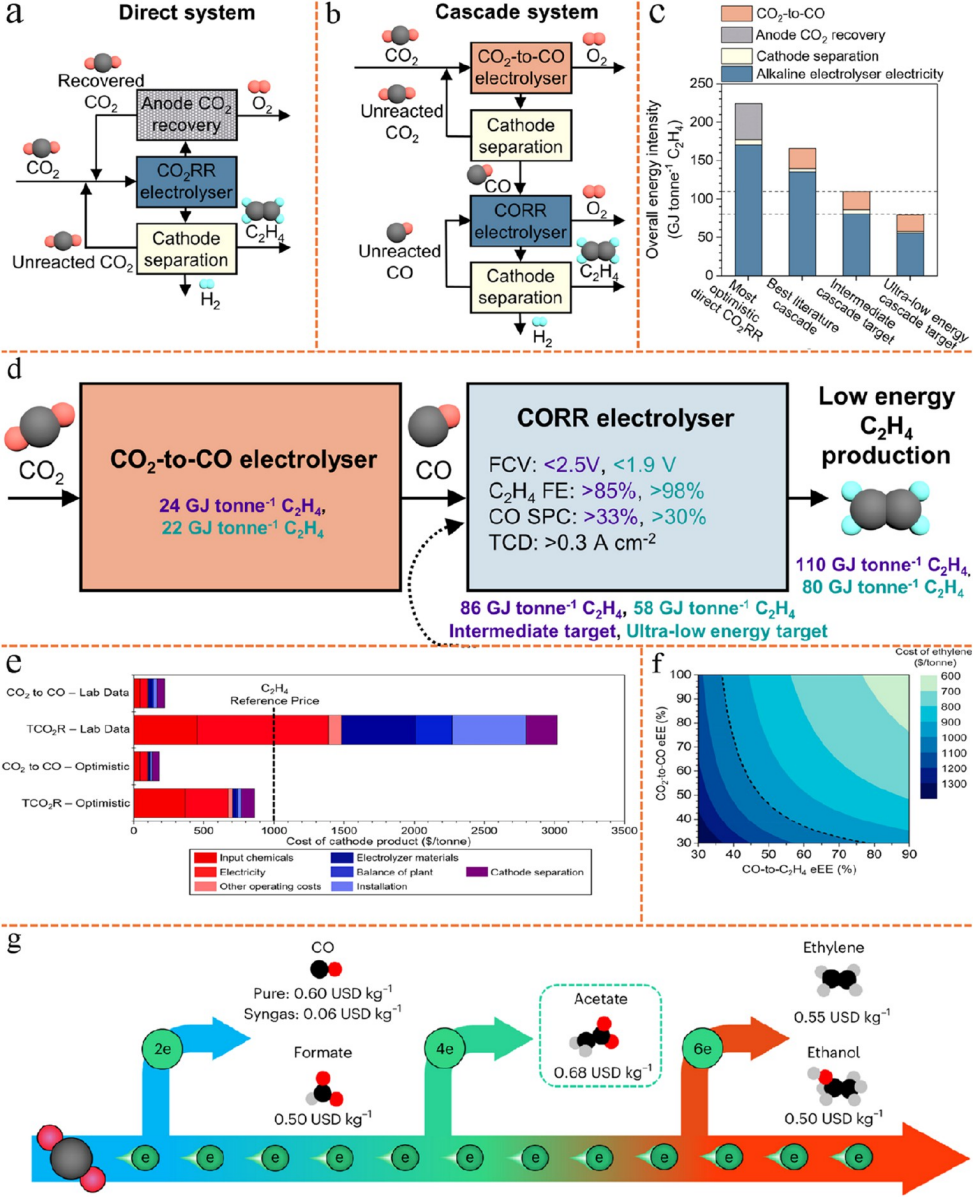
Tandem electrolyzer strategies for CO_2_ reduction energy intensity analysis and TEA. (a, b) Single-cell eCO_2_RR system and tandem electrolyzers system. Reproduced from ref 96. Copyright 2024 Elsevier. (c) Comparison of overall energy intensity values of the single-cell system with tandem electrolyzers system. Reproduced from ref 96. Copyright 2024 Elsevier. (d) Detailed requirements to achieve the intermediate/ultralow energy target for tandem approach. Reproduced from ref 96. Copyright 2024 Elsevier. (e) Cost breakdown for ethylene production in the tandem electrolyzers eCO_2_RR. Reproduced from ref 95. Copyright 2021 American Chemical Society. (f) Cost of ethylene as a function of eEE for each step of the tandem electrolyzers eCO_2_RR. Reproduced from ref 95. Copyright 2021 American Chemical Society. (g) Market price and energy requirements of common CO_2_ electroreduction products. Reproduced from ref 50. Copyright 2024 Springer Nature.

Tandem electrolyzer strategies are less energy-intensive compared with single-cell eCO_2_RR.^96^ As shown in Figure 5c, the overall energy intensity values for a direct system operating at the most optimistic eCO_2_RR SPCE,^97^ a tandem system based on one of the best literature metrics,^98,99^ and two target tandem systems are compared. Given that the production of one mole of ethylene requires two moles of CO, the energy intensity values (GJ tonne^–1^ C_2_H_4_) reported for the CO_2_-to-CO electrolyzer are calculated based on the output of two moles of CO. For the CO_2_-to-CO electrolyzer (here exemplified by a SOEC), the energy intensity values account for electrolyzer electricity consumption, heating, and cathode-side product separation. The upper dashed line indicates the intermediate overall target of 110 GJ tonne^–1^ C_2_H_4_, whereas the lower dashed line denotes the ultralow overall energy intensity goal of 80 GJ tonne^–1^ C_2_H_4_ for the tandem system. It should be noted that the energy results shown in Figure 5c do not account for system lifetime (useful life), which may lead to an underestimation of long-term energy costs. For the most optimistic single-cell eCO_2_RR system, the data are based on a MEA employed in ref 97, i.e., 3.30 V full-cell potential, 80.0% FE of C_2_H_4_, 27.9% full-cell eEE for C_2_H_4_, 200 mA/cm^2^ current density, and an assumed theoretical maximum CO_2_ SPCE of 25%. The MEA anode separation energy intensity is based on retrieving CO_2_ from the anode gas, and its value is calculated as described in ref 100. For the best literature metrics of tandem electrolyzers, the first electrolyzer data are based on a SOEC employed in ref 99, i.e., 1.06 V full-cell potential, 95% FE of CO, 815 mA/cm^2^ current density, 35% SPCE of CO_2_, and operating temperature at 800 °C; the second electrolyzer data are based on a MEA employed by ref 98, i.e., 2.23 V full-cell potential, 45.5% FE of C_2_H_4_, 21.6% full-cell eEE for C_2_H_4_, 240 mA/cm^2^ current density, and a CO_2_ SPCE of 94.6%. The results demonstrate that the energy intensity of tandem systems (<166 GJ tonne^–1^ C_2_H_4_) is generally lower than that of direct single-cell eCO_2_RR (224 GJ tonne^–1^ C_2_H_4_). The majority of this difference arises from improved CO_2_ recovery at the anode, which is related to carbonate formation/carbon loss problem in single-cell eCO_2_RR systems.

A closer analysis of the energy intensity in tandem systems reveals that further improvements are possible to achieve the intermediate or ultralow energy intensity targets for C_2_H_4_ production. The required performance metrics for both the CO_2_-to-CO and eCORR MEAs to enable low-energy C_2_H_4_ electroproduction are summarized in Figure 5d. Values in purple correspond to the overall tandem system intermediate target scenario of 110 GJ tonne^–1^ C_2_H_4_, values in teal correspond to the overall tandem system ultralow energy target scenario of 80 GJ tonne^–1^ C_2_H_4_, while values in black correspond to both scenarios. Attaining these targets necessitates simultaneous advancements in several areas: cathode kinetics, AEM ion-exchange capacity and thickness, anode kinetics, anode thickness, and operational conditions (such as KOH concentration and cell temperature). These findings underscore the significant potential of the tandem method for the low-energy, sustainable production of green C_2_H_4_.

Tandem electrolyzer strategies offer the economically profitable possibility.^95^ For example, Figure 5e presents the cost breakdown for tandem eCO_2_RR and the dotted black lines in all plots show ethylene’s reference price ($1000/tonne, $600–1200/tonne depending on region^95^), indicating that ethylene can be produced below the reference price in an optimized tandem electrolyzers system, largely due to the high eEE in the first step and the elimination of carbonate formation. Representative operating parameters adopted for the cost analysis are summarized below. A representing low-cost renewable power electricity price of 2 ¢/kWh, a 20-year system lifetime with a 5-year catalyst and membrane replacement cycle, and a pure CO_2_ feed (≈100% purity, $30/t). For the CO_2_-to-CO step, the cell operated at −1.3 V with nearly 100% FE and 78% eEE, achieving a SPCE of 50% at a current density of 475 mA/cm^2^. For the CO-to-C_2_H_4_ step, the corresponding laboratory data show 38% FE and 17% eEE at −2.32 V and 144 mA/cm^2^ with 43% SPCE. Under optimistic assumptions (1000 mA/cm^2^), the CO_2_-to-CO and CO-to-C_2_H_4_ cells were modeled with 100%/78% and 90%/53% FE/eEE, and 60%/53% SPCE, respectively.

Figure 5f applies optimistic input parameters from Figure 5e and adjusts the eEE for each stage of the tandem process to identify conditions under which ethylene can be produced at costs below the $1000 per tonne industrial benchmark. If the eEE for the first electrolyzer is set at 80%, it requires only an eEE of ∼40% for the second electrolyzer to achieve ethylene production less than $1000/tonne. The relatively high eEEs achievable in the CO_2_-to-CO step, already demonstrated with zero CO_2_ loss to carbonate, reduce the performance requirements for the subsequent eCORR step to reach profitability, making this configuration economically promising. Although concerns remain regarding the potentially higher capital investment required for tandem systems (e.g., additional electrolyzers). In Figure 5f, techno-economic analysis (TEA) indicates that, after optimization, the majority of the overall cost is attributed to chemical inputs and electricity consumption rather than capital expenditures. Therefore, without a significant reduction in carbonate formation, single-cell eCO_2_RR in any electrolyzer configuration is unlikely to achieve economic viability.

Tandem electrolyzer strategies are also considered better than only eCORR. Many recent studies concentrate exclusively on the second stage of the tandem sequence, electroreduction of pure CO, while giving little attention to the interdependence of the two steps. This narrow focus diminishes the intrinsic advantages of the tandem route, introduces additional energy, cost penalties, and safety risks for CO purification, compression, storage, and transport, and disregards the kinetic and mass-transfer synergies that can emerge when CO_2_ and CO are cofed.^101^ Many eCORR studies assume a pure CO feed originating from an upstream CO_2_-to-CO electrolyzer but evaluate performance using only a stand-alone CO electrolyzer, without implementing an actual two-cell tandem setup. In practice, the absence of real cascade integration can lead to significant discrepancies between the observed performance of the isolated second cell and its behavior within a fully coupled tandem system.^50^ Some clear reasons include the purity of CO feed in the actual feed gas of CO electrolyzer, the presence of impurities in the CO stream, such as almost unavoidable byproduct H_2_ from the upstream CO_2_-to-CO electrolyzer, which is difficult to separate, as well as residual CO_2_ and H_2_O, all of which can significantly affect the downstream CO electrolyzer.

From a commercialization and cost analysis perspective, pure CO itself is a CO_2_ reduction product, with a market price of approximately 0.60 USD/kg, the second highest among common CO_2_ electroreduction products, compared to formate at 0.50, acetate at 0.68, ethylene at 0.55, ethanol at 0.50, and syngas at 0.06 USD/kg^50,102,103^ (Figure 5g). Moreover, lots of research just use pure CO available from market. Importantly, the vast majority of industrially available pure CO is derived from fossil fuels as the primary feedstock, most commonly through processes such as steam reforming or partial oxidation of hydrocarbons. This not only raises concerns regarding GHG emissions and environmental sustainability but also undermines the decarbonization potential of downstream CO-based processes. As a result, the relatively high price and carbon-intensive production pathway of CO make eCORR less economically attractive and often nonprofitable compared to eCO_2_RR to other target products.

The proposed concept of tandem electrolyzers for eCO_2_RR involves in situ conversion of as much CO_2_ as possible in the first step, followed by feeding the resulting CO into the second-step eCORR. This approach readily overcomes the challenges associated with high-pressure CO storage and eliminates the need for handling hazardous undiluted CO. Even though eCORR shows promising performance and the approaching commercial CO_2_-to-CO eCO_2_RR technique, unfortunately, due to the risks associated with high-pressure CO storage and the high equipment maintenance costs, producing multicarbon products from CO feedstock is not a practical option for either industrial or decentralized applications. In addition to storage challenges, CO is a highly toxic and deadly gas, and its generation and use without in situ handling can pose significant safety risks and concerns. Even brief exposure to CO concentrations above 1600 ppm can be fatal within hours, and levels exceeding 6000 ppm may cause death within minutes. In contrast, CO_2_ is safe, inexpensive, and easily liquefied. Given that chemical inputs represent the largest proportion of costs in eCORR industrialization, the tandem system is undoubtedly the more advantageous option.^53^

When examining tandem systems, some studies have bypassed the intermediate separation process and fed a CO_2_/CO mixture directly into the eCORR step, thereby simplifying system design and exploiting the potential kinetic benefits of CO_2_/CO cofeeds. Notably, the use of mixed CO_2_/CO cofeeds has been shown to significantly enhance C_2_H_4_ production. Detailed analysis reveals that this enhancement primarily arises because CO does not compete with CO_2_ for the same active sites; instead, each molecule binds to distinct and nonoverlapping sites on Cu catalysts, with an additional cross-coupling pathway occurring between the CO_2_ and CO specific active sites.^64,104,105^ Other studies have reported that the presence of CO_2_ promotes eCORR, and that at least two distinct types of Cu sites exist, one (Cu_CO2_) more active for CO_2_-to-CO conversion, and the other (Cu_CO_) favoring the further reduction of CO to C_2+_ products.^55^ These mechanistic insights underscore the synergistic effect of CO_2_/CO cofeeding and highlight its promise for achieving higher efficiency in tandem electrolyzers eCO_2_RR.^101^ But while considering the challenges of carbonate formation and carbon loss, the operational priority remains to maximize CO concentration in the output stream while minimizing residual CO_2_. Therefore, a holistic evaluation that integrates the upstream CO_2_-to-CO electrolyzer with the downstream CO-to-C_2+_ reactor is essential to capture the true efficiency and economic potential of tandem electrolyzers eCO_2_RR.

The modular design of tandem electrolyzers systems represents a transformative step toward scalable and commercially viable carbon utilization.^50^ By separating complex reactions into independently optimized units, these systems provide the flexibility to adapt each module to specific feedstocks, intermediates, or product demands. Such architecture not only improves process control and eEE but also allows for straightforward troubleshooting, maintenance, and system upgrades, a critical advantage for industrial deployment. As research moves from laboratory demonstrations to pilot and production scales, modular tandem systems can be readily expanded or reconfigured to meet increasing throughput requirements. This plug-and-play nature accelerates integration with existing renewable energy infrastructure, making it possible to match production capacity with intermittent power supplies or changing market needs. Additionally, modularity enables parallelization of multiple production lines, reducing downtime and risk while supporting continuous operation.

Beyond technical scalability, modular tandem systems open new opportunities for decentralized chemical manufacturing and point-source CO_2_ capture and conversion. This flexibility supports a more distributed, resilient, and sustainable chemicals industry, bridging the gap between current scientific advances and real-world impact. Looking ahead, the continued evolution of tandem system design, coupled with advances in materials, automation, and digital process control, positions this technology at the forefront of the next generation of carbon management solutions.

## 3 Tandem Electrolyzers System Architectures

### 3.1 Electrolyzer Types

#### 3.1.1 H-Cell

The H-cell remains the most fundamental and widely adopted configuration for early-stage eCO_2_RR. Structurally, the H-cell consists of two separate compartments, one for the cathode and one for the anode, typically divided by an ion-exchange membrane (Figure 6a). This straightforward design allows for the facile study of catalyst materials, electrolyte composition, and reaction conditions under well-defined, batch-mode conditions.

**Figure 6:**
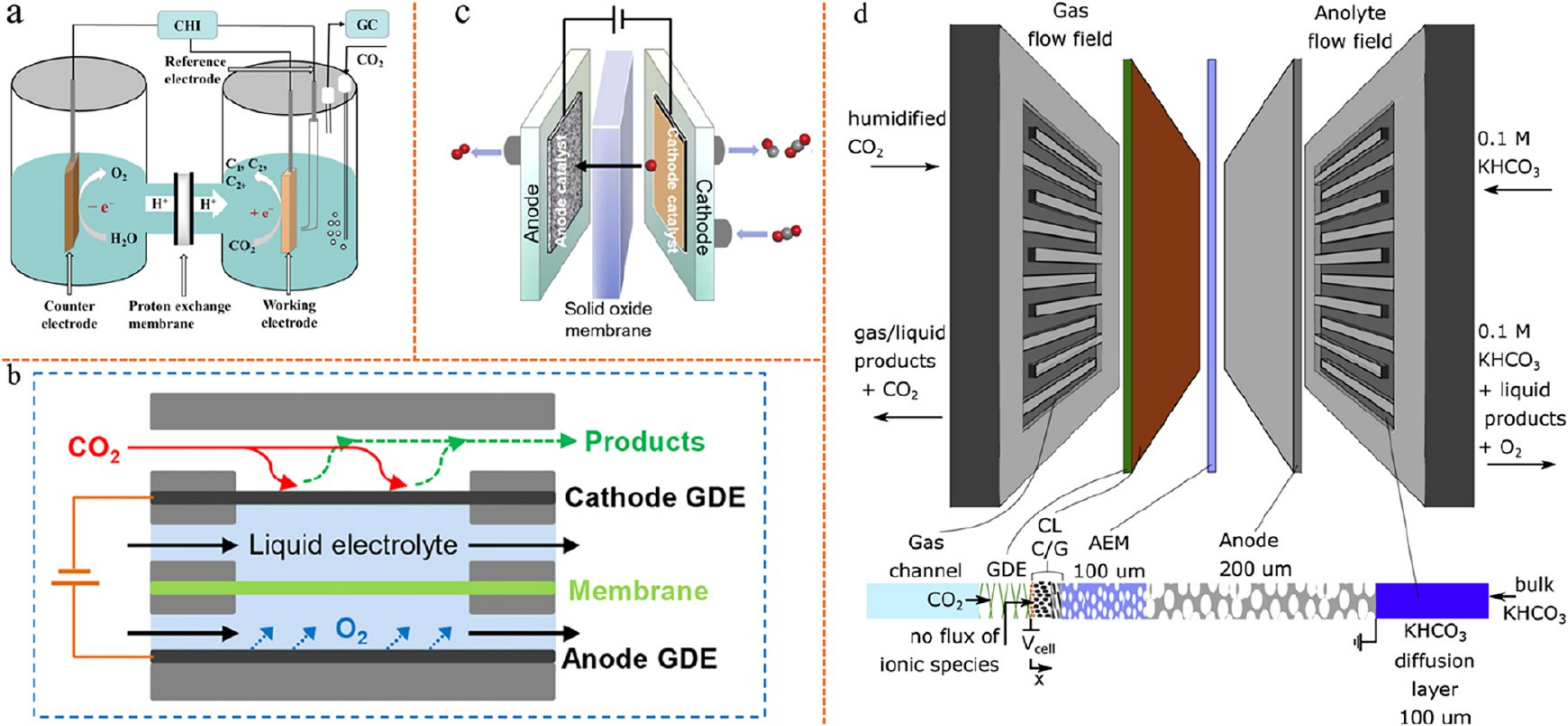
Representative electrolyzer types for electrochemical CO_2_ reduction. (a) Traditional H-cell setup employing a proton exchange membrane for ion transport between the cathode and anode compartments. Reproduced from ref 106. Copyright 2023 MDPI. (b) Flow cell configuration illustrating separate cathode/anode GDEs and liquid electrolytes. Reproduced from ref 107. Copyright 2020 Peking University Press. (c) A SOEC configuration, featuring separated anode and cathode catalysts divided by a solid oxide membrane. Reproduced from ref 108. Copyright 2023 Elsevier. (d) Model schematic showing different layers of the MEA system. Reproduced from ref 109. Copyright 2021 Cell Press.

In the context of tandem eCO_2_RR, H-cells are rarely used and offer limited practical value for most advanced system designs. Their simple configuration, while suitable for catalyst screening and proof-of-concept studies, does not align with the strengths of tandem strategies, which benefit from integrating already reported catalysts into functionally coordinated, energy-efficient multistep processes. The H-cell’s design is also limited by low mass transport rates and restricted current densities due to long diffusion paths and poor gas reactant accessibility, hindering both scalability and practical relevance for industrial applications where high current densities and continuous operation are required. As research in this field advances, the focus has shifted away from H-cells toward more sophisticated electrolyzer types, such as flow cells and MEAs, that enable higher performance and better scalability. However, for certain novel proof-of-concept experiments, such as the tandem production of carbonate-derived ethylene, or specialized processes like high-purity ethylene synthesis from 2-bromoethanol (2-BrEtOH), H-type cells can still offer practical advantages due to their ease of assembly and operation and intrinsic structure design. Nonetheless, for established pathways such as CO_2_-to-CO and CO-to-C_2+_ conversions, more advanced electrolyzer types should be prioritized for their superior performance and scalability.

#### 3.1.2 Flow Cell

Flow cells have become the predominant electrolyzer configuration for eCO_2_RR in recent years, owing to their efficient mass transport at the gas–liquid–solid triphase boundary (TPB), which facilitates high reaction rates through effective CO_2_ delivery and rapid product removal (Figure 6b). Some early demonstrations of tandem eCO_2_RR systems have utilized flow cell designs in one or both steps, capitalizing on their ability to operate at industrially relevant current densities and utilizing highly alkaline electrolytes. However, as research has progressed and higher eEE has been targeted, the inherent limitations of flow cells have become more apparent, most notably the unavoidable significant ohmic losses. The use of flowing liquid electrolytes between electrodes introduces substantial ohmic resistance in the cell. For example, a 3 mm layer of 1 M KOH results in an IR drop of approximately 800 mV at a current density of 500 mA/cm^2^.^110^ These losses lead to elevated overall cell potential and consequently reduced eEE, which presents a fundamental challenge for this electrolyzer type. Despite these limitations, flow cells remain a cornerstone of eCO_2_RR research. They are especially valued for catalyst screening, mechanistic studies, and benchmarking new materials, owing to their simple operation, ability to achieve industrially relevant current densities, and the ease of comparing cathodic CO_2_ reduction potentials against reference electrodes. These attributes make flow cells highly suitable for individual step optimization such as catalyst development, reaction parameter selection. While alternative electrolyzer types are now being explored to address eEE and scalability challenges, flow cell systems continue to play a critical role in the fundamental investigation.

Additionally, Figure 7a presents the results of a sensitivity analysis for a flow cell operating under alkaline conditions without CO_2_ loss to carbonate formation. Each plotted line showing the cost of ethylene as one input parameter is independently scaled on the *x*-axis from 10 to 200% of its initial value. The “center” cost, at 100%, is the same for all lines and is labeled in the legend. Of note, SPCE is defined as (CO_2_ reduced to any product)/(unreacted CO_2_ at the cathode output + CO_2_ reduced to any product). Even though this analysis is based on an alkaline flow cell, which is a less favorable configuration for CO_2_ conversion, it still demonstrates that both system lifetime and SPCE have minimal impact on the overall cost of the electrolyzer under investigation, provided that the lifetime exceeds approximately 10 years and the SPCE reaches at least 20%. These results provide further evidence supporting the previous conclusions. Concerns regarding the additional capital investment required for tandem electrolyzer systems are alleviated, as the long operational lifetimes of electrolyzers significantly mitigate their overall cost contribution. Similarly, the minimal impact of SPCE on system cost is attributable to the relatively low expense of recovering unreacted CO_2_ via the cathode separation module, which is more economical than purchasing additional CO_2_ feedstock. At high current densities (∼1 A/cm^2^), improving electrolyzer eEE, by reducing cell voltage and increasing Faradaic efficiency (FE), as well as lowering electricity price, are all effective strategies to reduce the cost of ethylene production. These findings provide valuable insights for improving the eCO_2_RR system, such as lowering cell voltage through the use of zero-gap MEA.

**Figure 7:**
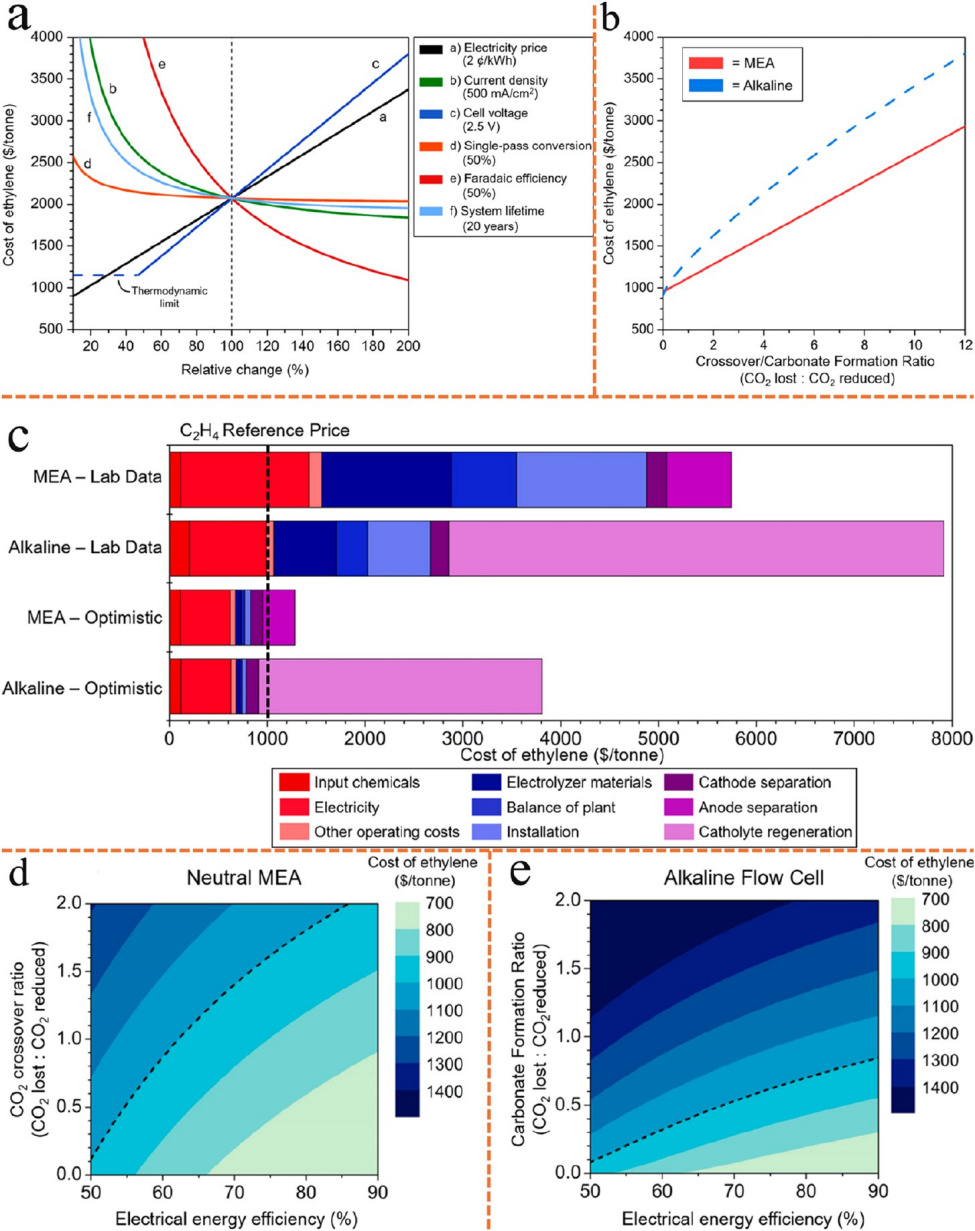
General economic trends of eCO_2_RR electrolyzer and CO_2_ reduction in a neutral MEA and an alkaline flow cell. Reproduced from ref 95. Copyright 2021 American Chemical Society. (a) Sensitivity analysis of an alkaline flow cell with no CO_2_ loss to carbonate formation. (b) Ethylene production costs in neutral MEA and alkaline flow cells with varying crossover/carbonate formation ratios while all other cell inputs are fixed at their optimistic values. (c) Cost of ethylene production breakdown for both systems. (d, e) Cost of ethylene produced in a neutral MEA and alkaline flow cell, respectively, with variable crossover ratio/carbonate formation ratio and eEE.

#### 3.1.3 Solid Oxide Electrolysis Cell

SOECs have become an increasingly prominent configuration for high-temperature eCO_2_RR, especially for CO production. Compared with low-temperature systems, SOECs operate at 600–900 °C, leveraging fast electrode kinetics and enhanced ionic conductivity in solid electrolytes to achieve high current densities, near 100% FE, and excellent stability-features that make them attractive for practical and industrial applications (Figure 6c). Notably, SOECs enable direct use of pure CO_2_ feedstocks, producing CO with essentially complete selectivity, and are now regarded as one of the most promising routes for large-scale CO_2_ conversion.

In the context of tandem electrolyzers eCO_2_RR, SOECs stand out for their ability to efficiently convert CO_2_ into concentrated CO without byproducts, making them an excellent front-end component for integrated systems. By supplying high-purity CO feedstock at industrially relevant rates, SOECs enable subsequent low-temperature electroreduction steps, such as MEAs to focus on the synthesis of valuable C_2+_ products. One of the principal advantages of SOECs over flow cells or MEAs for the initial CO_2_-to-CO conversion is their complete suppression of carbonate formation and associated carbon loss. Because SOECs operate without aqueous electrolytes, CO_2_ does not react with hydroxide ions or dissolve into the electrolyte, eliminating a major efficiency bottleneck found in liquid-phase systems. This fundamental distinction not only makes SOECs uniquely suited for tandem electrolyzer strategies but also enables higher energy efficiencies due to minimized ohmic losses and superior ionic conductivity at elevated temperatures. Moreover, SOECs offer straightforward product separation, as the only inputs are CO_2_ and electricity, and the only products are CO and O_2_, with virtually no side reactions. These advantages highlight SOECs as a powerful platform for supplying pure CO in advanced tandem electrolyzers eCO_2_RR system.

However, the high operating temperatures required by SOECs, typically in the range of 700–900 °C, also introduce new challenges. Such harsh reaction conditions can lower overall eEE and raise concerns related to material stability, reactor durability, and system integration, especially when coupling high-temperature SOECs with low-temperature downstream reactors. Developing low-temperature SOEC techniques would represent a major breakthrough, combining the advantages of solid oxide operation with milder conditions. Despite their promise, SOECs still face technical hurdles including the long-term stability of cathode materials, compatibility with intermittent renewable electricity, and seamless integration into practical tandem systems. Ongoing advances in electrode material design, catalyst engineering, and system architecture will be essential to unlock the full potential of SOEC-driven tandem eCO_2_RR.

#### 3.1.4 Membrane Electrode Assembly Electrolyzer

MEAs have rapidly become the benchmark for advanced eCO_2_RR systems, especially as the field moves closer to industrial relevance. Unlike traditional H-cell and flow cell setups, MEAs employ a zero-gap architecture: a thin polymer electrolyte membrane is sandwiched directly between the cathode and anode. In Figure 6d, the one-dimensional domain models the MEA system from the CO_2_ entering the GDE through to the wetted Cu catalyst layer, the carbon/graphite layers, the membrane (AEM), the anode, and the electrolyte diffusion layers. This compact configuration eliminates the need for a bulk liquid electrolyte between electrodes, dramatically reducing ohmic losses and enabling much higher current densities at lower cell voltages. The direct supply of gaseous CO_2_ to the cathode catalyst layer, often delivered through a carefully engineered GDE, ensures efficient reactant delivery and robust three-phase contact. As a result, MEA systems routinely reach current densities well above 200 mA/cm^2^, meeting or exceeding the thresholds required for commercial viability. The elimination of catholyte prevents direct contact between the CO_2_ feed and the bulk alkaline reservoir, thereby significantly reducing carbonate formation and, in turn, lowering the costs associated with electrolyte regeneration and CO_2_ recovery. The steep slopes of both lines in Figure 7b indicate that crossover/carbonate formation are major contributors to the final cost of ethylene. Depending on the extent of carbonate formation, CO_2_ recovery can represent the largest single cost in the entire system. In contrast, the MEA system is less affected by these losses.

However, the unique advantages of MEA also introduce several significant challenges. In order to minimize ohmic losses, the catholyte is eliminated and the membrane, catalyst layer, and gas diffusion layer (GDL) are pressed tightly together, typically forming a zero-gap structure that must withstand substantial mechanical pressure. The absence of catholyte also means that ion and reactants’ transport must be carefully managed, effective water management becomes critical. Excess water can lead to flooding, damaging the GDE and reducing performance, while insufficient water risks membrane dehydration and failure, since MEA membranes are fragile and require a humid environment to maintain integrity. Additionally, the removal of the catholyte exposes one side of the membrane directly to a gas/liquid mixture and the other to a strong acidic or basic solution, imposing stringent chemical and mechanical requirements on the membrane. The zero-gap design and high current density operation under low overpotential demand a thin membrane to lower the ohmic resistance and facilitate efficient ion transport, but this also makes the membrane more susceptible to mechanical failure. Tight compression is necessary to ensure proper contact between the membrane and electrodes, yet uneven pressure or pressure differentials during extended high-current operation can further increase the risk of rupture. Collectively, these factors create extremely high demands for membrane performance and long-term durability in MEA-based systems. Recent advances in membrane materials and device engineering have significantly improved operational durability. While membrane degradation was once considered a major limitation for eCO_2_RR, recent studies have demonstrated that modern membranes can sustain stable operation for thousands of hours even under high current densities.^111^ As a result, membrane durability is no longer regarded as the primary bottleneck for long-term eCO_2_RR performance. The ion-exchange membrane discussed here in MEA refers to an AEM. For other types of membranes used in eCO_2_RR, such as cation exchange membranes (CEM) and bipolar membranes (BPM), their roles in addressing carbon loss and carbonate formation in acid eCO_2_RR and related systems, as well as their performance differences and respective pros and cons, are presented in Section 6.1.3.

Today, flow cells and MEAs are the most widely used electrolyzer types in eCO_2_RR. While flow cells offer high reaction rates and are robust thanks to durable membranes, they suffer from significant ohmic losses caused by the presence of a liquid electrolyte, which results in higher cell potential and reduced eEE. This persistent limitation has fueled a shift toward MEA architecture. In MEAs, the zero-gap design effectively minimizes ohmic losses and significantly enhances system efficiency, a critical advantage as the field moves toward commercialization. However, MEAs introduce their own challenges, such as complex water management and increased sensitivity to mechanical and chemical stresses due to the fragile membrane and tightly coupled cell components. Despite these challenges, the superior eEE of MEA systems makes them a more promising pathway for industrial adoption compared to traditional flow cells. While the membranes used in flow cell designs are generally robust and tolerant of operational stresses, the ohmic losses introduced by the liquid electrolyte represent a fundamental limitation. In my opinion, this issue is difficult to overcome through engineering solutions alone, making it a persistent barrier to achieving high eEE in flow cell systems. Although flow cells remain valuable for catalyst screening and fundamental research, overcoming the intrinsic ohmic losses in flow cell designs is far less tractable than addressing the engineering complexities of MEAs, justifying continued investment and development in MEA technology for future commercialization.

A closer examination of the TEA between flow cells and MEAs is provided in Figure 7c, which highlights the significant impact of CO_2_ loss to carbonate formation in alkaline environments. Red shaded bars show the contribution to electrolyzer operating costs, blue shaded bars show electrolyzer capital costs, and purple shaded bars represent costs of external systems. In both laboratory data and optimistic scenarios for alkaline flow cell, the cost of catholyte regeneration accounts for over 60% of the total production cost. The elevated operating and capital costs observed for the laboratory-scale MEA are primarily attributed to high cell potential because of the low conductivity neutral electrolyte, which results in very low eEE. However, since these data are based on single-cell eCO_2_RR systems, replacing them with tandem configurations, where a highly alkaline electrolyte is employed in the second-step eCORR to ethylene, can substantially reduce cell potential and enhance eEE. While at the laboratory scale the electricity and electrolyzer costs are lower for alkaline flow cells, these savings are offset by the expenses associated with electrolyte regeneration. This analysis suggests that, for the first cell, it is essential to minimize carbonate formation by utilizing an MEA rather than a flow cell. Although the comparison here is between an alkaline flow cell and a neutral MEA, employing a neutral flow cell would result in significantly higher resistance compared to the MEA, leading to increased cell voltages, higher electricity costs, and overall poorer performance. Additionally, the cost associated with separating CO_2_ that crosses over in the MEA is represented by the “anode separation” component. Encouragingly, as discussed later in Section 6.1, new techniques enable efficient recycling of crossover CO_2_. Under optimistic conditions, MEA tandem systems have the potential to achieve ethylene production costs lower than the current market price.

Figure 7d,e further examines the requirements for achieving ethylene production near profitability, assuming that CO_2_ loss via crossover or carbonate formation is minimized. All inputs are fixed at their optimistic values, while full-cell voltage and CO_2_ crossover/carbonate formation are varied. CO_2_ crossover ratio/carbonate formation ratio is defined as the number CO_2_ molecules that cross over/are lost to carbonate for each CO_2_ molecule that is reduced to any product. In all plots, the dotted black lines indicate ethylene’s reference price ($1000/tonne). Each plot utilizes optimistic input parameters, varying both the electrolyzer eEE and the extent of CO_2_ loss, to estimate the final cost of ethylene. The results indicate that MEA systems can more readily achieve ethylene production costs below $1000 per tonne, by eliminating CO_2_ loss, an ambitious goal that is now attainable through advanced MEA architecture and tandem system designs. However, with increasing carbonate formation and CO_2_ crossover, the eEE threshold needed for economic feasibility rapidly exceeds achievable levels. Thus, for single-step electrolyzers to produce ethylene profitably, CO_2_ loss to carbonate formation must be entirely eliminated, a significant challenge for single-cell systems. Even in neutral MEAs, while CO_2_ crossover can be addressed, the low conductivity and low alkalinity of the neutral electrolyte tend to decrease selectivity and increase cell voltage. All in all, the zero-gap structure MEA still represents the state-of-the-art electrolyzer type for tandem eCO_2_RR systems.

For the tandem electrolyzer strategies introduced in this review, particularly in terms of electrolyzer configuration, it is suggested that the zero-gap MEA be employed in both the first and second cells to overcome the limitations mentioned above. In this design, the first MEA cell operates with a neutral electrolyte for efficient CO_2_-to-CO conversion, followed by a second MEA cell operating in a highly alkaline environment for the subsequent conversion of CO to C_2+_ products. In both cases, the MEA is utilized as the optimal electrolyzer type, owing to its low ohmic losses and minimized carbon loss. This approach provides a clear pathway from fundamental research to scalable, commercial technologies. Furthermore, the modular and stackable nature of MEA designs supports future industrial deployment, aligning with the broader goals of sustainable carbon utilization.

### 3.2 Catalyst Selection and Pairing

In tandem electrolyzers systems, careful pairing and selection of catalysts for each step is central to achieving high efficiency, selectivity, and system stability. Unlike single-cell approaches, tandem architecture offers the unique advantage of decoupling reaction environments and optimizing each catalyst specifically for its target transformation.

#### 3.2.1 First Cell Catalysts

Typically, the first cell in a tandem system is dedicated to converting CO_2_ to a key intermediate, most often CO, using catalysts such as Ag, Au, or advanced single-atom transition metals supported on carbon. Considering performance, technological maturity, and cost, Ni-based single-atom catalysts and Ag materials are currently the most widely used options in the first cell of tandem systems (see Table 1). These materials are selected for their high selectivity and activity in the CO_2_-to-CO reaction, as well as their stability under the relevant electrolyte conditions.^112^ For some emerging tandem system designs and target products, such as high-purity C_2_H_4_, ethylene carbonate, and succinic acid, the first cell is required to convert CO_2_ directly to C_2_H_4_, making Cu-based catalysts the preferred choice in these cases.

**Table 1:** Configuration and Performance of the First Cell^a^

ref		Target Product	Electrolyzer	Catalyst	Catalyst Type^b^	Electrolyte	*j* ^c^ (mA/cm^2^)	FE (%)
Theaker^113^		CO	Flow cell^d^	Ag-NC foil	Reported	0.1 M KHCO_3_	2	80
Ozden^99^		CO	SOEC	/	/	/	550	95
Wu^114^		CO	Flow cell	3D Ni-SAG	Reported	0.5 M KHCO_3_	140	95.9
Li^115^		CO	MEA	Ni–N–C	Reported	0.5 M K_2_SO_4_ + H_2_SO_4_ anolyte with pH 0.5, 0.5 MPa CO_2_	200	/
Möller^64^		CO	MEA	Ni–N–C	Reported	0.1 M KHCO_3_	Various	∼100
Crandall^50^ ^e^	Tandem data	CO	MEA	Ag particles	Commercial	100 mM CsHCO_3_	100	/
	Individual scale-up		MEA (Stacks)				200 (100 A Total)	>80
Wu^101^		CO	MEA	Ni-CN-3DF	Original	0.5 M KHCO_3_	200	98
Ni^84^		2-BrEtOH	Flow cell	Cu/Cu_2_O	Original	Catholyte: 1 M KOH	138.8 (Partial)	46.1
						Anolyte: 1 M KBr		
Wen^85^		C_2_H_4_	MEA	Cu-PTFE	Reported	0.1 M KHCO_3_	130	47
Weidner^116^		CO	Flow cell	NiCu single-atom	Reported	1 M KOH	400	∼97
Cai^86^		C_2_H_4_	Flow cell	Cu(OH)F nanoparticle	Reported	Catholyte: 2.0 M KOH	100	63 ± 4
						Anolyte: 1.0 M KOH		
Mao^87^		C_2_H_4_	Flow cell	CuO	Original	1 M KOH	337.5 mA (Total)	50
Wang^117^		CO	Flow cell	Ag NCs@Ag-MOF	Original	1.0 M KOH	/	99.1

a All data in this table corresponds to results from integrated tandem electrolyzer systems rather than individual optimization experiments. Only one study has reported the full-cell eEE of the first cell in such systems: Ozden et al.[Bibr ref99] (2021), with a value of 86% (considering electricity only). In addition, only two studies have reported the SPCE of the first cell: Ozden et al.[Bibr ref99] (2021) and Crandall et al.[Bibr ref50] (2024), with values of 48 and ∼40%, respectively. Due to the limited number of reported values, these data are not listed in the table.

b Three catalyst types are listed in all the tables to indicate the source of each catalyst. “Commercial” refers to catalysts that are commercially available. “Reported” indicates catalysts that were reproduced in this study based on previously reported work. “Original” denotes catalysts that were developed originally in this article.

c *j* denotes total current density, expressed in mA·cm^–2^.

d This flow cell represents the initial version, where the liquid electrolyte flows through the electrode and is saturated with CO_2_ in an external electrolyte reservoir. No GDE is used, making it very similar to an H-cell, except the electrolyte is flowing.

e All data presented in the tables are obtained from real integrated tandem electrolyzer systems, unless otherwise stated. Crandall et al.[Bibr ref50] (2024) is the only study that demonstrated a real integrated tandem electrolyzer system while simultaneously scaling up both individual steps of the tandem process.

#### 3.2.2 Second Cell Catalysts

The second cell is equipped with a catalyst specifically designed for the further reduction of the intermediate (typically CO) to multicarbon products. Copper-based catalysts are most widely used for this purpose, owing to their unique capability to promote carbon–carbon coupling and facilitate the formation of valuable chemicals such as ethylene, ethanol, and n-propanol. In fact, across the tandem systems reviewed, all second-step CO-to-C_2+_ catalysts are exclusively Cu-based (see Table 2). For other novel target products, however, catalyst selection is more diverse and tailored to the unique reactants and reaction pathways involved, with no universal approach currently established.

**Table 2:** Configuration and Performance of the Second Cell[Table-fn t2fn1]

ref	Feed	Target Product	Electrolyzer	Catalyst	Catalyst Type	Electrolyte	*j* (mA/cm^2^)	FE (%)
Theaker^113^	∼80% CO/20% H_2_	C_2_H_5_OH	Flow cell^b^	OD-Cu foil	Reported	0.1 M KOH	1–2	28.4
Ozden^99^	∼CO[Table-fn t2fn2]	C_2_H_4_	MEA	Cu:Py:SSC	Original	3 M KOH	120	/
Wu^114^	∼CO[Table-fn t2fn2]	n-propanol	Flow cell	Multihollow Cu_2_O NPs	Reported	1.0 M KOH	42.5	28.0
Li^115^	∼CO[Table-fn t2fn2]	C_2_H_4_	MEA	Cu Nanoparticle	Commercial	0.5 M KOH	1000	46
Möller^64^	18% CO	C_2+_	Flow cell	Spherical Cu particles	Commercial	1.0 M KHCO_3_	/	/
	35% CO							
Crandall^50^	∼CO[Table-fn t2fn2]	Acetate	MEA	Cu particles	Commercial	2 M KOH	100	∼50
	Pure CO		MEA (Stacks)				300 (300 A Total)	30–35% purity >96
Wu^101^	74.8% CO	C_2+_	MEA	Cu_3_N-HDD	Reported	/	800	93.5[Table-fn t2fn3]
Ni^84^	Liquid[Table-fn t2fn4]	C_2_H_4_	H-cell	AC-Ag/C	Original	0.5 M KCl	/	>95
Wen^85^	(C_2_H_4_)[Table-fn t2fn5]	2-BrEtOH	H-cell	WO_3_ nanoarrays	Original	0.5 M KBr	/	/
Weidner^116^	10.3% CO	C_2_H_5_OH	Flow cell	Al-Rich Cu/CuO_*x*_	Original	1 M KOH	300	/
Cai^86^	(C_2_H_4_)[Table-fn t2fn5]	EC	Batch cell	IrO_2_-DSA	Reported	0.5 M KBr...^f^	6	37
Mao^87^	(C_2_H_4_)[Table-fn t2fn5]	SA	Single-cell	FeNi foam	Commercial	DMF...^g^	/	/
Wang^117^	∼CO[Table-fn t2fn2]	Ethanol&Acetate	Flow cell	Cu–O_2_N_2_–COF	Original	1.0 M KOH	120	90.9

f All the data presented in this table are collected from experimental results of the second cell in integrated tandem electrolyzer systems. No studies have reported the full-cell eEE of the second cell in integrated tandem electrolyzer systems; therefore, these data are not listed in the table. Only one study, Theaker et al.[Bibr ref113] (2018), has reported the SPCE of the second cell in such a system, with a value of 6.4%. Due to the limited availability of data, these values are also not listed here.

g Because the results are obtained from real integrated tandem electrolyzer systems, the feed gas to the second cell is not pure CO. After the intermediate CO_2_ removal and purification process, the feed consists predominantly of CO (after purification), with small amounts of byproduct H_2_ or minor unspecified components such as water vapor.

h This FE result should be interpreted with caution, as its calculation did not account for the CO_2_/CO cofeed factor.

i In this real integrated tandem electrolyzer system for indirect CO_2_-to-ethylene conversion, the first cell produces an anolyte containing the intermediate 2-BrEtOH, which serves as the feed for the second cell.

j The results are obtained from real integrated tandem electrolyzer systems, in which the gas exiting the first cell is directly fed into the second cell without further processing and contains C_2_H_4_ as the useful component.

k The optimal electrolyte composition was 0.5 M KBr, 0.2 M Cs_2_CO_3_, and 3 mM K_2_Cr_2_O_7_ in 30:70 vol % DMF/water.

l Typically, the electrolyte of 0.5 mM *n*-Bu_4_NPF_6_ in 8 mL DMF was used in a single electrochemical cell.

#### 3.2.3 General Principles of Catalyst and Electrolyzer Selection

Notably, the catalyst choices in typical tandem system designs are often drawn from existing or previously reported materials, rather than from the development of entirely new, highly sophisticated catalysts specifically for tandem applications. In most CO_2_-to-C_2+_ tandem routes, both the CO_2_-to-CO and CO-to-C_2+_ steps are already well-studied and rapidly advancing fields. This provides a wealth of readily available catalysts and underlines one of the core advantages of the tandem eCO_2_RR strategy: by decoupling a complex transformation into two more manageable steps, each stage, catalyst, reactor, electrolyte, can be individually optimized. Few current research either focus on developing improved CO_2_-to-CO catalysts while using established Cu-based catalysts for the second CO-to-C_2+_ step or leverage mature CO_2_-to-CO catalyst technology and introduce a novel catalyst for CO reduction. Simultaneous development of new catalysts for both steps is rare and generally unnecessary, as the primary objective of tandem electrolyzers design is not academic novelty but rather reduced energy consumption, lower costs, and enhanced prospects for industrialization and commercialization.

Beyond conventional eCO_2_RR-to-C_2+_ products, recent advances have targeted specialty chemicals such as high-purity ethylene, ethylene carbonate, and succinic acid. These systems typically employ a mature CO_2_-to-C_2_H_4_ process in the first cell, followed by development or careful optimization of the downstream catalyst or reaction environment to convert C_2_H_4_ or intermediates like 2-BrEtOH into higher-value products. This trend further demonstrates the benefits of the tandem electrolyzer strategies, enabling the replacement of energy-intensive, fossil-fuel-based production routes with renewable, electricity-driven pathways.

Finally, the reported tandem electrolyzer configurations and performances are summarized in Table 3. Apart from electrolyzer type and catalyst selection, the performance metrics for tandem electrolyzer systems are often messy and inconsistent, particularly when considering the overall tandem system rather than individual steps or cells, as shown below. In some studies, no meaningful metrics are reported; while this may be acceptable for proof-of-concept experiments, standardized, comprehensive, and instructive performance metrics should be adopted in the future. This inconsistency represents a significant challenge, which is further discussed in Sections 5 and 6.4.

**Table 3:** Tandem Electrolyzer Configuration and Performance

ref	Conversion	Electrolyzers Configuration	Performance
Theaker[Bibr ref113] (2018)	CO_2_-to-C_2_H_5_OH	Two old-type Flow cells	FE of 11.0%
Ozden[Bibr ref99] (2021)	CO_2_-to-C_2_H_4_	SOEC + MEA	eEE of 20%, SPCE of 11%, Stability of 40 h, Energy Intensity of 138 GJ·ton^–1^
Wu[Bibr ref114] (2022)	CO_2_-to-n-propanol	Flow cell + Flow cell	FE of 15.9%
Li[Bibr ref115] (2023)	CO_2_-to-C_2_H_4_ & C_2+_	MEA + MEA	The highest ethylene and C_2+_ FEs are 46 and 99%
Möller[Bibr ref64] (2023)	CO_2_-to-C_2+_	MEA + Flow cell	SPCE of 30–35%/
Crandall[Bibr ref50] (2024)	CO_2_-to-Acetate	MEA + MEA	Target purity of ∼99%, Stability of 200 h
Wu[Bibr ref101] (2024)	CO_2_-to-C_2+_	MEA + MEA	/
Ni[Bibr ref84] (2024)	CO_2_-to-C_2_H_4_	Flow cell + H-cell	Target purity of 98.00 ± 1.45 wt %, 2 + 6 (Hours)
			Energy Intensity of 310.99 GJ·ton^–1^
Wen[Bibr ref85] (2024)	CO_2_-to-EC	MEA + H-cell + Flask	/
Weidner[Bibr ref116] (2025)	CO_2_-to-C_2_H_5_OH	Flow cell + Flow cell	Production rate of ethanol formation at a current density of 300 mA/cm^2^ increased by 28%
Cai[Bibr ref86] (2025)	CO_2_-to-EC	Flow cell + Batch cell	Cost of USD $2326/ton
Mao[Bibr ref87] (2025)	CO_2_-to-SA	Flow cell + Single-cell	0.12 μmol SA was obtained in 5 h of catalysis
Wang[Bibr ref117] (2025)	CO_2_-to-Liquid C_2_	Flow cell + Flow cell	FE of liquid C_2_ products (ethanol and acetate) was 90.9% at −0.98 V (vs RHE) at partial current density of 120 mA/cm^2^

### 3.3 Feedstock Management and Reaction Environment

#### 3.3.1 CO_2_/CO Co-Feed Ratio

A representative earlier example of tandem-electrolyzer-based eCO_2_RR toward C_2+_ products was demonstrated by Romero Cuellar et al.,^118^ who developed a two-step flow cell system operating at industrially relevant current densities. In this configuration, CO_2_ was first reduced to a CO-rich gas mixture using a silver-based GDE, followed by further reduction on a copper-based cathode to yield C_2_ and C_3_ products. Initially, the system exhibited limited FE (approximately 20%) for C_2+_ products due to residual CO_2_ diluting the CO feed in the second stage. However, by incorporating a CO_2_ scrubber between the two cells and independently controlling the current in each step, the authors achieved a significantly improved C_2+_ selectivity of 62% at a current density of 300 mA/cm^2^. This result clearly demonstrates that eliminating the dilution effect of CO_2_ can significantly enhance FE in tandem configurations. It is also worth noting that this study included only a limited number of data points, particularly comparing just two cases, with and without the CO_2_ scrubber. This leaves open the possibility that intermediate CO_2_/CO ratios (between 0 and 1) could further promote C–C coupling and optimize product selectivity.

Wu^119^ and colleagues began by investigating the CO_2_ reduction and CO reduction performance of Cu catalyst in the second cell of the tandem system. Under strong alkaline conditions (1 M KOH), the Cu catalyst exhibited a marked inclination toward the HER during CO_2_RR, with H_2_ selectivity consistently above 10% and reaching around 20% at all tested current densities. The FE for C_1_ products such as CO, CH_4_, and HCOOH also remained above 10%. These side reactions made it challenging to achieve a C_2+_ FE above 80%, with the highest observed value being 75.8% at 500 mA/cm^2^. In contrast, under eCORR conditions, the C_2+_ FE consistently exceeded 80% across a wide range of current densities (100 to 1200 mA/cm^2^) and peaked at 92.9% at 1 A/cm^2^. The authors attributed this substantial difference to increased CO surface coverage during eCORR, which blocks active sites for HER and favors C–C coupling. Another important factor is that using CO as the only reactant eliminates pathways to other C_1_ products, further boosting selectivity for C_2+_ products. These findings underscore the advantage of the segmented tandem system: efficient CO production in the first cell, followed by using high-purity CO in the second cell, creates optimal conditions for C_2+_ synthesis by simulating an ideal eCORR environment. In addition to experiments using pure CO_2_ or CO feeds, Wu and colleagues examined the effect of varying the CO_2_/CO ratio by adjusting the initial CO_2_ flow rate into Cell-1 (Figure 8a). At a fixed current, reducing the CO_2_ flow rate resulted in a higher CO concentration in the output. Without any intermediate gas purification, the study circumvents the issue of residual CO_2_ affecting FE in Cell-2 by assuming that, under the highly alkaline conditions present, any unreacted CO_2_ will be rapidly adsorbed and precipitate as bicarbonate on the cathode surface. In reality, the presence of CO_2_ in the CO stream from Cell-1 makes it impossible to accurately calculate FE in Cell-2, as the participation of CO_2_ in downstream reactions disrupts standard FE calculations. However, if we set aside this complication and accept the authors’ approach, the reported results indicate a clear trend: higher CO concentration from Cell-1 leads to increased FE for C_2+_ products in Cell-2 (Figure 8b). Nevertheless, this study clearly demonstrates the practicality of a single-pass tandem electrolyzers operating without intermediate purification. The system achieves outstanding carbon selectivity toward C_2+_ products, strongly supporting its potential for efficient multicarbon product synthesis.

**Figure 8:**
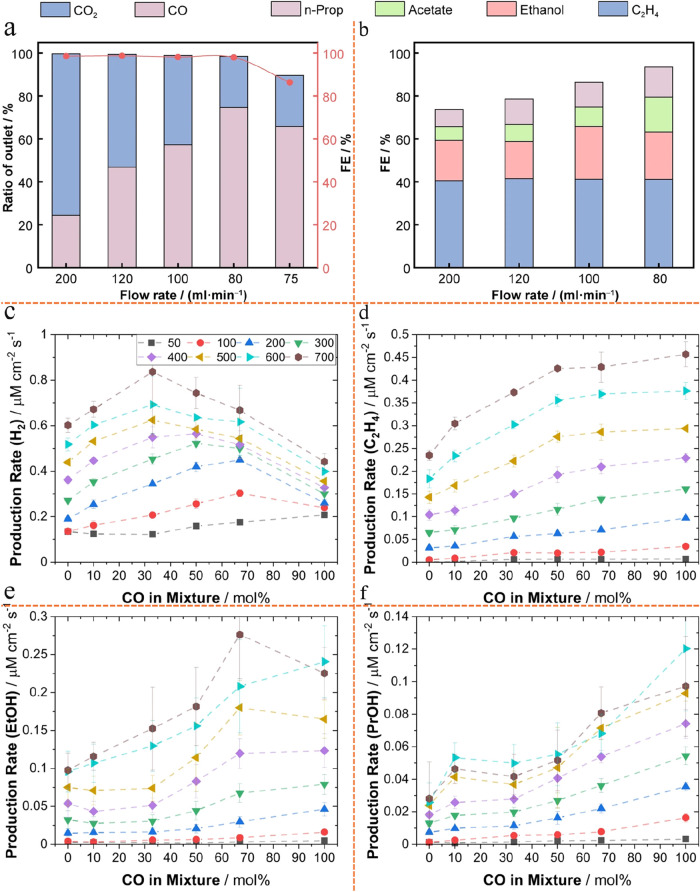
Gas composition of Cell-1 outlet and C_2+_ FE of Cell-2 with variation of Cell-1’s inlet flow rate. Influence of CO_2_/CO cofeed composition on production rates observed for electrolysis in single-cell experiments of Cell-2. (a) Gas composition of Cell-1 outlet and its FE, varying with inlet flow rate. Reproduced from ref 101. Copyright 2024 Springer Nature. (b) C_2+_ FE with variation of Cell-1’s inlet flow rate. Reproduced from ref 101. Copyright 2024 Springer Nature. (c–f) Production rates are shown for (**c)** hydrogen, (**d)** ethylene, (**e)** ethanol, and (**f)** n-propanol.^64^ Production rates of key products using a single-electrolyzer cell as a function of CO mol percent (mol %) in the CO_2_/CO cofeed, measured for various applied current densities in Cu-based cell-2. Reproduced from ref 64. Copyright 2023 Springer Nature.

CO_2_–CO cofeed valorization in a tandem CO_2_ electrolyzer system has been comprehensively studied by Möller and co-workers.^64^ Unlike tandem catalyst designs, tandem electrolyzers design allow independent optimization of catalytically active sites and electrolysis conditions in each step. In their system, a CO_2_-to-CO electrolyzer served as the first cell, enabling precise control over the CO_2_/CO composition of the outlet gas introduced into the second cell. This control was achieved by adjusting two key parameters: the applied current density and the CO_2_ feed rate. To assess the influence of CO in the feed, the authors conducted comparative experiments using a single Cu-based CO_2_ electrolyzer while varying both the current density and the CO molar fraction (Figure 8c–f). Ethylene production increased steadily with higher CO content, although the rate of increase slowed beyond 50% CO. In contrast, ethanol and n-propanol production rates exhibited more pronounced enhancements, especially at CO concentrations approaching pure CO input, indicating that oxygenates and C_3_ products are more sensitive to CO-rich conditions. The improved formation of C_2_ and C_3_ products was attributed not only to the elevated CO availability but also to the suppression of common C_1_ side products such as CO and formate, along with more favorable reaction kinetics and improved surface coverage of adsorbed hydrogen and CO intermediates. In addition, Weidner et al.^116^ investigated the influence of CO concentration in the gas feed on the production rates of C_2+_ products. When the CO concentration fed into the second electrolyzer decreased from ∼10.2 to 3.2%, the production rate of ethylene decreased by 15.6%, while that of ethanol increased by 35.2%, indicating a trade-off between the two products. This result does not contradict the findings of Möller et al.; rather, the Weidner study included only two data points, both at relatively low CO concentrations (10.2 and 3.2%). These findings from different studies further highlight the complex mechanisms underlying CO_2_/CO cofeed eCO_2_RR and underscore the emerging need for systematic investigation of tandem electrolyzer systems.

Beyond product formation rates, the CO_2_/CO feed ratio also plays a critical role in determining carbon selectivity. Tim Möller and co-workers^64^ reported that ethylene selectivity peaked at a CO fraction of approximately 33%, indicating that moderate CO levels are optimal for C_2_ hydrocarbon formation. In contrast, higher CO concentrations significantly enhanced the carbon selectivity toward oxygenates such as ethanol and propanol. Notably, the formation of n-propanol was found to be particularly sensitive to current density. At higher current densities, the selectivity for propanol decreased, likely due to reduced surface coverage of CO intermediates, which are essential for facilitating C_3_ product formation. This conclusion is supported by earlier experiments conducted under pure CO feeds at varying partial pressures, where limited CO availability was shown to suppress the formation of C_3_ oxygenates.^120–124^ These results underscore the importance of jointly optimizing both feed composition and operating current to balance product distribution and selectivity in tandem electrolyzers eCO_2_RR system.

While this study provides a well-structured investigation into CO_2_/CO cofeed strategies, several areas remain open for further exploration. Notably, all comparative experiments were conducted at a fixed total gas flow rate of 50 sccm, leaving uncertainty about how varying the total flow rate, either higher or lower, might affect product distribution, surface kinetics, or gas utilization. Additionally, when analyzing the unexpected single maximum pattern in hydrogen production, the authors attributed the trend to the declining total carbon content in the electrolyte with increasing CO fraction, citing the much lower solubility of CO compared to CO_2_. However, given that the experiments were carried out in a GDE, this explanation may underestimate the dominant role of the gas–solid–liquid triple-phase interface, which governs surface coverage more directly than bulk solubility. In GDE-based systems, local concentration and interfacial dynamics often outweigh solubility limitations. Finally, the tandem operation in their system achieved a maximum CO concentration of only 35% at the outlet of the first cell, indicating considerable room for improvement in both product formation rates and overall eEE.

#### 3.3.2 Gas Impurities Impacts

In practical tandem electrolyzer systems, feed gas purity plays a critical role in determining long-term performance, product selectivity, and system stability. While many studies focus on idealized pure gas feeds, real-world gas streams, particularly those derived from industry or from capture processes, often contain impurities such as residual CO_2_, ambient air, or trace contaminants. These impurities can interfere with key electrochemical pathways, degrade catalytic activity, or induce irreversible changes at the electrode–electrolyte interface. Understanding the effects of such impurities is essential for developing robust and scalable tandem systems suitable for industrial deployment. This section highlights recent findings on how both intentional and unintentional gas components influence performance outcomes in tandem electrolyzers setups.

**CO_2_** is the primary feed gas for the first cell in the CO_2_-to-CO conversion and may also be intentionally used as a cofeed component alongside CO in the second cell. However, in the second cell, CO_2_ is often not desired and can even be detrimental, particularly for acetate production, where CO_2_ has been shown to strongly inhibit formation. In this context, CO_2_ can be regarded as an impurity in the CO stream fed into the second cell, or as a contaminant within CO-rich feeds that adversely affect downstream reactor performance. Crandall et al.^50^ demonstrated that increasing the CO_2_ volume fraction in a CO_2_/CO mixture shifted the product distribution away from acetate and toward hydrogen, with hydrogen FE increasing from 10% to over 30% upon CO_2_ exposure (Figure 9a). Notably, after reverting to a pure CO feed, acetate production did not recover to its initial level, indicating irreversible performance losses. This deterioration is likely due to the formation of (bi)­carbonate salts at the cathode-electrolyte interface, which can impair catalytic activity and reduce the long-term stability of tandem systems. In their study, NaOH was used to absorb residual CO_2_ from the first cell, which introduces a notable recovery penalty for both CO_2_ and the alkaline absorbent. However, since the target product was acetate, known to be favored under highly alkaline microenvironments, this trade-off was considered acceptable.

**Figure 9:**
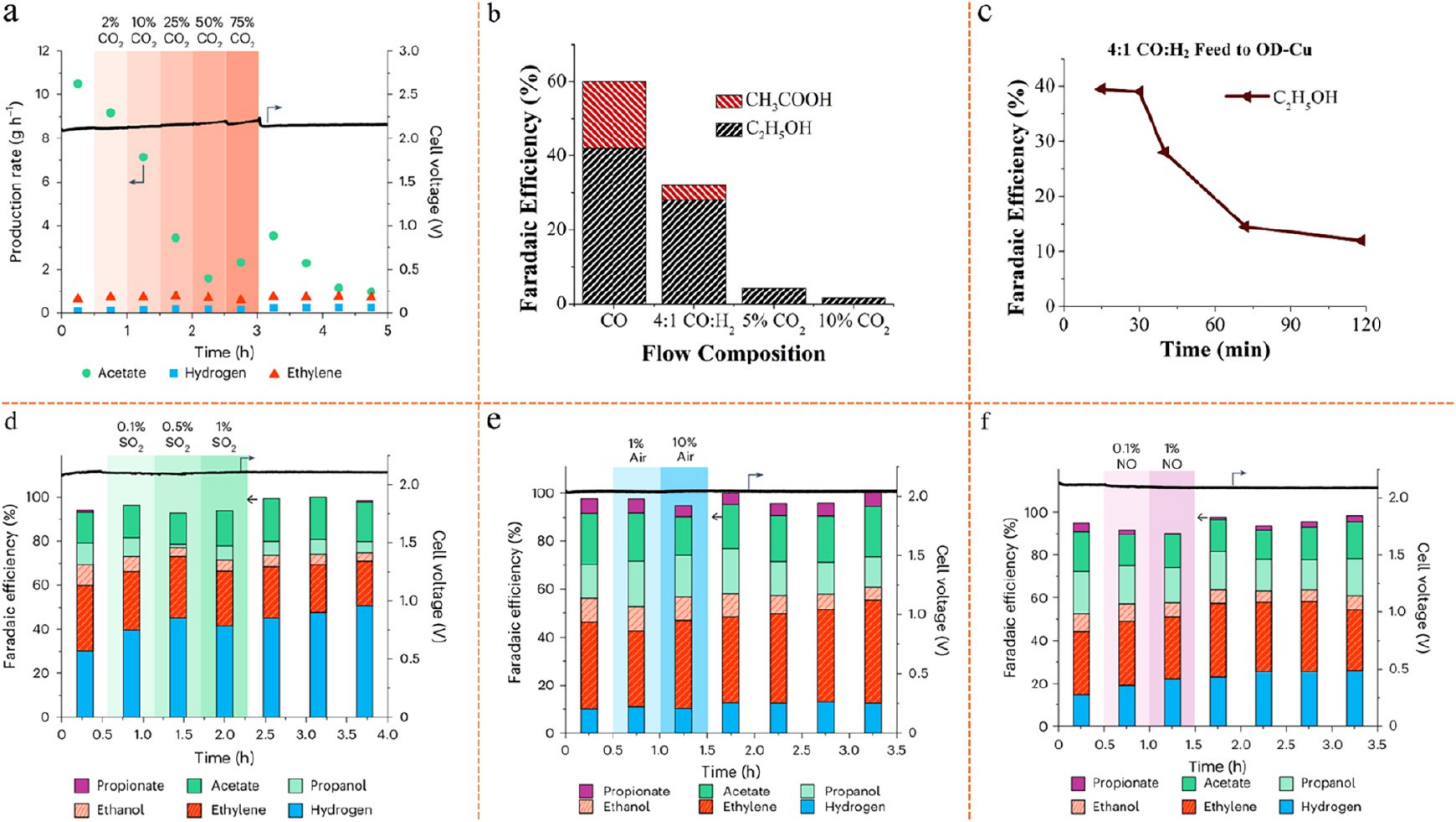
Effect of impurity content in the second step that eCORR in tandem electrolyzers eCO_2_RR. (a) Impact of unreacted CO_2_ on a 2-cell (200 cm^2^) CO electrolyzer stack at 100 mA/cm^2^. Reproduced from ref 50. Copyright 2024 Springer Nature. (b) Liquid product FEs for the second electrolyzer with varying flow composition, 5 and 10% CO_2_ compositions had a balance of 4:1 CO:H_2_. Reproduced from ref 113. Copyright 2018 Elsevier. (c) Second electrolyzer ethanol FE vs time with a 4:1 CO:H_2_ feedstock. Reproduced from ref 113. Copyright 2018 Elsevier. (d) Impact of air on a 2-cell (200 cm^2^) CO electrolyzer stack at 100 mA/cm^2^. Reproduced from ref 50. Copyright 2024 Springer Nature. (e) Impact of SO_2_ on a 2-cell (200 cm^2^) CO electrolyzer stack at 100 mA/cm^2^. Reproduced from ref 50. Copyright 2024 Springer Nature. (f) Impact of NO on a 2-cell (200 cm^2^) CO electrolyzer stack at 100 mA/cm^2^. Reproduced from ref 50. Copyright 2024 Springer Nature.

Early work by Theaker et al.^113^ investigated the impact of CO_2_ content and coexisting species on tandem system performance, revealing that even small amounts of CO_2_ in the gas feed can significantly reduce FEs for ethanol and acetate. In their study, a 4:1 CO:H_2_ gas mixture was used to mimic the output of the first cell, and incremental additions of CO_2_ (5 and 10% by volume) were introduced into the feed to the second cell, operated with an OD-Cu catalyst. The results showed a dramatic drop in ethanol FE from 28% (with no added CO_2_) to 5% at 5% CO_2_, and below 3% at 10% CO_2_, with acetate production disappearing entirely at higher CO_2_ concentrations (Figure 9b). These findings underscore the critical importance of minimizing or excluding CO_2_ entering the second cell to preserve high selectivity and efficiency for some desired multicarbon products in tandem electrolyzers eCO_2_RR.

**H**_**2**_ is an almost unavoidable byproduct in aqueous eCO_2_RR, and, unlike CO_2_, is challenging to remove from product gas streams. Its presence introduces competing reactions that can lower the FE for desirable products such as ethanol and acetate. Theaker et al.^113^ operated a tandem electrolyzers system where the gas stream from the outlet of first cell contained approximately 20% H_2_. For the second cell (with no ambiguous charge transfer affecting the calculation), the average FE for ethanol was 28.4%, significantly lower than the 42% obtained for the same second cell when operated with a pure CO feed (Figure 9b). To further confirm the H_2_ role, the authors simulated the second cell’s conditions by using a gas mixture CO/H_2_ with a controlled ratio of 4:1 (with no CO_2_) in individual tests. This setup also yielded an FE for ethanol of 28% after 40 min, matching the tandem result and showing a significant drop in acetate production compared to pure CO feed conditions (Figure 9c). These findings confirm that H_2_ behaves as a poisonous impurity in tandem eCO_2_RR systems, and its removal is much more challenging than CO_2_.

Given the high reducing power of H_2_ and its impact on catalyst performance, particularly for OD-Cu-based second cell catalysts, whose effectiveness for multicarbon product formation relies on maintaining an optimal oxidation state, excess H_2_ can disrupt this balance and may lead to continuous catalyst poisoning. This effect could explain the observed time-dependent decline in FE for ethanol, where cumulative C_2_H_5_OH FE decreases sharply beyond 30 min (Figure 9c). Therefore, maximizing CO selectivity and minimizing H_2_ formation in the first cell is highly desirable for sustaining efficient tandem operation. Fortunately, state-of-the-art CO_2_-to-CO electrolyzers can now approach this ideal. Notably, the authors also observed that, in the second electrolyzer, when operated under reductive potentials with the presence of CO/H_2_ mixtures, ethanol was further reduced to C_3+_ products. This suggests that the presence of a reductive agent like H_2_ may promote the formation of more complex hydrogenated and deeply reduced carbon products.

**Sulfur dioxide** (SO_2_), a common impurity in flue gas streams, was also examined for its influence on eCORR step^50^ (Figure 9d). SO_2_ is typically present in flue gas streams at concentrations of several hundred to a few thousand ppm, generally around 500–3000 ppm (0.05–0.3 vol %), even after desulfurization.^125,126^ Within this practical range (0.1–1 vol %), SO_2_ exposure was found to significantly shift product selectivity. Specifically, hydrogen FE increased from 30% to around 55% following SO_2_ exposure. XPS analysis indicated the possible formation of copper sulfide (Cu_2_S) on the catalyst surface, which may contribute to the enhanced hydrogen evolution. Despite this shift, the distribution of carbon products remained largely unchanged under CO electrolysis conditions, suggesting the adverse effects of SO_2_ were partially mitigated by the large electrode area, as evidenced by a slightly higher sulfur concentration on upstream cells. However, since SO_2_ can poison Cu catalysts even at very low concentrations, complete removal from the feed may not always be feasible. In such cases, incorporating a sacrificial upstream cell could serve as a practical mitigation strategy to intercept sulfur contaminants and protect the main electrolyzer stack in future scaled-up implementations.

**Air** impurities were also investigated by introducing 0–10 vol % ambient air into the CO feed^50^ (Figure 9e). The presence of air had limited effects on product distribution and cell voltage, with acetate, ethylene, and hydrogen (maintaining FEs below 15%) remaining the main products. The slight decline in efficiency was likely attributed to the oxygen reduction reaction, indicating that the tandem system exhibits a reasonable tolerance to low-level air contamination.

**Nitric oxide** (NO), the dominant component of NO_*x*_ in flue gas (typically accounting for 90 to 95%), was also examined for its influence on eCORR step^50^ (Figure 9f). Even under NO exposure, acetate and ethylene remained the primary products, and the cell voltage stayed stable. FE losses were modest, approximately 5% at 0.1% NO and 10% at 1% NO and fully reversible upon returning to a pure CO feed. Compared to CO_2_, the adverse effects of NO on CO electrolysis were less pronounced. XPS analysis indicated that nitrogen species were incorporated into the GDL as pyrrolic and pyridinic nitrogen, rather than binding to the copper catalyst surface. Elevated nitrogen concentrations on upstream cells further suggest that these cells act as passive barriers, absorbing most NO contaminants and helping to shield downstream electrodes. This finding supports the practical strategy of designing tandem stacks with upstream sacrificial cells to enhance impurity tolerance in scaled applications. Moreover, given the fully reversible nature of NO-induced performance losses, periodically feeding pure CO may serve as an effective operational strategy to restore activity and protect the integrity of the electrolyzer stack over time.

#### 3.3.3 Reaction Environment

The choice and design of the reaction environment, particularly the electrolyte, play a pivotal role in the performance, selectivity, and stability of tandem electrolyzers eCO_2_RR systems. In tandem architecture, where two or more electrochemical steps are integrated, electrolyte optimization is even more critical, as each cell may require different operating conditions to achieve optimal activity and product distribution.

For the first step, converting CO_2_ to CO, mild electrolytes such as potassium bicarbonate are most commonly used (see Table 1). These electrolytes enable high CO selectivity on catalysts like silver, gold, or single-atom Ni, while offering a good balance of ionic conductivity and reduced carbonate formation. Although higher alkalinity, such as 1 M KOH, can enhance reaction rates and boost SPCE, it also accelerates carbonate formation and exacerbates carbon loss, especially in flow cell systems. As a result, KOH is not used as the electrolyte for the first step in tandem designs; see Table 1 for a summary of typical electrolyte choices.

In the second step, typically the eCORR to C_2+_ products, the electrolyte is usually adjusted to a much higher concentration of strong alkaline solution, most commonly 1–5 M KOH. Thanks to the tandem design, the initial feedstock CO_2_ has already been largely removed, so the feedstock entering this stage is predominantly CO. This allows for the safe use of highly alkaline conditions without excessive carbonate formation. Under such environments, the increased alkalinity significantly enhances both the electrolyte conductivity and the formation of multicarbon products on copper-based catalysts, leading to improvements in both selectivity and production rate. A high pH not only suppresses the competing HER but also promotes C–C coupling, which is essential for producing key target products such as ethylene, ethanol, and n-propanol. Thus, the combination of elevated alkalinity and conductivity is a critical factor for optimizing the efficiency and performance of this second step.

For novel tandem electrolyzers eCO_2_RR systems targeting products such as high-purity C_2_H_4_, ethylene carbonate, and succinic acid, the choice of electrolyte is often dictated by proof-of-concept priorities rather than practical or scale-up considerations. In these studies, there is a tendency to use excessive quality/amounts of electrolytes, such as high concentrations of KOH in the first-step ethylene production, as seen in several recent publications. While such conditions are precisely what tandem eCO_2_RR strategies aim to address and improve upon, in these cases, the focus remains on demonstrating feasibility, maximizing selectivity, and achieving high production rates. Considerations like carbon loss, downstream process integration, or TEA are often secondary or not addressed. As a result, electrolyte selection in these novel tandem systems is driven more by the need to facilitate the desired reaction in each step than by the careful trade-offs for practical usage.

Furthermore, the trend toward zero-gap MEA designs and SOECs adds additional electrolyte engineering challenges and opportunities. In MEAs, the absence of a flowing catholyte minimizes carbonate formation and carbon loss but puts greater demands on membrane hydration and ionic conductivity. In SOECs, the solid-state ceramic electrolyte allows for pure CO generation without liquid-phase carbon loss but requires precise control of feed humidity and operating temperature.

Overall, the rational design and synchronization of electrolytes across tandem steps are essential not only for maximizing conversion efficiency and product selectivity but also for achieving long-term operational stability and minimizing energy consumption and carbon loss. Electrolyte engineering, therefore, stands as a key enabler for the practical deployment and scale-up of tandem electrolyzers eCO_2_RR technologies.

## 4 Product Distribution and Selectivity

Tandem electrolyzers eCO_2_RR systems are specifically designed to overcome the limitations of conventional single-cell configurations by enhancing the formation of multicarbon products through stepwise reaction pathways. In these systems, CO_2_ is first reduced to CO in the initial stage, which serves as a reactive intermediate for subsequent carbon–carbon coupling reactions in the second stage. The most reported products from tandem electrolyzer setups include C_2_ compounds such as ethylene, ethanol and acetate along with C_3_ products such as propanol. Among these, C_2_ products have received the most attention due to their high industrial relevance and relatively higher FEs achieved. Although the formation of C_3_ and higher hydrocarbons remains a significant challenge, emerging tandem strategies integrating advanced catalyst design and reaction engineering show promising potential to extend carbon chain growth beyond C_2_ species. Beyond the typical products generated from eCO_2_RR, tandem strategies open new possibilities for synthesizing more complex compounds, such as ethylene carbonate and succinic acid, offering sustainable alternatives to conventional energy-intensive petrochemical or biobased production routes. Advancing efficient tandem systems that can produce a broader spectrum of multicarbon products would mark a significant step toward the commercial viability of eCO_2_RR technologies, supporting more sustainable industrial practices and contributing to a low-carbon future.

### 4.1 C_2_ Products

#### 4.1.1 Ethylene

Ethylene is a key platform chemical in the petrochemical industry, serving as a fundamental building block for a wide range of downstream products, with global production capacity reaching approximately 228.53 million metric tons in 2023.^127^ Presently, the most widely adopted and commercial ethylene method is the steam cracking of ethane and naphtha, which is remarkably energy and GHG emission-intensive.^127,128^ As a result, the production of ethylene has attracted significant attention due to its substantial market value and the growing environmental concerns associated with its conventional fossil-based synthesis.

Möller et al.^64^ developed a low-temperature, neutral-pH tandem electrolyzer system to explore CO_2_/CO cofeed valorization (Figure 10a). The first cell selectively converted CO_2_ to a mixed CO_2_/CO stream over a precious-metal-free Ni–N–C catalyst, while the second cell utilized a Cu-based cathode to further reduce the feed into C_2+_ products, primarily ethylene and alcohols. The system achieved around a 50% increase in ethylene production rate at 500 mA/cm^2^ and a 100% improvement in alcohol formation at 700 mA/cm^2^ compared to single-cell setup. On a broader range of current densities up to 700 mA/cm^2^, the tandem setup also demonstrated an enhancement value up to 100% of overall C_2+_ eEE. This tandem architecture eliminates the need for a CO_2_ scrubbing step; however, experiments reveal that CO_2_ is selectively removed from the gas mixture by cathodically generated OH^–^ near the cathode, which effectively mimics the performance of CO-rich feeds, contributing to enhanced C–C coupling and improved selectivity toward C_2+_ products. The findings indicate that tandem electrolyzers can deliver kinetic and practical energy benefits compared with single-cell configurations when producing C_2+_ products directly from CO_2_ feeds, eliminating the need for intermediate CO_2_ removal. Nevertheless, this approach sidesteps the core objective of tandem electrolyzer designs, which is to tackle the carbonate formation challenge, rather than simply omitting the removal of intermediate CO_2_.

**Figure 10:**
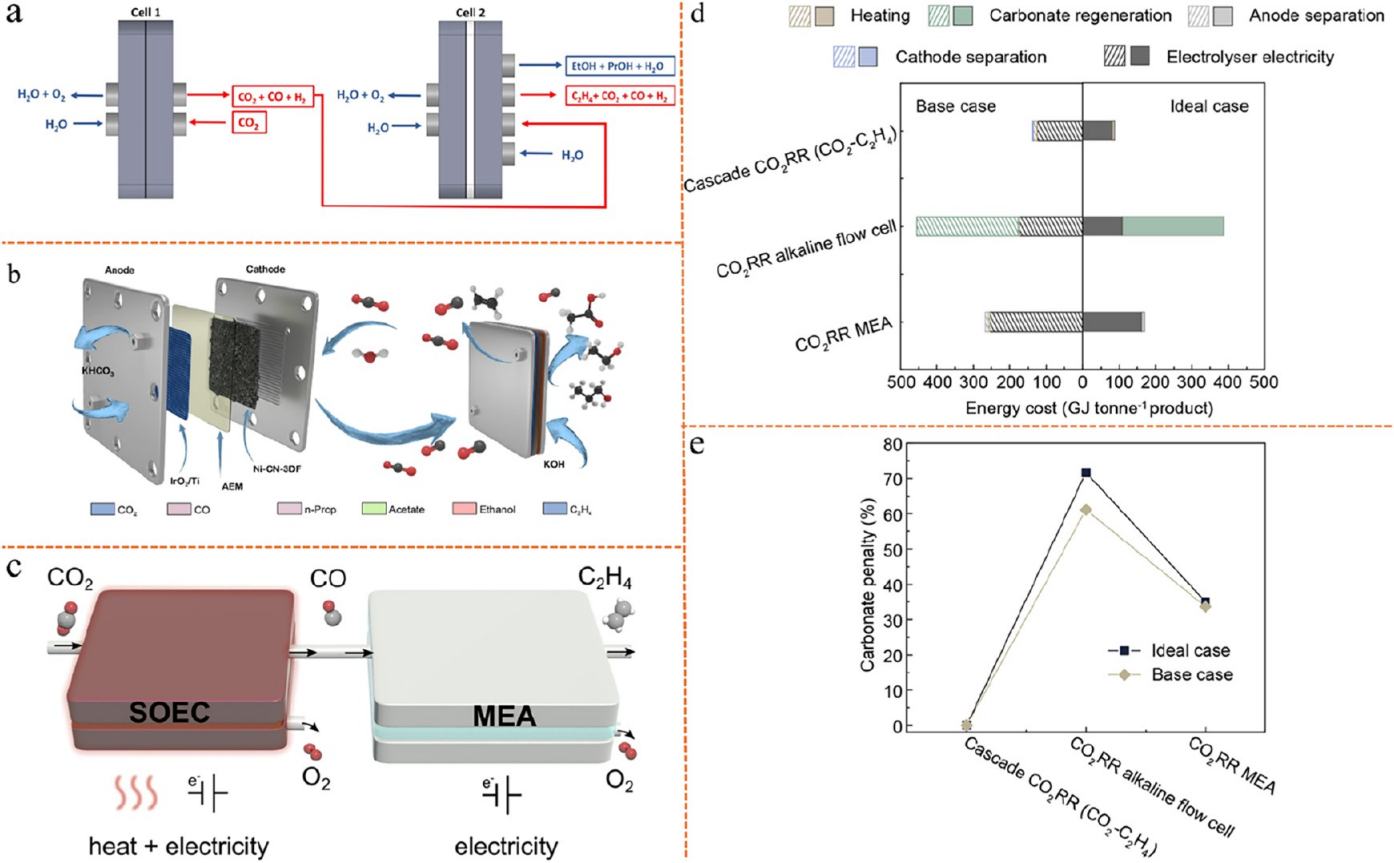
Schematic illustration of the tandem electrolyzers systems for CO_2_–CO–C_2_H_4_ and TEA of carbonate formation-free CO_2_-to-C_2_H_4_ production through tandem electrolyzers eCO_2_RR. (a) Concept of coupled tandem CO_2_ electrolyzers highlighting the flows of gas (red) and liquid (blue) reactants and products. The tail gas from cell 1 directly enters cell 2. Reproduced from ref 64. Copyright 2023 Springer Nature. (b) Schematic presentation of tandem electrolyzers. Reproduced from ref 101. Copyright 2024 Springer Nature. (c) Schematic illustration of renewable CO_2_-synthesized C_2_H_4_ in a combined system consisting of a CO_2_-to-CO SOEC and a CO-to-C_2_H_4_ MEA. Reproduced from ref 99. Copyright 2021 Elsevier. (d) Comparison of energy consumption for C_2_H_4_ production in various systems. Reproduced from ref 99. Copyright 2021 Elsevier. (e) The carbonate penalty (i.e., the fraction of energy consumption due to carbonate formation) in the various systems. Reproduced from ref 99. Copyright 2021 Elsevier.

In the study by Wu et al.,^101^ a tandem electrolyzers system was constructed with a first MEA cell using a Ni single-atom catalyst embedded in a porous 3D framework to achieve efficient CO production, and a second MEA cell employing a reported Cu_3_N-HDD catalyst optimized for multicarbon formation, see Figure 10b. The first cell generates a CO stream with 74.8% purity at a CO production rate of 34.4 mL·min^–1^. This abundant CO feed is subsequently delivered to the second cell, achieving a multicarbon FE of 93.5% at a current density of 800 mA/cm^2^. For comparison, recent work has shown that tandem catalyst systems, with similar catalyst selections but as a one-pot CuO/Ni single atoms tandem catalyst, only achieving an overall C_2+_ FE of 81.4%.^119^ Suggesting by inferior performance, a significant challenge for the above-mentioned single-pot cascade reactions lies in integrating multiple catalysts under a single set of optimized reaction conditions. However, the tandem design overcomes this limit by enabling independent optimization of each elementary reaction step, thereby maximizing both reaction kinetics and selectivity and showing a better FE of C_2+_. Through optimized control of the high concentration CO supply from the first cell and the maintenance of a strongly alkaline reaction environment in the second cell, the system establishes ideal conditions for C–C coupling, which is essential for efficient ethylene production. This highlights the superiority of the tandem approach in achieving precise reaction control and higher overall efficiency. The system achieved a remarkable single-pass FE of over 93% for total C_2+_ products at high current density (800 mA/cm^2^), demonstrating the effectiveness of the tandem approach for selective multicarbon synthesis. However, it is worth noting a methodological concern regarding the use of FE as a metric in the second cell when employing a CO_2_/CO cofeed. In this context, the FE plot presented in the article does not appear to rigorously account for the complexities introduced by mixed reactant feeds, which may complicate the interpretation of product selectivity and system efficiency.

ECO_2_RR has created promising pathways to low-carbon ethylene, but a significant challenge persists, substantial loss of CO_2_ to carbonate formation, which imposes severe energy penalties for electrolyte regeneration, about ∼278 GJ per ton C_2_H_4_ produced, accounting for a 60–70% of the total energy requirement^99^ (Figure 10d,e). Even in tandem systems discussed throughout this review, while carbonate formation is reduced compared to single-cell eCO_2_RR, it is not completely eliminated. Though MEAs result in less carbonate formation, CO_2_ inevitably reacts with cathodically locally generated OH^–^, resulting in some carbonate loss leads to 60–90 GJ of additional energy consumption per ton C_2_H_4_, accounting for an energy penalty of ∼35%^99^ (Figure 10d,e). Ozden^99^ and colleagues tackled this issue by employing the tandem electrolyzer strategies, first step CO_2_-to-CO is achieved in SOEC, followed by CO-to-C_2_H_4_ in a MEA (Figure 10c). Typically, individual experiments were performed for both steps in the tandem process. For the first step, the study identified that an optimal current density to CO_2_ flow rate ratio of 815 to 15 (mA/cm^2^: sccm) provided the best combination of CO selectivity and CO_2_ SPCE, with ∼91% FE of CO and a SPCE of ∼45%. In the second step, a Cu:Py:SSC catalyst is employed in an MEA with 3 M KOH, operating at 150 mA/cm^2^. Under these conditions, the system maintains a stable ethylene FE of 61% and a full-cell potential of 2.73 V for 110 h without any performance degradation. As an integrated system, the tandem system achieved a FE for ethylene of 58.7% and a total C_2+_ product efficiency of 76% at 120 mA/cm^2^, with stable operation for over 40 h and no detectable CO_2_ lost to carbonate.

This tandem approach leverages the liquid-electrolyte-free SOEC to eliminate carbonate formation and the benefits of a highly alkaline environment in the second step MEA. As a result, the total energy requirement for ethylene production was approximately 138 GJ/ton without energy penalty from carbonate formation, leading to a 48% reduction in energy intensity compared to the conventional single-cell eCO_2_RR process, which typically requires around 267 GJ/ton (Figure 10d,e). This work further improved the system by substituting the conventional oxygen evolution reaction (OER) at the anode with the glucose electrooxidation reaction (GOR), which possesses a thermodynamic potential approximately 1 V lower than that of OER (Figure 11a).^129^ This decrease in voltage lowered the total energy demand to 89 GJ/ton of ethylene, which is about 35% less than the 138 GJ/ton required by pairing OER at the anode at the same current density. This additional result with GOR demonstrates the potential for even greater reductions in the energy intensity of ethylene production. Overall, the work highlights a core advantage of the tandem electrolyzer strategies: each step can be independently optimized and controlled to maximize system efficiency and product yield. However, the high operating temperature of the SOEC (800 °C) raises concerns about harsh reaction conditions and stability. Overall, the tandem SOEC-MEA enables continuous ethylene production with record-low energy consumption, further improved by replacing OER with GOR at the anode. These results underscore the potential of tandem electrolyzer strategies to overcome key limitations of single-cell eCO_2_RR, advancing scalable and sustainable ethylene manufacturing from CO_2_.

**Figure 11:**
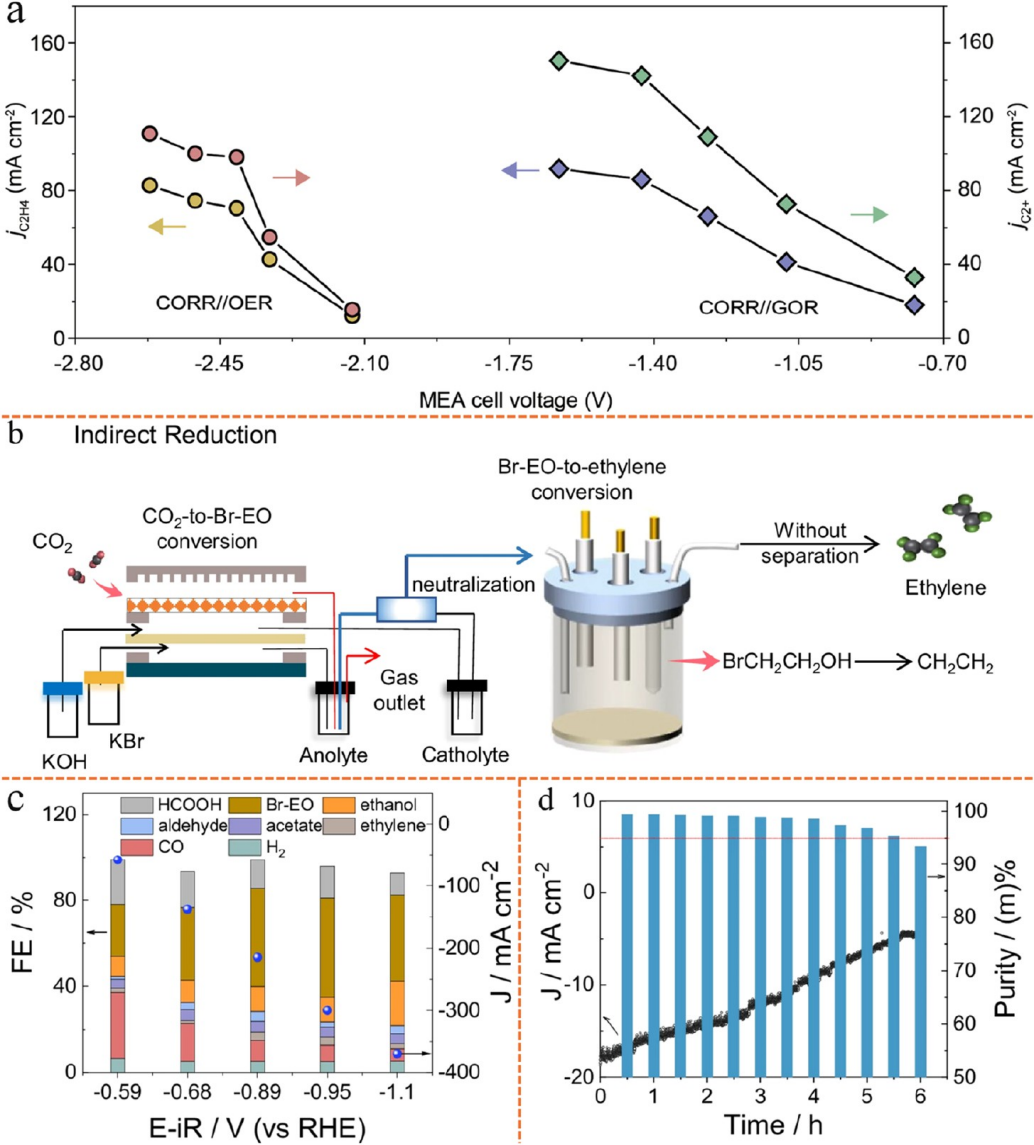
GOR replace OER as the anode half reaction in eCORR and high-purity C_2_H_4_ production in the tandem electrolyzers eCO_2_RR. (a) Effect of anodic reaction on the eCORR performance metrics of the MEA in the tandem eCO_2_RR. Reproduced from ref 99. Copyright 2021 Elsevier. (b) Schematic illustration of the tandem electrolyzers system of indirect reduction of CO_2_ for high-purity ethylene production. Reproduced from ref 84. Copyright 2024 Springer Nature. (c) FE and *J*_total_ of CO_2_ reduction products at different potentials. Reproduced from ref 84. Copyright 2024 Springer Nature. (d) *J* and the corresponding purity of ethylene collected by electrolysis in the neutralized anolyte of CO_2_ reduction electrolytic cell. Reproduced from ref 84. Copyright 2024 Springer Nature.

Li et al.^115^ developed a tandem electrolyzers eCO_2_RR system by coupling an acidic CO_2_-to-CO electrolyzer with an alkaline eCORR electrolyzer. In the first step, the two-electron CO_2_-to-CO conversion was carried out in a tailored acidic microenvironment (0.5 M K_2_SO_4_ + H_2_SO_4_ anolyte, pH 0.5, under 0.5 MPa CO_2_), achieving an eEE of 45% and reducing carbon loss by 86% compared with alkaline eCO_2_RR at 300 mA/cm^2^. High CO selectivity was obtained, with 95% FE at a partial current density of 475 mA/cm^2^ and an SPCE of 85% at 600 mA/cm^2^. The authors attributed the suppression of HER to high K^+^ concentrations, which decreased local H^+^ levels near the catalyst surface. In the tandem configuration, the first electrolyzer operated at 200 mA/cm^2^ using a Ni–N–C catalyst in a 25 cm^2^ MEA. The outlet gas passed through a CO_2_ trap for purification before being fed into the second electrolyzer, which employed a commercial Cu nanoparticle catalyst under alkaline conditions. At 1 A/cm^2^ in a 4 cm^2^ MEA, the second cell achieved FEs of 46% for C_2_H_4_ and 99% for total C_2+_ products, the highest reported to date. This two-step tandem strategy effectively divides the complex CO_2_-to-C_2+_ conversion into two more efficient reactions, each individually optimized. The acidic first step facilitates selective CO formation, while the alkaline second step promotes C–C coupling, collectively addressing both carbonation formation in alkaline electrolytes and the coupling limitation in milder conditions. However, no stability data were reported, likely due to the inherent instability of the acidic eCO_2_RR system.

In addition to carbonate formation in alkaline or neutral eCO_2_RR, a major challenge inherent to eCO_2_RR is the generation of a complex gas mixture containing unreacted CO_2_, H_2_, CO, CH_4_, and other byproducts. This necessitates energy-intensive separation processes to achieve the high-purity ethylene required for industrial polymerization.^130,131^ Ni and colleagues have developed an innovative indirect approach to address these challenges, allowing high-purity ethylene to be produced without the necessity of separation or purification steps.^84^ In this tandem strategy, CO_2_ is first electrochemically reduced to 2-BrEtOH in a bromide-containing electrolyte. The resulting 2-BrEtOH intermediate is then selectively reduced to ethylene using a Cl^–^ incorporated, electrochemically activated Ag/C catalyst (Figure 11b). Notably, 2-BrEtOH is readily generated by the reaction between CO_2_-derived ethylene and bromine produced at the anode.^132–134^ The eCO_2_RR performance of the Cu/Cu_2_O catalyst was tested in a flow cell with 1 M KOH serving as the catholyte and 1 M KBr as the anolyte. The gas exiting the cathode was passed through the anolyte container, where any remaining ethylene in the stream was converted into 2-BrEtOH. After 2 h of continuous CO_2_ reduction over the Cu/Cu_2_O catalyst at −0.95 V vs RHE, the resulting electrolyte exhibited a 2-BrEtOH selectivity of 46.1% (Figure 11c). This electrolyte was subsequently employed for 6 h of continuous electrolysis using an AC-Ag/C electrode at −0.48 V vs RHE, yielding an average ethylene purity of 98%, a level of selectivity and efficiency that has not been realized with single-cell eCO_2_RR approaches (Figure 11d). For this tandem electrolyzers route, the total electrical work is estimated at 310.99 GJ/ton of ethylene at a current density of 100 mA/cm^2^, which is much lower than the 564.3 GJ/ton required for thesingle-cell eCO_2_RR route using an alkaline flow cell.^130^ This strategy highlights how tandem electrolyzers processes that leverage specific intermediates can address persistent selectivity and purity challenges in conventional single-cell eCO_2_RR, opening new opportunities for scalable, low-carbon ethylene manufacturing with improved process efficiency and product quality. Altogether, this work provides new insights into the design of advanced tandem electrolyzers eCO_2_RR technologies for high-purity, energy-efficient ethylene production.

#### 4.1.2 Ethanol

With a global output of around 90 Mt/year, ethanol ranks among the most significant organic commodity chemicals.^135^ It is extensively used as a fuel blend component and serves as a vital precursor for synthesizing a variety of chemical compounds in the medical and food industries.^136^ At present, ethanol is primarily produced from starch-based biomass, such as sugar cane and corn, through fermentation. By eCO_2_RR, ethanol can be produced using intermittent renewable electricity and serve as an efficient, dense, and transportable energy carrier, suitable for both stationary energy storage and mobile energy applications. Its liquid phase at ambient conditions eliminates the need for high-pressure storage systems required by gaseous fuels, facilitating integration with current distribution networks. Moreover, converting renewable electricity into ethanol not only supports intermittent energy storage but also enables the valorization of CO_2_ into a high-value, carbon-neutral fuel for transportation, bridging the gap between renewable power generation and conventional fuel usage.

A pioneering and precursory work on ethanol production through tandem electrolyzers eCO_2_RR was conducted by Theaker et al.,^113^ who engineered two steps using spatially separated Ag and Cu catalysts. This tandem approach allowed each catalyst to operate under its optimal conditions, resulting in an overall FE of 11.0% for ethanol at an average applied potential of −0.52 V vs RHE, which was considered competitive at the time. The study also pointed out that Cu is less suitable for CO_2_-to-CO conversion due to its suboptimal binding energy, suggesting that a tandem approach with a dedicated and optimized CO production stage could outperform single-catalyst systems by reducing overall overpotential. This tandem design effectively mitigated catalyst interference and poisoning, while underscoring the critical role of controlled CO intermediate management in promoting C–C coupling and improving product selectivity toward liquid fuels.

Weidner et al.^116^ significantly increased the overall yield of C_2+_ products by enhancing the ethanol production rate by 28% at a current density of 300 mA/cm^2^, while maintaining the ethylene production rate in tandem electrolyzers eCO_2_RR. In the first electrolyzer, a Ni–Cu dual-atom catalyst was employed in a flow cell to produce a CO_2_ gas stream containing approximately 10.2% CO at a current density of 400 mA/cm^2^, which was then directly fed into the second electrolyzer. In the second electrolyzer, a newly developed surface Al-rich Cu/CuO_*x*_ catalyst was utilized in a flow cell, resulting in a 28% increase in the ethanol production rate at 300 mA/cm^2^ compared with single-electrolyzer measurement. Considering the unreliability of FE in tandem electrolyzers experiments, all results were reported in terms of production rate, which provides a clearer and more accurate basis for comparison and analysis. By analyzing the CO production rates from both the single-electrolyzer and the tandem electrolyzer measurements, the combined CO production rate from the single cells was found to be 10.3% higher than that measured in the tandem configuration. This clearly demonstrates that CO formed in the first electrolyzer is more efficiently converted into C_2+_ products in the second cell than CO generated in situ within the second cell itself. These findings strongly support the advantages of tandem electrolyzer strategies in unlocking new reaction pathways through CO/CO_2_ mixed feed and in enhancing the overall production of C_2+_ products.

Wang et al.^117^ achieved a high FE of 90.9% for liquid C_2_ products in the second step of CO-to-C_2+_ conversion in tandem electrolyzers for eCO_2_RR. In the first step, Ag NCs@Ag-MOF catalysts with highly dispersed Ag nanoclusters (NCs) were synthesized via a prereduction strategy, where Ag-NH_2_BDC MOF was treated at −0.68 V (vs RHE) for 30 min. These nanoclusters (∼6 nm), generated through Ag^+^ reduction and subsequent migration-aggregation under applied potential, served as the primary active sites for CO_2_-to-CO conversion. In the second step, Cu–O_2_N_2_–COF nanoparticles (∼50 nm) were synthesized, with atomically dispersed Cu species (valence state close to +2) anchored on the COF framework. This unique structure was attributed to the remarkably high FE toward liquid C_2_ products, in contrast to the conventional understanding that Cu^+^ favors C–C coupling while single-atom Cu is generally unfavorable for C_2+_ formation. For tandem electrolyzer operation, the first cell operated at −0.78 V (vs RHE) with a FE for CO of 99.1%; unreacted CO_2_ was removed by NaOH solution placed at the gas outlet. The second cell achieved 90.9% FE for liquid C_2_ products (ethanol and acetate) at −0.98 V (vs RHE) with a partial current density of 120 mA/cm^2^, maintaining stability for at least 5 h. This two-electrolyzer tandem system allows free selection and combination of catalysts, overcoming potential mismatching and maximizing the catalytic efficiency of each catalyst, thereby enabling high selectivity toward C_2+_ products.

#### 4.1.3 Acetate

Acetic acid or acetate is a vital feedstock in numerous industrial applications as a current industrial bulk chemical product and one of the most important organic acids.^137,138^ Widely known as the primary component of vinegar, acetic acid holds significant industrial value, with global production exceeding 16.7 million metric tons annually. Valued at 20.6 billion USD in recent years, the market is projected to reach USD 31.9 billion by 2030, reflecting its growing demand across multiple sectors.^139^ Acetic acid is used as a solvent in terephthalic acid production and, in the food industry, serves both as a preservative and a flavoring agent, with growing interest in its potential for antimicrobial coatings and packaging. Its salts, such as sodium acetate and potassium acetate, are also utilized as food additives.^140^ In addition, downstream products like acetic anhydride and vinyl acetate are key bulk chemicals in organic synthesis, plastics, pharmaceuticals, and agriculture.^138^ Cellulose acetate is widely applied in the manufacture of photographic films and textiles, while volatile acetic esters function as solvents in paints, coatings, and adhesives.

Its central role in metabolic pathways and its diverse range of downstream applications underscore the strategic importance of acetic acid in both traditional manufacturing and emerging biobased technologies.^141^ In biosynthesis, acetate’s value has been further elevated by demonstrating its conversion into complex, high-value chemicals. For example, engineered *Escherichia coli* can metabolize acetate into succinate,^142,143^ while other bioconversion pathways yield products like itaconic acid,^144^ glucose, fatty acids,^145^ acetyl-CoA, and 3-hydroxypropionic acid.^146,147^

At present, industrial-scale acetic acid and acetate production involves multiple steps: syngas is thermocatalytically converted to methanol at 5–10 MPa and 250 °C, after which methanol undergoes methyl carbonylation with CO to yield acetic acid. This conventional route is energy-intensive, relies on fossil fuel sources, and produces approximately 1.6 kg of cradle-to-gate CO_2_-equivalent emissions for every kg of acetic acid generated, depends on costly rhodium catalysts, and experiences corrosion problems from iodide additives, all of which add to both the environmental impact and operational expenses.^138,140^

Acetate is one of the most promising eCO_2_RR products since it requires only four electron transfers per CO_2_, while other common multicarbon products require at least six electron transfers per CO_2_. Acetic acid has the highest market value (0.68 USD/kg) of the most common CO_2_ electrolysis products, enabling large profit margins.^50^ In contrast to traditional thermal processes, acetate and acetic acid can be produced from CO_2_ or CO and water as reactants through eCO_2_RR or eCORR, powered exclusively by electricity.^148,149^ They present a more sustainable alternative, operating under mild conditions and enabling intermittent, decentralized production, an advantage that becomes increasingly relevant as renewable electricity prices continue to fall.^150–152^ While Cu-based catalysts have demonstrated strong performance in generating C_2+_ products such as ethylene^153^ and ethanol,^154^ the single-cell eCO_2_RR to acetate remains comparatively underexplored. Acetate generally appears only as a minor byproduct and exhibits lower selectivity than ethylene and ethanol in eCO_2_RR.^155,156^ Recent studies indicate that although ethylene and ethanol share similar reaction pathways,^157–159^ the formation of acetate proceeds through a distinctly different mechanism with limited mechanistic understanding,^160,161^ which may partly account for its lower yield in typical eCO_2_RR systems. Given the inherent challenges in simultaneously achieving high selectivity and activity for acetate production through single-cell eCO_2_RR, an attractive alternative is to decouple the process into two sequential steps: first converting CO_2_ to CO, followed by selective eCORR to acetate.

For acetate production, the nucleophilic attack of OH^–^ on key C_2_ intermediates, such as *CCO or *CH_2_–CO, plays a crucial role in directing the reaction pathway toward acetate formation.^162,163^ The notable advantage of the tandem electrolyzers approach is that it allows the use of a highly alkaline environment rich with OH^–^ in the CO electrolyzer, which uniquely enhances selectivity for acetate.^160^ Starting from CO also simplifies the overall reaction, as two fewer electrons per molecule are required to produce acetate compared to starting from CO_2_, while also leveraging recent improvements in eCORR. Recent studies further demonstrate that MEA-based CO electrolyzers can generate concentrated and pure acetate streams, achieved by integrating an alkaline stable and ethanol-permeable AEM with a selective ethanol partial oxidation anode catalyst.^94^

Crandall et al.^50^ have demonstrated significant progress toward scalable acetate production via tandem electrolyzers eCO_2_RR. A kilowatt-scale tandem electrolyzer system, consisting of a 500 cm^2^ CO_2_ electrolyzer and a 1000 cm^2^ CO electrolyzer, successfully produced acetate at industrially relevant rates. Specifically, the system achieved stable operation at 300 A over 125 h, yielding 98 L of acetate solution (1.2 M concentration) with 96% purity. Initially, the researchers developed individual CO_2_ and CO electrolyzers, both employing 5 cm^2^ MEAs, to achieve high reaction rates with low resistance. In the CO_2_ electrolyzer, metallic Ag particles supported on a carbon-based GDL served as the cathode, paired with an IrO_2_-based anode. This system achieved optimal CO_2_ conversion (∼47%), producing a CO-dominant gas stream containing less than 10 vol % residual CO_2_ at 400 mA/cm^2^ and maintaining approximately 80% FE toward CO. In the CO electrolyzer, a Cu cathode and a NiFeO_*x*_ anode operated at current densities up to 500 mA/cm^2^ (∼2.3 V), primarily yielding acetate and ethylene with hydrogen FE maintained below 10%. Upon integrating the optimized individual cells, a NaOH-based CO_2_ trap was employed to remove residual CO_2_, a step considered necessary here since CO_2_ acts as an impurity in acetate production. This difference likely arises from distinct reaction pathways involved in acetate formation compared to those for other C_2+_ products. To achieve a highly pure acetate stream, the anolyte in the CO electrolyzer was recirculated across the NiFeO_*x*_ anode, enhancing partial oxidation of alcohols to carboxylates. The integrated watt-scale system produced approximately 20% FE ethylene and 50% FE acetate, maintaining stable performance over 200 h with the liquid product stream containing ∼99% acetate purity (Figure 12a). Stability was evidenced by minimal degradation in cell potential (∼1 mV/h) and maintained hydrogen FE below 30%. However, performance declined significantly at around 300 h, indicated by an increase in hydrogen FE to approximately 50%. This deterioration was attributed mainly to Fe contamination of the Cu cathode in the CO electrolyzer. Thus, while the first electrolyzer demonstrated sufficient maturity for integration, using an inexpensive metal–nitrogen–carbon (MNC) catalyst could further reduce costs. The primary remaining challenge is improving the stability of the Cu cathode in the second cell, highlighting the need for developing more durable Cu-based catalysts and robust anode materials. The study highlighted the benefits of the tandem approach, notably overcoming common challenges such as unwanted carbonate formation and limited selectivity inherent to conventional single-cell eCO_2_RR.

**Figure 12:**
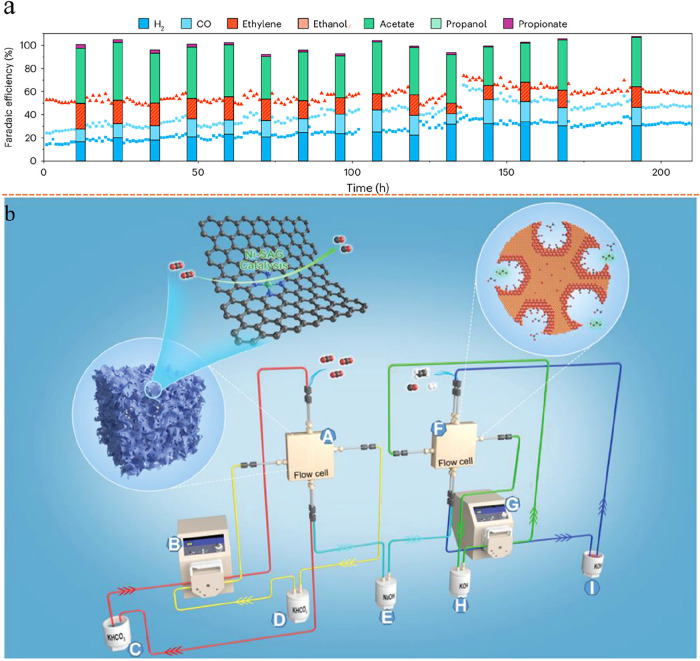
Watt-scale integrated CO_2_ and CO electrolyzer performance for acetate production and scheme of two-step eCO_2_RR for CO_2_ to n-propanol. (a) FE versus time for integrated two-step CO_2_ electroreduction. Reproduced from ref 50. Copyright 2024 Springer Nature. (b) Scheme of two-step CO_2_ electrochemical reduction for CO_2_ to n-propanol. Reproduced from ref 114. Copyright 2022 John Wiley and Sons.

### 4.2 C_3_ Products

#### 4.2.1 Propanol

Alcohols are considered an attractive target in eCO_2_RR because they combine high energy density with convenient liquid-phase storage and transport, making them promising candidates for practical fuel applications.^135,164^ Among C_1_–C_3_ alcohols, n-propanol exhibits one of the highest volumetric energy densities (27 MJ·L^–1^) and a high-octane rating, providing strong resistance to engine knock. Although its gravimetric energy density (33 MJ·kg^–1^) is lower than that of gasoline (45 MJ·kg^–1^), these combustion properties make it a promising candidate for blending with gasoline or serving as an alternative fuel.^165^ In the chemical industry, n-propanol is an important solvent and a precursor in various chemical and pharmaceutical processes. Its global annual production is about 0.3 Mt, and it is projected to grow at 6.6% per year through 2030.^166^ N-propanol can also be dehydrated under mild conditions to yield propylene, valued at approximately USD 130 billion annually, which serves as a major feedstock for polypropylene, propylene oxide (used in polyurethane), and acrylonitrile.^167^ However, the current industrial supply of propylene comes almost entirely from oil- and gas-based processes, which are fossil-fuel-dependent and highly energy-intensive. At present, n-propanol is manufactured through the hydroformylation of ethylene with CO and H_2_ at about 20 MPa and 80–150 °C to form propanal, which is then hydrogenated with H_2_.^168^ The combined feedstock use and process emissions result in more than 4 tonnes of CO_2_-equivalent being released for every tonne of n-propanol produced.^169^ In contrast, isopropanol is produced on a much larger scale, several million tonnes annually and widely used as a solvent, disinfectant, and chemical intermediate but is less often considered for fuel applications. Both routes are currently based on fossil-derived feedstocks, though they operate under different reaction conditions and serve largely distinct market demands.

The electrosynthesis of propanol using only CO_2_ and water as inputs, powered entirely by renewable energy, has recently attracted considerable interest as a sustainable alternative to fossil-based production. However, single-cell eCO_2_RR to n-propanol is hampered by limited eEE and significant CO_2_ losses, primarily due to the carbonate formation-related energy penalty. Furthermore, the direct conversion of CO_2_ to propanol faces significant kinetic barriers, as it requires an overall transfer of 18 electrons and two C–C coupling steps. Achieving this in a single electrolyzer is challenging, as it demands exceptionally well-designed catalytic sites: some must drive the reduction of CO_2_ to CO intermediates, while others must facilitate the subsequent C–C bond formation. Such limitations often cause low activity and poor selectivity for propanol, thereby reducing the potential of single-cell eCO_2_RR for its production. With the increasing maturity and availability of electrochemical CO_2_-to-CO conversion technologies, eCORR has become a promising pathway for synthesizing propanol. Compared to eCO_2_RR, the eCORR route requires fewer electrons (12 vs 18) and benefits from strong alkaline conditions that favor multicarbon product formation, enabling good performance in generating C_3_ products. Moreover, recent research indicates that eCORR intrinsically favors the production of alcohols, particularly propanol.^168,170–173^ However, practical considerations regarding the cost, safety, and complexity of storing and handling gaseous CO limit its industrial application. Consequently, the tandem electrolyzer strategies have gained significant attention. In this tandem process, CO_2_ is first reduced to CO, which is then electrochemically converted to propanol, offering a promising strategy to overcome the limitations of the single-cell eCO_2_RR approach.

Wu^114^ and colleagues introduced a two-step tandem electrolyzers approach, efficiently converting CO_2_ first into CO using a 3D single-atom Ni catalyst and subsequently transforming this CO to n-propanol employing a reported multihollow Cu_2_O (Figure 12b). Typically, each step in the tandem process is individually optimized before integration. For the initial CO_2_-to-CO conversion, the researchers selected a cost-effective, high-performance, and easily scalable nickel single-atom catalyst (Ni SAC), avoiding the use of precious metals like silver and thereby reducing capital costs. Following optimization, the CO FE reached a maximum of 99.2% at 140 mA/cm^2^. For the second-step eCORR to propanol, multihollow Cu_2_O nanoparticles with surface cavities, considered favorable for propanol formation, were selected as catalysts. C_2+_ products dominate across a wide current density range of 20 to 160 mA/cm^2^, with n-propanol reaching a maximum FE of 30.2% at 42.5 mA/cm^2^ with a partial current density of 12.8 mA/cm^2^. In the final tandem system, the residual CO_2_ in the gas stream exiting the first cell is removed using a 5 M NaOH purifier. However, the reduction of the gas flow rate from 30 to 3 sccm before entering the second cell indicates that the CO_2_-to-CO conversion efficiency is still quite low. After purification, the CO-rich gas feeds into the second cell, which consistently yields n-propanol with a FE of 28.0% at 42.5 mA/cm^2^, slightly lower than the 30.2% achieved with pure CO. Other major products include ethylene (29.8%), ethanol (25.7%), and acetic acid (7.2%). The observed performance gap between individual and integrated tandem experiments underscores the importance of evaluating the combined system rather than relying solely on pure CO-fed eCORR results. Although this work does not resolve the carbonate formation issue and still exhibits substantial carbon loss, it nevertheless demonstrates the use of tandem electrolyzers in eCO_2_RR for the selective production of n-propanol. Importantly, such tandem systems show particular promise for generating higher-carbon and more complex products, such as n-propanol, that are difficult to obtain through single-cell eCO_2_RR alone.

### 4.3 Niche Products

#### 4.3.1 Succinic Acid

Succinic acid is recognized as a key biobased platform molecule, listed by the U.S. Department of Energy among the top ten value-added chemicals from renewable feedstocks. It has wide-ranging applications in pharmaceuticals, surfactants, additives, and polymer manufacturing. It also serves as a precursor to high-value derivatives such as 1,4-butanediol (BDO), tetrahydrofuran (THF), and γ-butyrolactone (GBL). In contrast to conventional petrochemical or fermentation-based routes, producing succinic acid via eCO_2_RR offers a cleaner and more sustainable pathway, thereby enhancing the environmental performance of chemical manufacturing.

The sustainable production of multicarbon chemicals directly from CO_2_ remains a major challenge in electrocatalysis, particularly for long-chain hydrocarbons like succinic acid. Mao et al.^87^ reported an innovative tandem system that couples CO_2_ electroreduction with electrocarboxylation, achieving efficient upcycling of CO_2_ as the only carbon feedstock into succinic acid under mild conditions (Figure 13a). In this two-step process, CO_2_ is first electrochemically converted to ethylene using a CuO-based catalyst in a flow cell. The resulting ethylene gas is then directly fed, without purification, into a second electrocarboxylation cell where it reacts with additional CO_2_ over a FeNi foam cathode, yielding succinic acid (Figure 13b). As a result, 0.12 μmol of succinic acid was obtained in 5 h of cascading catalysis. This cascade approach addresses key bottlenecks of previous methods, notably by eliminating the need for complex gas separations and demonstrating the feasibility of direct CO_2_ conversion to longer-chain products, such as C_4_ product succinic acid, which cannot be obtained through a single-cell eCO_2_RR but is accessible via tandem electrolyzers design. Importantly, this process results in a negative cradle-to-gate carbon footprint of −0.174 kg CO_2_/kg succinic acid, outperforming both petrochemical (1.94 kg CO_2_/kg SA) and biobased (0.88 kg CO_2_/kg SA) production routes. This work demonstrates that tandem electrochemical systems can provide scalable and economically viable pathways to high-value C_3+_ chemicals, opening promising prospects for sustainable, carbon-negative manufacturing from CO_2_.

**Figure 13:**
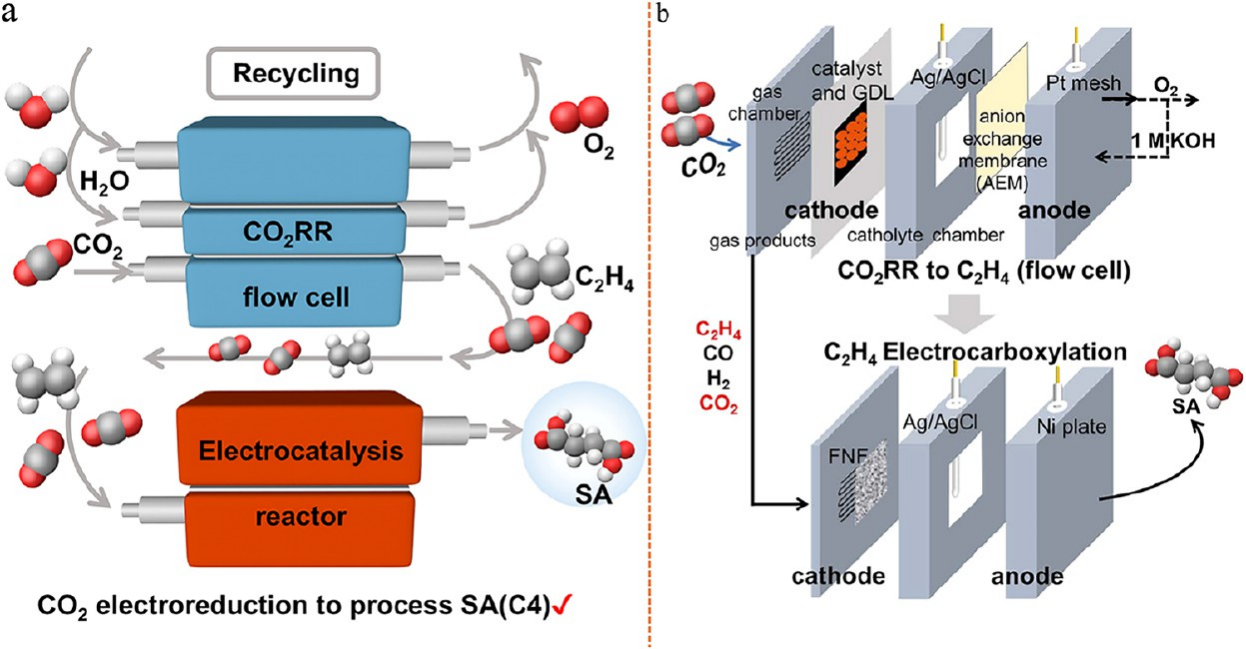
Schematic illustration of the tandem electrolyzers system for succinic acid production. Reproduced from ref 174. Copyright 2025 Elsevier. (a) Schematic illustration of tandem catalysis to produce succinic acid from CO_2_. (b) Schematic illustration of renewable CO_2_-synthesized succinic acid in a combined system consisting of a CO_2_-to-C_2_H_4_ flow cell, and a C_2_H_4_-to-succinic acid single cell.

#### 4.3.2 Ethylene Carbonate

Ethylene carbonate stands out as a valuable building block for the chemical industry, thanks to its remarkable versatility. Its strong polarity, chemical stability, high boiling point, and low toxicity make ethylene carbonate an outstanding organic solvent, offering a safer alternative to traditional polar aprotic solvents like acetonitrile, dimethylformamide, and *N*-methyl-2-pyrrolidone.^175^ Beyond its role as a solvent, ethylene carbonate serves as an essential intermediate in the production of polymers, is used in gas separation processes, and finds applications in surface coatings, among many others.^176,177^ Perhaps most significantly, ethylene carbonate is a crucial ingredient in lithium-ion battery electrolytes. As global efforts intensify toward electrification and the electric vehicle sector continues to boom, the demand for ethylene carbonate is set to soar, with market projections estimating growth from 0.8 billion dollars in 2024 to 1.5 billion by 2030.^178^ However, current industrial production of ethylene carbonate primarily relies on a thermocatalytic process in which epoxides, such as ethylene oxide, react with CO_2_ at elevated temperatures and high pressures (Figure 14a).^86,179^ Notably, the ethylene oxide feedstock required for ethylene carbonate production is currently derived from fossil resources through an energy-intensive, silver-catalyzed aerobic epoxidation of ethylene, thereby further tying ethylene carbonate production to conventional petrochemical supply chains (Figure 15a).^86,180–182^ The development of sustainable strategies for ethylene carbonate production under ambient conditions is highly attractive but currently unavailable.

**Figure 14:**
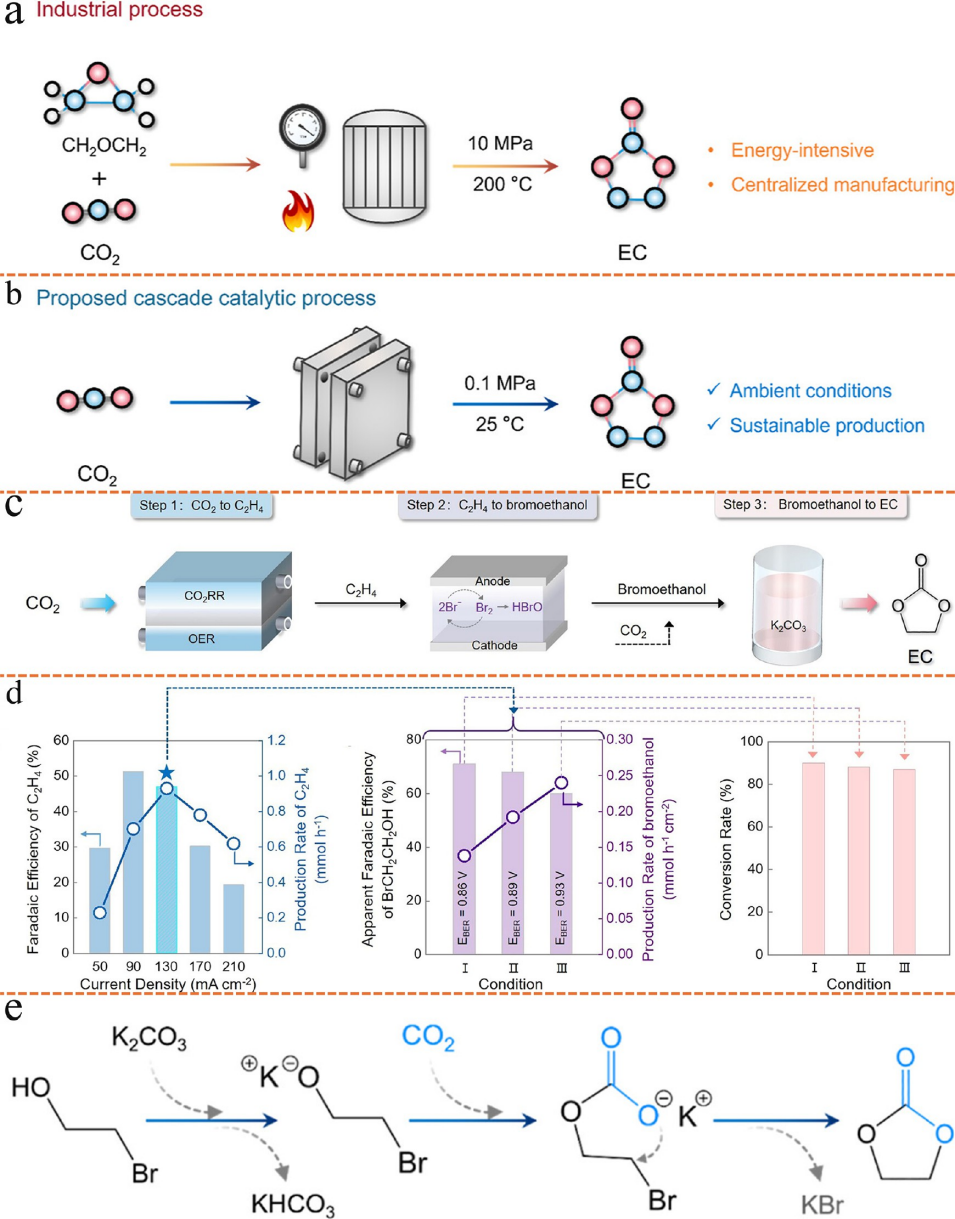
Schematic comparison between the industrial process and the proposed EC-EC-C tandem method for ethylene carbonate production, tandem conversion of CO_2_ to ethylene carbonate, and the key step of conversion of 2-BrEtOH and CO_2_ to ethylene carbonate. Reproduced from ref 85. Copyright 2024 American Chemical Society. (a) Industrial process for ethylene carbonate production. (b) Proposed tandem catalytic process for ethylene carbonate production. (c) Schematic showing the three-step catalysis. (d) Corresponding three-step catalysis. From left to right; Left: FE and production rate of the CO_2_RR to C_2_H_4_ under different current densities. Middle: Apparent FE and production rate of the bromine-mediated conversion of ethylene to 2-BrEtOH at different working potentials with the gas feed from the CO_2_RR at 130 mA/cm^2^ (a total current of 650 mA). Right: Conversion rate of 2-BrEtOH to ethylene carbonate using the 2-BrEtOH aqueous solution prepared in last step, and (e) the corresponding proposed reaction pathway.

**Figure 15:**
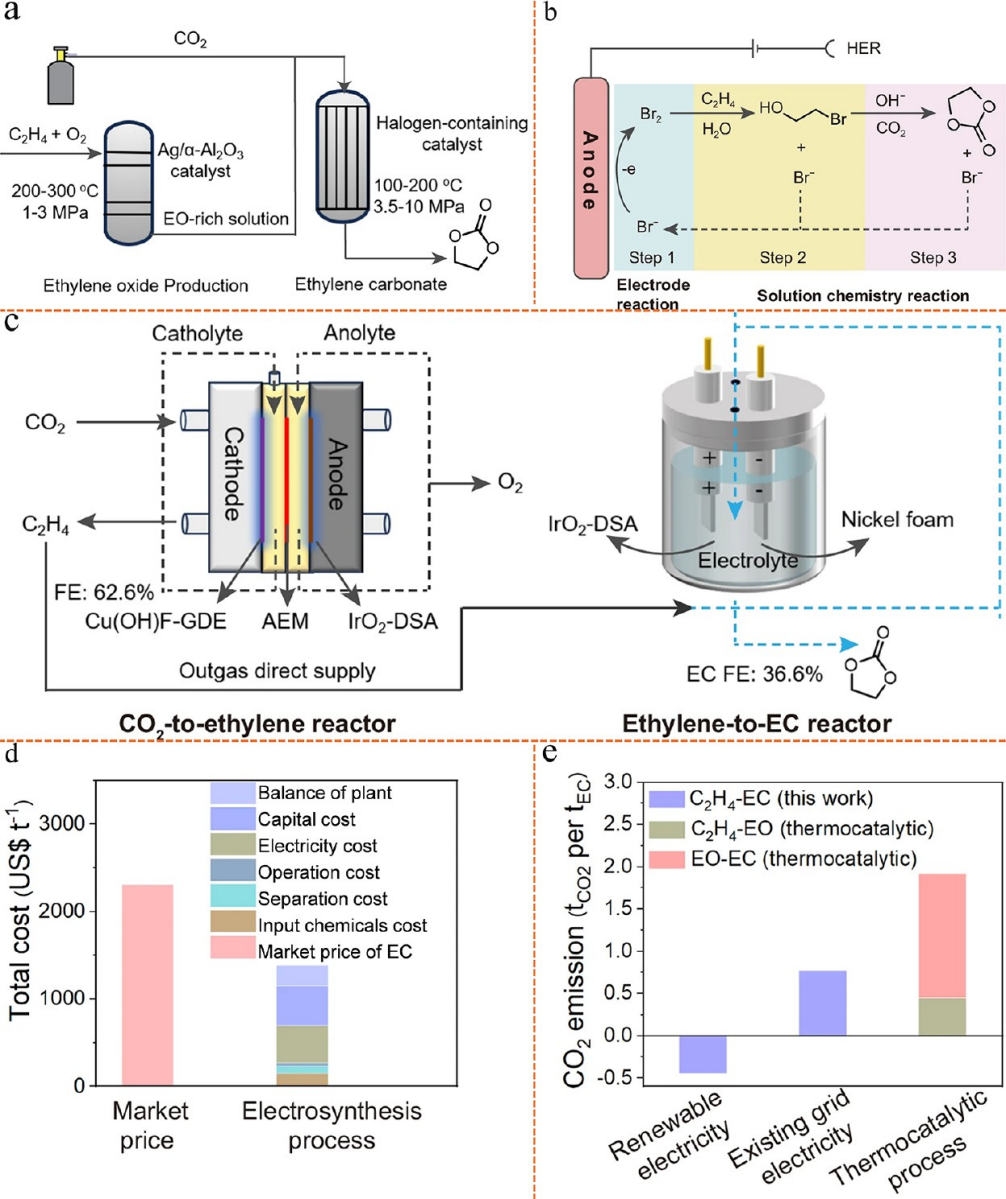
Routes for ethylene carbonate production and TEA and CO_2_ emission of ethylene carbonate electrosynthesis, and tandem electrolyzers CO_2_-to-ethylene carbonate synthesis process. Reproduced from ref 86. Copyright 2025 Springer Nature. (a) Existing industrial thermocatalytic route for ethylene carbonate production. (b) The proposed bromide-mediated membraneless direct ethylene carbonate electrosynthesis strategy using ethylene and CO_2_ as the feedstocks. (c) Schematics of the two-stage tandem CO_2_-to-ethylene carbonate system. (d) TEA of single-step ethylene-to-ethylene carbonate electrosynthesis. (e) The comparison of the CO_2_ emission for ethylene carbonate synthesis from ethylene using different production methods.

Thus, Wen^85^ and colleagues demonstrated a tandem catalytic route for converting CO_2_ directly into ethylene carbonate under ambient temperature and pressure, which represents the first demonstration of the successful synthesis of organic carbonates from CO_2_ as the exclusive carbon source (Figure 14b). Their process couples three steps (Electrochemical-Electrochemical-Chemical): eCO_2_RR to ethylene, bromine-mediated conversion of ethylene to 2-BrEtOH, and subsequent reaction of 2-BrEtOH with CO_2_ to yield ethylene carbonate (Figure 14c).

For the initial CO_2_-to-ethylene step, production rate is prioritized over selectivity to meet the demands of downstream reactors, resulting in the selection of a current density of 130 mA/cm^2^ and a corresponding ethylene production rate of 0.93 mmol/h (Figure 14d). The MEA reactor’s outlet gas is directly bubbled into the anode compartment of an H-cell for the subsequent bromine-mediated conversion. For the second step, C_2_H_4_-to-2-BrEtOH, newly developed WO_3_ nanoarrays supported on carbon cloth were selected, achieving an average FE for 2-BrEtOH of 89%. However, when integrated into the cascade, the bromine-mediated FE was somewhat reduced (71% at 0.86 V, 68% at 0.89 V, and 60% at 0.93 V) compared to individually optimized operation (consistently above 80%), highlighting the importance of real-world tandem devices implementation. In the final step, the conversion of 2-BrEtOH to ethylene carbonate was achieved with an optimized conversion rate of about 90%. Figure 14e illustrates a proposed reaction pathway, in which it is believed that K_2_CO_3_ first abstracts a proton from the −OH group of 2-BrEtOH to generate an alkoxide nucleophile. This intermediate then reacts with CO_2_ and subsequently undergoes intermolecular ring closure to form ethylene carbonate.^183,184^ The presence of a bromine atom in 2-BrEtOH increases the electrophilicity of the adjacent carbon, thereby enhancing its susceptibility to nucleophilic attack by CO_2_ and significantly lowering the activation energy of the reaction. This work underscores the potential of tandem electrolyzers eCO_2_RR, which combines electrochemical and chemical transformations, to broaden the possibilities for CO_2_ valorization and to enable sustainable, decentralized production of complex carbonates like ethylene carbonate under mild operating conditions.

Cai et al.^86^ introduce a sustainable, bromide-mediated membraneless electrosynthesis that enables the direct conversion of ethylene and CO_2_ into ethylene carbonate under ambient conditions, with FE ranging from 47 to 78% at industrially relevant current densities. This strategy achieves high ethylene carbonate concentrations (up to 0.86 M) and demonstrates excellent operational stability for over 500 h. To realize these results at scale, the authors adopt an efficient one-pot design that optimizes reaction kinetics via electrolyte engineering. In the ethylene carbonate electrosynthesis in the second cell, bromine or hypobromite generated at the anode reacts with dissolved ethylene through a halohydrin pathway to produce 2-BrEtOH, which subsequently cyclizes with CO_2_ to form ethylene carbonate while regenerating bromide ions (Figure 15b). Importantly, the authors integrated ethylene carbonate electrosynthesis with upstream eCO_2_RR to ethylene, thereby establishing a full tandem process that employs only CO_2_ and water as feedstocks (Figure 15c). In this tandem system, the eCO_2_RR to ethylene step used sheet-shaped copper fluoride hydroxide (Cu­(OH)­F) as the catalyst, achieving a C_2_H_4_ FE of ∼63% at 100 mA/cm^2^ on a 5 cm^2^ electrode, where the gas was directly fed into the second cell. In the second cell, a FE of 37% was achieved at 6 mA/cm^2^. The lower FE for ethylene carbonate than the individual second step is mainly attributed to the limited ethylene output from the small-area CO_2_ electrolyzer, as opposed to a pure and efficient ethylene feed, underscoring the need for further system optimization before practical implementation.

Techno-economic and carbon assessments were demonstrated for the stand-alone C_2_H_4_-to-ethylene carbonate step using fossil-derived ethylene. Overall, this ethylene carbonate electrosynthesis route shows superior performance compared to previously reported approaches in terms of stability, ethylene carbonate concentration, current density, and productivity. The TEA estimates the production cost at US $1379.9/ton (based on an electricity price of $0.10/kWh), which is notably lower than the U.S. market price of $2305/ton as of March 2024 (Figure 15d). Relative to conventional ethylene carbonate production pathways relying on fossil-fuel-derived ethylene oxide, this process also achieves lower CO_2_ emissions, even under the current average grid emission intensity (0.295 kg CO_2_/kWh, EU data). When powered entirely by renewable electricity, the system attains net-negative carbon emissions, consuming 0.45 tons of CO_2_ per ton of ethylene carbonate produced (Figure 15e). Moreover, with declining electricity prices and continued progress in CO_2_-to-C_2_H_4_ electrosynthesis, the projected production cost of the integrated tandem CO_2_-to-ethylene carbonate process could fall to ∼USD $1275/ton when electricity costs reach USD $0.04/kWh. Under these conditions, powering with renewable electricity (emission factor: 0.01 t/MWh) results in overall CO_2_ emissions of −1.39 ton_CO2_/ton_EC_, substantially lower than the −0.46 ton_CO2_/ton_EC_ achieved in the fossil-ethylene-based single-step route. These findings underscore the potential of tandem electrolyzer systems for scalable, low-carbon ethylene carbonate production directly from CO_2_, offering both environmental and industrial advantages.

## 5 Performance Metrics and Benchmarking

### 5.1 Metrics in Tandem Electrolyzers System

#### 5.1.1 Faradaic Efficiency

Faradaic efficiency (FE) represents the fraction of the total electric charge that contributes to forming a desired product in an eCO_2_RR. It reflects how effectively electrons are utilized for the desired chemical transformation, rather than being consumed by side reactions such as hydrogen evolution. FE is typically expressed as a percentage and serves as a key performance metric in evaluating the selectivity and electrochemical utilization of energy in CO_2_ reduction systems. However, in tandem electrolyzers system, particularly those without an intermediate CO_2_ scrubbing step, the application of FE becomes less meaningful and potentially misleading. In single-cell systems, FE is a reliable metric for selectivity, as there is only one cell structure, one inlet, and one outlet, making product detection and quantification relatively straightforward. By contrast, in tandem systems, the overall FE for target C_2_ and higher products is typically lower than that of a single cell, not because of inferior performance, but due to the system design. When designing a tandem electrolyzers system, the primary objective is to generate a high CO molar fraction from the first cell. This design inherently requires the first cell to consume a significant portion of the total electrons to convert CO_2_ into CO, which then serves as the intermediate gas feed for the second cell. However, as the CO concentration increases, a larger share of the total charge is allocated to CO production in the first cell, an electron usage that is unrelated to the selectivity or catalytic performance of the second cell. As a result, the calculated FE for C_2+_ products in the tandem configuration appears artificially low when both cells’ currents are summed in the denominator. From an academic standpoint, this disconnect renders FE inadequate for evaluating or comparing catalyst activity and mechanistic performance between tandem and single-cell systems. From an industrial or practical perspective, while single cell setups may show higher FE for target products, what ultimately matters are the overall eEE, which directly influences the cost of electricity energy input. Therefore, when comparing the performance of different tandem electrolyzer systems, the relevance of FE becomes even less clear. Since the current densities of the two cells can be independently optimized to maximize overall eEE, rather than to achieve a higher combined FE, using FE as a primary benchmark fails to capture the true performance potential of the system.

If FE cannot be meaningfully applied by summing the contributions of both cells, it is also problematic to apply it solely to the second cell in tandem systems. In practice, accurately quantifying FE in the second cell becomes challenging due to the complexity of the feed composition. When the input gas contains both CO and CO_2_ simultaneously, the specific reaction pathway leading to a given product, such as ethylene, is ambiguous. Ethylene formation may proceed through CO–CO coupling (involving 8 electrons), CO_2_–CO coupling (10 electrons), or CO_2_–CO_2_ coupling (12 electrons), making the exact electron transfer number difficult to assign. This undermines the reliability of calculated FEs for C_2_ and higher products in the second cell, as the true stoichiometry of the electrochemical process cannot be definitively established. Therefore, while FE remains a useful parameter within isolated systems, it should be interpreted with caution or complemented by other metrics when evaluating tandem setups.

Therefore, while FE remains a useful metric in single-cell systems, its limitations in tandem setups necessitate additional evaluative tools. In tandem electrolyzers systems, production rate and carbon selectivity together address the shortcomings of FE. Production rate provides a direct and practical measure of system output, enabling meaningful comparisons across different conditions, especially when FE calculations become unreliable, such as in CO_2_/CO cofeeding experiments. In parallel, carbon selectivity offers mechanistic insight by clarifying product distribution and shedding light on competing reaction pathways. Together, these complementary metrics establish a more comprehensive framework for assessing system performance and understanding fundamental processes in tandem electrolyzers eCO_2_RR, guiding both experimental optimization and mechanistic studies.

#### 5.1.2 Production Rate: Performance beyond FE

Production rate is a critical parameter for evaluating the practical performance of tandem electrolyzers eCO_2_RR, especially those without the intermediate step CO_2_ scrubbing, where the FE is not suitable for indication and comparison anymore. It represents the quantity of desired product generated per unit time, typically reported as mmol/h, g/h, or normalized to electrode area (e.g., mA/cm^2^ for partial current density). Unlike selectivity-focused metrics, production rate directly reflects the throughput and productivity of a system, which are essential considerations for industrial application and scale-up.

Production rate is an especially valuable metric for optimizing individual steps in tandem electrolyzers systems. For instance, in the first step, CO production, a high production rate of CO is crucial to ensure that sufficient reactant is supplied for downstream CO-to-C_2+_ conversion, regardless of whether a CO_2_ absorber is used. If the first cell cannot provide enough of the desired product to serve as feedstock for the second step, the performance of the latter will inevitably suffer due to reactant limitation. This means that when FE and production rate are not simultaneously maximized in the upstream reactor, which is often the case, optimization strategies tend to prioritize the highest possible production rate over FE.^85^ This is particularly relevant when CO_2_ is not participating in the second or subsequent cell reactions, as in tandem configurations with intermediate CO_2_ absorption. Therefore, a focus on production rate enables better integration and performance matching between consecutive steps in tandem systems, supporting more efficient overall operation.

The production rate serves as a practical and informative metric for evaluating electrochemical performance, especially when FE cannot be accurately determined. This situation commonly arises in CO_2_/CO cofeeding experiments, where complex and overlapping reaction pathways make precise FE calculations impossible for the second electrolyzer. Under these conditions, with a fixed applied current, variations in feed composition, reaction environment, or catalyst design can be effectively assessed by tracking the production rate of the target product. For instance, based on single-electrolyzer measurements for both steps, the production rates of the respective products in each individual electrolyzer can be measured and assumed constant throughout the entire measurement sequence. Since C_2+_ products are only generated in the second electrolyzer, any change in their production rates during tandem electrolyzer operation can be directly correlated with variations in the CO_2_/CO input ratio, electrolyte pH, or catalyst structure, allowing the production rate to serve as a meaningful and practical substitute for FE in comparative studies.

However, it is important to recognize the inherent limitation of production rate as a metric: it considers only the amount of product formed, without accounting for the consumption of reactants, input energy, or feedstock. This makes direct efficiency comparisons between different research groups or system designs challenging. Moreover, attempts to normalize production rate by current or current density, in an effort to make it more like an efficiency metric, ultimately render it similar to FE and diminish its unique value. Thus, while production rate is indispensable for comparative studies under certain experimental conditions, it should be used in conjunction with, rather than as a replacement for, efficiency-based metrics whenever possible.

A high production rate indicates the system’s ability to deliver sufficient quantities of valuable multicarbon products under continuous operation, which is vital for commercial viability. However, increasing production rates often bring challenges such as mass transport limitations, product crossover, and catalyst stability issues. In tandem systems, the production rate of each step must be balanced to ensure smooth integration, avoid bottlenecks, and maintain consistent intermediate supply (e.g., CO from the first cell for downstream conversion). Optimizing production rate therefore requires a holistic approach, considering not only the intrinsic activity of catalysts and reactor design, but also overall system integration and operational stability. Reporting and benchmarking production rates, alongside selectivity and eEE, provides a more comprehensive and practical assessment of tandem electrolyzers eCO_2_RR performance.

#### 5.1.3 Carbon Selectivity: Mechanistic Insight beyond FE

Carbon selectivity is a useful metric for evaluating the performance of eCO_2_RR systems, especially in mechanism analysis of tandem electrolyzer configurations targeting multicarbon products. Unlike FE, which measures the proportion of total current used to generate a particular product, carbon selectivity focuses on the distribution of carbon input among the different products formed during the reaction. It is typically defined as the fraction of carbon atoms from the CO_2_ (or CO) feedstock that are converted into a specific product or class of products, relative to the total carbon-containing products detected.

In tandem systems, carbon selectivity offers valuable insight into how efficiently the integrated process directs carbon toward the desired multicarbon products while minimizing the formation of less valuable C_1_ byproducts or gaseous losses. Beyond serving as an overall process metric, carbon selectivity is especially valuable for mechanistic studies in tandem electrolyzers system. This more granular perspective allows researchers to track how carbon is partitioned among different multicarbon products under varying operating conditions. For example, in CO_2_/CO cofeeding experiments, adjusting the feed ratio—such as by increasing the CO concentration—generally increases the production rate of most multicarbon products. However, a detailed analysis of carbon selectivity reveals divergent trends for different products, shedding light on their distinct formation mechanisms. In Möller’s study,^64^ for instance, a feed with 33% CO resulted in ethylene achieving the highest carbon selectivity, while further increases in CO concentration shifted carbon selectivity toward alcohols, suggesting that a higher proportion of CO feedstock favors alcohol formation over olefins. Additionally, increasing the applied current density tends to boost the overall production rate for all multicarbon products, but from the perspective of carbon selectivity, ethylene’s share remains relatively unchanged. In contrast, higher current densities significantly enhance the carbon selectivity for ethanol at the expense of propanol, reflecting mechanistic differences: lower surface coverage of CO at high currents disfavors C_3_ product formation. Thus, carbon selectivity not only provides a nuanced assessment of process efficiency but also offers mechanistic insights into product distribution and pathway competition within tandem electrolyzers eCO_2_RR.

Ultimately, carbon selectivity serves as a practical and relevant metric for comparing different system designs and operating strategies, providing a clearer understanding of overall carbon utilization efficiency in tandem electrolyzers eCO_2_RR. This focus on carbon allocation is especially critical for industrial applications, where both process economics and sustainability are directly impacted by how effectively input carbon is converted into value-added products.

#### 5.1.4 Single-Pass Conversion Efficiency

SPCE measures the portion of the input reactant, usually CO_2_ or CO, that is transformed into the target products during one pass through the electrolyzer, without recycling. In principle, a higher SPCE is always advantageous, as values closer to unity (one) mean more complete reactant utilization, lower downstream separation costs, and greater process efficiency. However, in real systems, achieving very high SPCE is limited by factors such as mass transport constraints, side reactions, and the need to balance conversion with selectivity.

There are often trade-offs between SPCE, FE, and other performance metrics. For example, the FE of eCO_2_RR to CO can approach unity, whereas the SPCE generally remains below 50%. Efforts to increase SPCE to very high values can adversely impact FE for the desired product, as evidenced by enhanced competing reactions such as HER and the onset of irreversible performance degradation. Likewise, optimizing for maximum FE may reduce SPCE if it requires operating at higher reactant concentrations or flow rates. These inherent trade-offs must be carefully managed depending on system design and target application. As a result, recent research focuses on optimizing SPCE alongside FE and production rate, recognizing that practical process performance depends on a balance among all these metrics. Overall, SPCE serves as an essential indicator of process intensification and scale-up potential for tandem electrolyzers systems, providing critical insights into electrolyzer design, catalyst performance, and the overall feasibility of CO_2_ electrochemical valorization at an industrial scale.

#### 5.1.5 Stability

Stability is a critical benchmark for advancing tandem electrolyzers in eCO_2_RR from laboratory studies to industrial applications. In real systems, the two cells often exhibit a mismatch in durability: simple intermediates such as CO, generated in the first cell, typically show much longer operational stability compared to the more complex multicarbon products formed in the second cell.^185^ This disparity is particularly evident when copper, the primary catalyst for C_2+_ products, suffers from well-known atomic-scale instability and dynamic surface reconstruction during extended electrolysis.^186,187^ Such instability compromises long-term selectivity and efficiency, directly affecting both operational costs and capital investments required for scale-up. In evaluating tandem electrolyzer strategies, the expected operational longevity should not simply mirror that of individual single-step systems. Instead, stability benchmarks must account for the shorter continuous operation times that are realistic when multiple cells are integrated in sequence.

To date, only a limited number of tandem electrolyzers demonstrations have achieved meaningful operational stability, with one study reporting over 200 h of continuous acetate production and another reaching 40 h for ethylene production.^50,99^ For the first step of CO_2_-to-CO, Hao et al.^111^ reported stable eCO_2_RR performance for up to 4500 h using an acid-humidified CO_2_ gas input. However, for the second step, stability is markedly lower. For example, targeting acetate, Zhu et al.^163^ demonstrated over 150 h of continuous operation for pure acetic acid generation via eCORR. In the study by Crandall,^38^ the individual second cell of eCORR with pure CO feed exhibited 125 h of operation at a total current of 300 A. This stability is lower than that reported for the tandem configuration in the same work, but the difference is primarily attributable to scale-up effects, rather than the 200 h achieved with small-area tandem MEAs. Using a direct CO_2_-to-acetate single-cell configuration, Zhu et al.^149^ constructed a tandem catalyst system that sustained electrolysis for 200 h. For ethylene, Bao et al.^52^ achieved a FE of 71% with stable activity during a 68 h eCORR run. In a direct CO_2_-to-ethylene single-cell approach, Fang et al.^51^ reported 72% selectivity for CO_2_-to-ethylene conversion at a current density of 800 mA/cm^2^, with durable stability of 230 h. She et al.^188^ further demonstrated pure-water-fed eCO_2_RR to ethylene with >1000 h of stability at 10 A, without CO_2_ or electrolyte losses, and with 50% FE. Beyond C_2_ products, Wang et al.^53^ achieved n-propanol with a FE of 36% ± 3% at 300 mA/cm^2^, maintaining stable operation for 100 h. The stability of tandem electrolyzer strategies remains unsatisfactory compared with single-cell eCO_2_RR. This is understandable, as the incorporation of additional electrolyzers increases system complexity, tandem methods are not primarily designed to address stability limitations, and potential bottlenecks (i.e., “short-board effects”) can arise. Moreover, this area has only recently come into focus, and further progress will require time and systematic development.

#### 5.1.6 Electrical Energy Efficiency

Electrical energy efficiency (eEE) is a critical metric in tandem electrolyzers eCO_2_RR, representing the proportion of supplied electrical energy that is successfully converted into the target chemical product. For a single electrochemical cell, eEE is typically calculated as the ratio of the theoretical energy content of the product, quantified by its Gibbs free energy change, to the total electrical energy consumed during the process. This metric provides direct insight into the practical utility of cell design and is essential for comparing different catalysts, electrolyzer configurations, and operational conditions.

In the context of tandem systems, by analyzing eEE at the single-cell level, researchers can identify inefficiencies and energy losses specific to each step. Such a stepwise approach enables rational design of tandem setups where each component operates close to its energetic optimum, ultimately improving the overall process efficiency. However, when evaluating the combined tandem system, eEE should, in principle, be one of the most important metrics for tandem design. Yet, its true significance is often underappreciated in current research. In practice, to ensure sufficient downstream conversion, the first cell is typically operated to generate a surplus of intermediate (such as CO), creating a concentrated feed that enables optimal performance in the second cell. This direct connection and real-time transfer of intermediates between cells, rather than separating the two steps, represents one of the biggest advantages and defines characteristics of tandem design, as highlighted previously in the fundamentals section of this review. While many studies report high eEE values for the individual cells, often presented separately for the first and second stages, these numbers do not always reflect the performance of the integrated system. When the two cells operate together in true tandem mode, the overall eEE for the desired product (such as ethylene) is typically lower than expected, since the intermediate (e.g., CO) is not the final target. This discrepancy underscores the need for system-level eEE analysis in tandem designs, emphasizing that metrics based only on individual cell performance can be misleading for evaluating the true process efficiency.

Ultimately, eEE is closely tied to the overall electricity cost of tandem electrolyzers systems. In a field where electricity represents the single largest operating expense, even small improvements in eEE can have a profound impact on process economics and scalability. As the price of renewable electricity continues to fall, maximizing system-level eEE becomes even more critical for ensuring the commercial viability of CO_2_ conversion technologies. Future research should therefore prioritize integrated energy analysis and cost optimization, making eEE not just a scientific metric but a practical guide for advancing sustainable and economically competitive CO_2_ utilization.

#### 5.1.7 Energy Intensity

Energy intensity serves as the final gate between laboratory research and industrial deployment, directly connecting technical progress in the field to real-world economic and sustainability outcomes. Unlike eEE, which focuses on the ratio of useful energy output to input within a single device, energy intensity quantifies the total energy consumed per unit mass of product, encompassing the entire process chain, including electrolysis, feedstock generation, product separation, and purification. This makes it a particularly relevant metric for TEA analysis and for evaluating the industrial scalability of tandem electrolyzers eCO_2_RR.

Recent studies demonstrate that, even with major improvements in catalyst design and reactor engineering, overall energy intensity remains one of the greatest challenges for tandem CO_2_-to-multicarbon conversion. For example, state-of-the-art tandem systems converting CO_2_ to ethylene currently achieve energy intensities of around 166 GJ per ton of product, an improvement over single-cell CO_2_-to-ethylene routes (224 GJ/ton) but still above the industrial target of 80–110 GJ/ton needed for cost-competitive, decarbonized manufacturing.^96^

Driving energy intensity lower will require coordinated advances at every step, from maximizing SPCE and minimizing intermediate losses, to deploying highly selective and robust catalysts and optimized cell and membrane designs. Both modeling and recent experiments indicate that with improved operational strategies and materials, the energy intensity for tandem electrolyzers eCO_2_RR could ultimately fall below 100 GJ per ton for major products like ethylene. In summary, energy intensity is a holistic benchmark that connects fundamental research to practical implementation, ensuring that advances in tandem electrolyzers eCO_2_RR are aligned with economic and environmental realities for industrial-scale applications.

### 5.2 Suggested Standardized Metrics

Given the stepwise nature of tandem electrolyzers design and their commercial relevance in overcoming carbon loss and carbonate formation, it is essential to establish standardized metrics that capture both the individual and integrated performance of each electrolyzer in the system. While all conventional metrics from single-cell strategies remain relevant, tandem configurations introduce additional parameters that provide deeper insight into system efficiency and optimization potential. For example, for the product C_2_H_4_, in the optimal model: the first cell MEA operates in a near-neutral environment with CO_2_ removal between the two steps, while the second cell MEA runs in a highly alkaline environment. The separation and recycling of gas mixtures in both steps are also considered, such as recycling CO_2_ in the first step and CO in the second step (The total SPCE is not mentioned below, since the preferred intermediate CO_2_ removal and possible recycling of unreacted/recovered CO_2_ and CO, the total SPCE for tandem systems is not necessarily required). Therefore, reporting the following suggested standardized metrics for tandem electrolyzer designs include:

1. The complete FE distribution for each cell, so researchers know which step can be improved.
2. The total FE for both cells, so researchers can determine how many electrons are wasted due to unmatched reaction rates between the two steps; for example, a surplus supply of CO from the first step will significantly lower the total FE.
3. The current density of each cell, especially when combined with FE to obtain the partial current density of CO and C_2_H_4_, which is useful for evaluating the reaction rate and potential for industrial application.
4. The total current of each cell, which generally indicates the scale-up potential; high current density with a small total current is less desirable than high current density with a larger total current, as the latter demonstrates a better transition from lab to practical use.
5. The cell voltage of each cell, where a lower cell voltage means less overpotential and better eEE.
6. The concentration of CO in the outlet of the first cell. Although it will likely go through a CO_2_ removal process before being fed to the second cell, a higher CO concentration means less CO_2_ needs to be removed, which can reduce separation costs. In addition, a higher CO concentration also means fewer cycles of CO_2_ through the first cell may be needed.
7. The SPCE in the first cell. While similar to the CO concentration in the outlet of the first cell, SPCE is generally lower due to carbonate formation and nearly unity FE for CO. If there is no carbon loss, SPCE should be close to the CO concentration. Higher SPCE results in fewer cycles for unreacted CO_2_.
8. Another important metric is the CO_2_ crossover rate. Although this rate is related to the reaction mechanism, with the OER at the anode, the mixture of crossover CO_2_ and O_2_ requires additional separation costs based on the CO_2_ crossover rate.
9. The concentration of C_2_H_4_ in the outlet of the second cell, which is relevant to separation, purification, and recirculation costs.
10. The SPCE for C_2_H_4_ in the second cell, similar to the concentration metric.
11. The eEE for each step, which is important for final TEA.
12. The energy intensity for each step and for the whole process, which is a fundamental advantage of tandem design compared to the single-cell strategy.

## 6 Challenges and Prospects

### 6.1 Carbon Loss

Carbon loss represents a major challenge in eCO_2_RR systems, fundamentally limiting both process efficiency and overall carbon utilization. In most low-temperature designs such as flow cells and MEAs, alkaline electrolytes are generally applied to mitigate competing HER and to promote C–C bond formation. However, a significant fraction of the CO_2_ feed is lost to carbonate formation, often exceeding the amount of CO_2_ converted to the target product, depending on factors such as electrolyzer types, reaction environment, and catalyst selection.^61,189–191^ Most notably, carbonate species are formed through reaction with hydroxide ions, whether present in the bulk catholyte or generated in situ at the cathode. This carbon loss not only decreases the SPCE and carbon selectivity for the desired products but also necessitates additional energy- and cost-intensive steps for electrolyte regeneration or CO_2_ recovery, along with other operational challenges, ultimately undermining the environmental and economic benefits of the process.

#### 6.1.1 Carbon Loss in Flow Cell: Electrolyte Carbonation

The differences in electrolyzer structure result in distinct carbon loss challenges for flow cells and MEAs. In flow cells, carbon loss primarily arises from carbonate formation and the associated costs of electrolyte regeneration back to alkaline and CO_2_. CO_2_ can react not only with cathodically generated OH^–^ but also has ready access to the bulk electrolyte. When a highly alkaline electrolyte is used, CO_2_ reacts rapidly with OH^–^, while even in mild catholytes, CO_2_ remains soluble and still participates in carbonate formation via reaction with in situ generated OH^–^. Consequently, carbon loss in flow cell configurations is significant and largely unavoidable compared to MEAs.

Although flow cells utilizing mild electrolytes generally exhibit less severe carbon loss than their alkaline counterparts, they also suffer from lower FE for C_2+_ products. This is because high-alkaline environments are well known to promote C–C coupling. Additionally, high pH conditions help minimize the full-cell voltage, making these systems appear to achieve higher eEE. Figure 16a shows that the highest pH yields the lowest full-cell voltage. The contributions shown are the thermodynamic cell potential (*E°*_cell_), and cathode, anode, and BPM overpotentials (η). Electrode potentials are referenced to the SHE scale. For simplicity, cell resistance was not included, which would add to the cell voltage. The green dotted line represents the cathode potential reported for low-temperature CO_2_ reduction at >200 mA/cm^2^.^157^ The red dotted lines provide a visual reference for cell voltages. The thermodynamic potentials versus SHE for the cathode (*E°*_cathode_) and anode (*E°*_anode_) shift positively as the pH is decreased. Furthermore, Figure 16a does not account for the increased resistance inherent to mild electrolytes such as H_2_O or KHCO_3_ due to their low conductivity, which further raises the cell voltage and decreases overall eEE.

**Figure 16:**
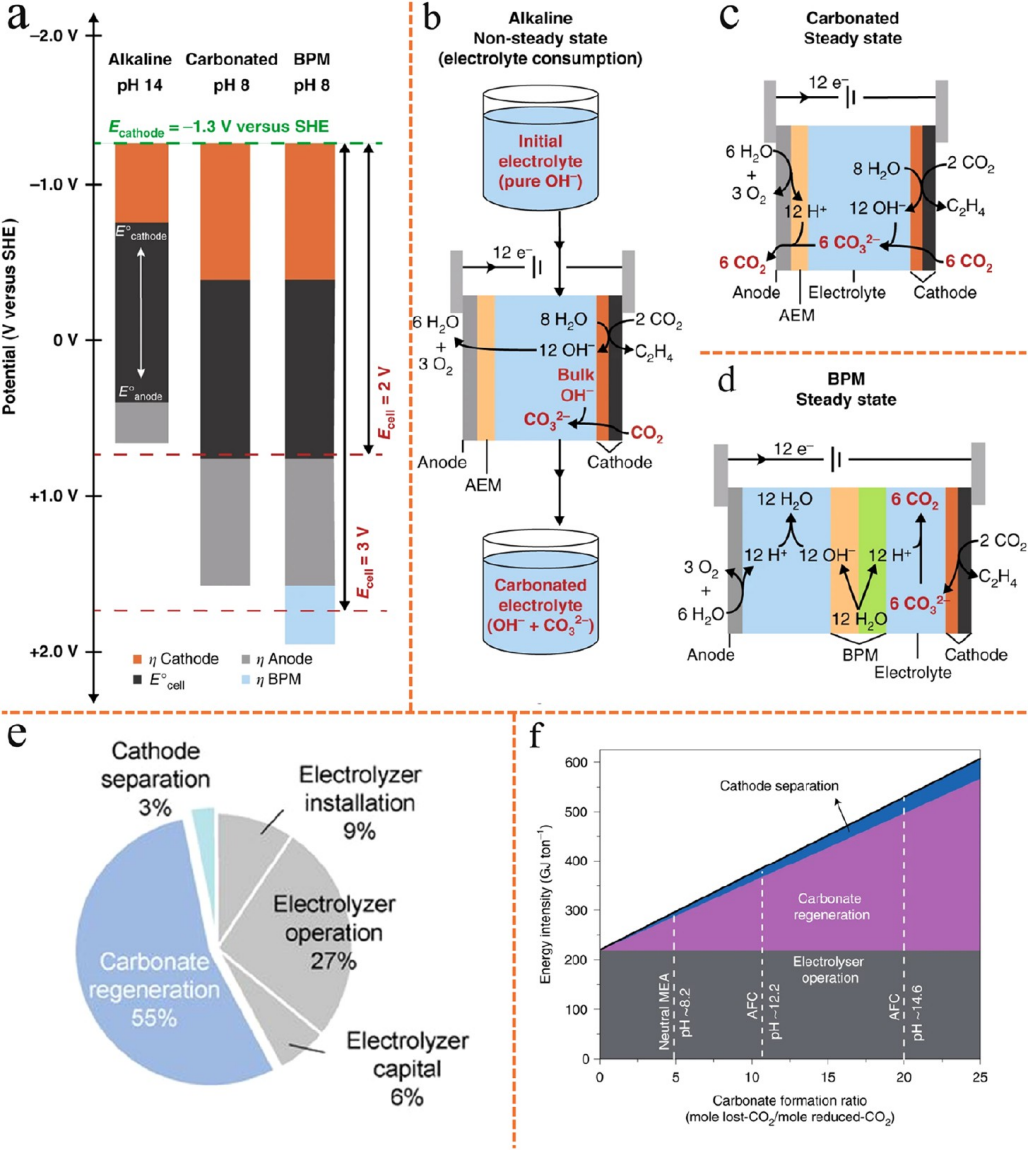
Carbonate problem and analysis of cost and energy penalties associated with carbon loss in eCO_2_RR. (a) Visualization of various contributors to the cell voltage for a conventional alkaline flow cell eCO_2_RR, carbonated conditions, or with a BPM formation. Reproduced from ref 192. Copyright 2020 Springer Nature. (b) Schematic of anion transport in an alkaline flow cell showing OH^–^ consumption by CO_2_. Reproduced from ref 192. Copyright 2020 Springer Nature. (c) Schematic of a cell at steady state after carbonation showing the carbon loss due to CO_2_ released at the anode.^192^ Reproduced from ref 192. Copyright 2020 Springer Nature. (d) Schematic of a BPM cell at steady state.^192^ Reproduced from ref 192. Copyright 2020 Springer Nature. (e) Cost breakdown of an alkaline eCO_2_RR flow cell based on TEA. Reproduced from ref 195. Copyright 2021 The American Association for the Advancement of Science. (f) Energy penalty associated with carbonate formation ratio. Reproduced from ref 100. Copyright 2022 Springer Nature.

In flow cells employing a mild catholyte and anolyte, typically using the same electrolyte on both sides, such as KHCO_3_, the absence of a highly alkaline environment does not eliminate carbonate formation. CO_2_ continues to react with in situ generated OH^–^ at the cathode, leading to continuous carbonate production. In flow cells equipped with an AEM, the resulting carbonate is transported to the anode (see Figure 16c). During OER at the anode, a significant number of protons are generated, which protonate the migrated carbonate, subsequently releasing CO_2_ together with O_2_, thereby introducing additional separation challenges. Therefore, the use of mild electrolytes in flow cells is not considered a favorable approach for minimizing carbon loss and is unlikely to be adopted in advanced eCO_2_RR systems.

For flow cells utilizing highly alkaline catholytes, challenges related to full-cell voltage and selectivity are generally considered less significant compared to systems using mild catholytes, and numerous studies have reported high FEs and half-cell energy efficiencies under such conditions. Under strongly alkaline conditions, the cathode overpotential is minimized, and the OER at the anode typically proceeds with the lowest overpotential, as illustrated in Figure 16a.^192^ These factors contribute to a significant reduction in cell voltage at high pH, creating the appearance of high eEE in these systems. However, the continuous consumption of OH^–^ in the bulk electrolyte reservoir due to carbonate formation introduces new and significant challenges. Although minimizing cell voltage is advantageous, flow cells operating under strong alkaline conditions fail to achieve a true steady state, as illustrated in Figure 16b. Hydroxide ions in the electrolyte reservoir are continuously depleted through exergonic carbonate formation upon contact with CO_2_ at the cathode. In addition to this bulk interaction, the OH^–^ generated locally at the cathode surface also reacts with CO_2_, further driving carbonate formation. However, in theory, these locally generated OH^–^ ions should migrate into the bulk catholyte and subsequently cross the AEM to compensate for OH^–^ consumption at the anode during the OER. As a result, the pH gradually decreases in both the catholyte and anolyte compartments, and the bulk KOH electrolyte is eventually converted into carbonate and bicarbonate, thereby losing its original high-alkaline characteristics. This process eventually reaches a steady state where the anolyte pH stabilizes around neutral (approximately pH 8). As a result, the cell voltage rises relative to operation under strongly alkaline conditions, since the anode thermodynamic potential shifts in the positive direction and the conductivity is much lower (Figure 16a).^193^ Maintaining a highly alkaline environment therefore necessitates continuous replenishment of KOH electrolyte.

At the research level, most studies focus on short-term FE measurements or mechanistic investigations, where this issue is often not apparent; in such cases, the effect of carbonate formation can be mitigated simply by replacing the catholyte with a fresh batch. Consequently, flow cells with strong alkaline catholytes, commonly 1 M KOH, are primarily used in research settings, and references to “flow cell eCO_2_RR” typically imply the use of highly alkaline electrolytes. However, for practical and long-term operation, the persistent formation of carbonate and the resulting energy and cost penalties present critical challenges to the viability of alkaline flow cells. In these systems, carbonate formation leads to substantial CO_2_ loss. To maintain the desired high-alkaline environment in alkaline flow cells, it becomes necessary to continuously regenerate the carbonated electrolyte back to CO_2_ and alkaline, further increasing operational complexity and cost. This issue becomes especially important when considering TEA. For example, the energy needed to convert aqueous carbonate (CO_3_^2–^) back into CO_2_ and OH^–^ exceeds 230 kJ/mol even in optimized systems using a calcination cycle.^194^ Some TEA studies have shown that 55% of the total cost in alkaline flow cells eCO_2_RR is attributable to the regeneration of CO_2_ and alkaline electrolyte from carbonate electrolyte, as illustrated in Figure 16e.^195^ Other reports indicate that electrolyte regeneration can account for 60–75% of total energy input.^95,99,100^ In contrast, as shown by the experimental results of Ozden et al.^100^ (see Figure 16f), in alkaline flow cells operating under strongly basic conditions (pH > 14), more than 90% of the CO_2_ feed is consumed by extensive carbonate formation instead of being electrochemically reduced. The associated energy demand for separation and recovery processes becomes prohibitive under these conditions. Without addressing (bi)­carbonate formation, eCO_2_RR is unlikely to be economically viable; in fact, production costs can be reduced by more than 90% if carbonate formation is effectively managed.^196^

Collectively, these findings indicate that the alkaline flow cell is not a viable or commercially profitable configuration for eCO_2_RR. While flow cells with alkaline electrolytes are valuable for catalyst development, screening, and mechanistic studies at the laboratory scale, they are not suitable for addressing carbon loss issues in eCO_2_RR. Although carbon loss is less pronounced in neutral flow cells, their low product selectivity and high ohmic losses render them unsuitable for practical applications. In alkaline flow cells, carbon loss predominantly occurs via CO_2_ conversion to carbonate, which accumulates in the catholyte. Given these limitations, flow cell reactors are not an effective solution to the carbon loss problem. In contrast, MEA configurations offer a more promising pathway for overcoming carbon loss and advancing the practical implementation of eCO_2_RR.

#### 6.1.2 Carbon Loss in MEA: CO_2_ Crossover and Salt Precipitation

In MEA systems, the elimination of catholyte significantly reduces carbon loss, as evidenced in Figure 16f, where the neutral MEA (pH = 8.2) exhibits only one-quarter of the carbon loss observed in the alkaline flow cell (pH = 14.6). This reduction in carbon loss also substantially lowers the energy penalty associated with electrolyte regeneration and CO_2_ recovery. Additionally, the zero-gap architecture of MEAs leads to reduced ohmic resistance and improved full-cell voltages, thereby enhancing eEE relative to flow cells. As a result, MEAs have become the most widely studied and dominant electrolyzer type in eCO_2_RR research. However, despite the removal of catholyte, carbon loss in MEAs remains a major challenge. In MEAs, carbon loss remains notable, though less severe than in flow cells. A significant fraction of the CO_2_ feed is converted to carbonate through reactions with OH^–^ generated locally at the cathode. Local proton consumption during eCO_2_RR induces an alkaline environment near the catalyst, where CO_2_ combines with OH^–^ to form CO_3_^2–^, with one equivalent produced for every two electrons transferred. Thermodynamic equilibrium considerations indicate that, under these strongly alkaline conditions at the catalyst–AEM interface, the reaction of hydroxide with CO_2_ predominantly forms carbonate ions rather than bicarbonate.^197–199^ Once carbonate is formed, it can migrate in two directions. First, carbonate may move backward to the backside of the GDE, leading to salt precipitation. However, carbonate ions that migrate to the backside of the GDE encounter a CO_2_-enriched and humid environment after passing through the water-humidified chamber. Under these conditions, the carbonate ions are gradually converted to bicarbonate ions, which have much lower solubility and are therefore more prone to precipitation. This process obstructs gas diffusion channels, promotes cathode flooding, drastically reduces the CO_2_ SPCE, and can cause premature device failure, a phenomenon and mechanism discussed previously and illustrated in Figure 1a–d. This type of carbon loss also contributes to the total carbon loss and presents significant challenges for long-term MEA operations.

Alternatively, carbonate anions CO_3_^2–^ formed by the carbonation can migrate toward the anode side through electromigration. In addition to the thermodynamically favored formation of carbonate over bicarbonate, experimental studies by Kim et al.^61^ have examined CO_2_ crossover in conventional eCO_2_RR electrolyzers, neutral MEA. Figure 17a, in which actual CO_2_ flow out (blue) is much less than the theoretical CO_2_ flow, which considers no CO_2_ crossover (red), confirms that significant CO_2_ loss occurs in the cathode gas stream. In fact, the data show that, at all applied currents, the amount of CO_2_ lost is approximately equal to the amount reduced to form CO, highlighting a substantial carbon loss issue. This observation aligns with the mechanistic understanding that, for each CO_2_ molecule reduced to CO, two electrons are transferred and two OH^–^ ions are generated; these OH^–^ ions subsequently react with one additional CO_2_ molecule to form one carbonate ion. If, instead, each pair of OH^–^ ions reacted with two CO_2_ molecules to form two bicarbonate ions, the amount of CO_2_ lost would be expected to be twice the amount reduced to form CO. The experimental finding of a 1:1 ratio supports the conclusion that carbonate, rather than bicarbonate, is the primary species involved in CO_2_ crossover.

**Figure 17:**
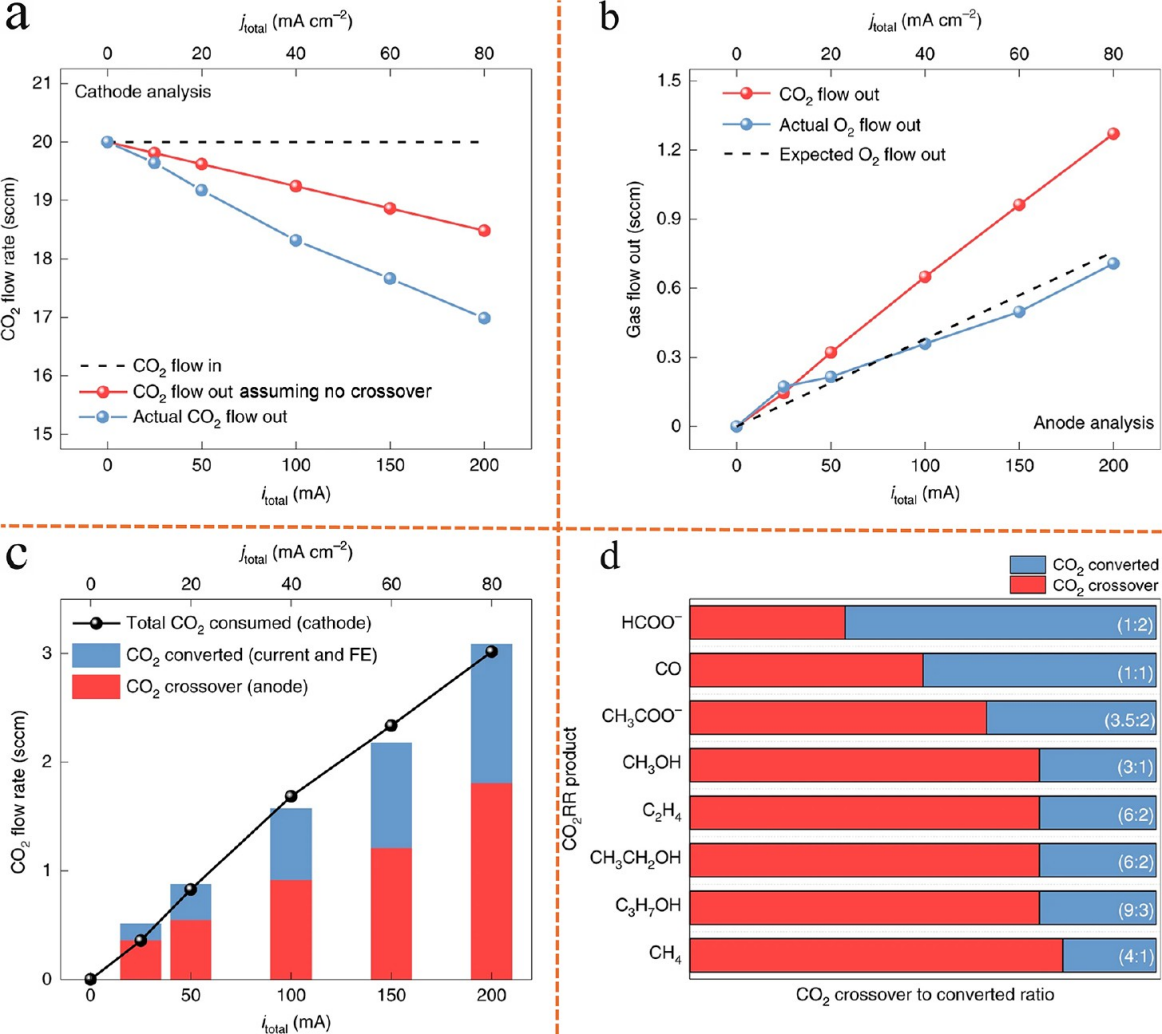
Mechanistic studies of CO_2_ crossover behavior in neutral MEA systems and cost of ethylene production in tandem electrolyzers eCO_2_RR. Reproduced from ref 61. Copyright 2022 Springer Nature. (a) CO_2_ flow analysis of the cathode side. (b) Total gas flow analysis of the anode side. (c) Total CO_2_ flow analysis. (d) Theoretical limit ratio of crossover CO_2_ to converted CO_2_ for different eCO_2_RR products.

At the anode, besides the expected O_2_ from the OER, a substantial CO_2_ flux is also observed, confirming that CO_2_ indeed permeates through the AEM into the anode compartment. Figure 17b shows that electrochemically calculated O_2_ flow out (dashed line) and measured O_2_ flow out (blue) are far exceeded by the crossover CO_2_ gas flow (red). Natural diffusion of CO_2_ across the membrane can be ruled out, as no CO_2_ was detected at the anode without applied current; therefore, the crossover must occur in the form of anionic species. Detailed analysis of the CO_2_ crossover rate, particularly at higher currents, reveals that it is approximately twice the O_2_ generation rate. Mechanistically, since the CO_2_-to-CO reaction is a two-electron process and water oxidation to O_2_ is a four-electron process, the molar O_2_ production rate is half that of CO formation. The amount of carbonate generated matches the amount of CO produced and is thus twice the amount of O_2_ formed; when these carbonate ions cross the AEM and react with protons generated locally at the anode, they produce an equivalent amount of CO_2_, again, twice the O_2_ produced. Notably, the CO_2_ flow rate at the anode outlet gas, when combined with the CO production rate, is consistent with the overall CO_2_ consumption rate determined at the cathode, corroborating this mechanism (Figure 17c). These strong correlations indicate that CO_2_ crossovers primarily occur via carbonate ions rather than bicarbonate.^59,197–200^ Collectively, these findings indicate that a substantial fraction of the CO_2_ fed into the system crosses over to the anode, where it mixes with O_2_. This crossover results in significant energy losses and increases operational costs in the eCO_2_RR process.

Following the migration of carbonate ions, their behavior in the anode compartment varies depending on the electrolyte used. When an alkaline anolyte such as 1 M KOH is employed, the anolyte gradually deteriorates as the local pH decreases due to the generation of protons via the OER. These protons first react with OH^–^ present in the bulk electrolyte, which maintains a high pH. Although migrated carbonate ions can react with locally generated protons at the anode to release CO_2_ and H_2_O, the released CO_2_ is rapidly absorbed by the excess OH^–^ in the bulk anolyte, reforming carbonate until the KOH is neutralized to K_2_CO_3_ or KHCO_3_ to some extent. While the use of KOH as an anolyte is acceptable for short-term laboratory catalyst screening or mechanistic studies, it becomes impractical for long-term or large-scale applications, as KOH will inevitably be neutralized by “crossover CO_2_”. This is further supported by the findings of Kim et al.^61^ that CO_2_ crossover is current-dependent and leads to gradual neutralization of KOH. Consequently, bicarbonate or other neutral/near-neutral electrolytes are more suitable for practical MEA operation. Notably, in MEA systems, the cathode catalyst generates a locally alkaline environment without the need for a high-alkalinity anolyte, and the zero-gap architecture minimizes ohmic losses, offsetting the lower conductivity of bicarbonate electrolytes.

Therefore, MEA systems with mild electrolytes are now generally preferred over those with alkaline electrolytes for practical applications; unless otherwise specified, subsequent analyses refer to mild MEA designs. In MEAs employing a mild anolyte such as bicarbonate, the migration of carbonate ions is followed by a local pH decrease at the anode due to proton generation from the OER. These protons react with bicarbonate, regardless of whether it originates from the cathode or the bulk anolyte to release CO_2_ alongside O_2_, resulting in substantial loss of CO_2_ feedstock. Consequently, the anode gas stream contains a mixture of O_2_ and CO_2_, necessitating considerable energy input for gas separation and CO_2_ recovery. However, the cost of CO_2_ recovery from the mild MEA anode gas stream is considerably lower than the cost associated with CO_2_ recovery and electrolyte regeneration in alkaline systems (Figure 7c). Thus, among the four possible combinations, flow cell or MEA, with either mild or alkaline electrolyte, MEA with a mild electrolyte is identified as the most favorable configuration.

Based on the principles outlined above, for every two electrons transferred in eCO_2_RR, two OH^–^ ions are generated, which can subsequently react to form one carbonate ion, except in cases where anionic products such as formate or acetate are produced. The CO_2_ crossover issue becomes more pronounced when targeting higher-value products. For instance, the synthesis of each mole of ethylene (a C_2_ product) from two moles of CO_2_ is accompanied by the crossover of six moles of CO_2_ to the anode, resulting in a crossover-to-conversion ratio of 3:1, as illustrated for various eCO_2_RR products in Figure 17d. This corresponds to a theoretical maximum carbon efficiency of only 25%. This value represents an upper limit, assuming 100% FE; in practice, competing HER and the formation of other side products further increase CO_2_ crossover and decrease carbon efficiency. This calculation also assumes that all CO_2_ feed is either reduced or converted to carbonate. It should be noted that the relationship described above is not an absolute model; other pathways are possible. For example, locally generated OH^–^ may cross the AEM without reacting with CO_2_. Such scenarios would reduce CO_2_ crossover and improve the theoretical maximum SPCE. Nevertheless, while the mild MEA has been identified as the best available option for minimizing carbon loss and associated costs, carbon loss remains a significant obstacle to achieving cost-effective CO_2_ electrolysis even in this electrolyzer type.

#### 6.1.3 Methods of Solving Carbon Loss

As discussed above, zero-gap MEAs are considered the optimized electrolyzer type for eCO_2_RR, and efforts to address carbon loss are primarily focused on solving this issue within MEAs. CO_2_ crossover, the main form of carbon loss in MEAs, predominantly occurs in mild or alkaline electrolytes, suggesting that shifting to acidic media could be a potential solution. In acidic systems, carbonate formation, and thus CO_2_ crossover can be nearly eliminated.^60,201–204^ Specifically, when protons serve as the reactant for CO_2_ reduction, OH^–^ is not produced, which prevents carbonate formation. In contrast, when water provides the protons, any carbonate generated is restricted to the diffusion layer and is subsequently reconverted to CO_2_ by proton interactions in the bulk electrolyte.

Recent studies have demonstrated high CO_2_ SPCE under acidic conditions. Sargent and co-workers demonstrated high carbon efficiency (77%) in pH 0.8 (using 1 M H_3_PO_4_ with 3 M KCl), which is much higher than the benchmark carbon utilization efficiency in neutral and alkaline solutions.^195^ However, selectivity for value-added multicarbon products remains much lower than in neutral or alkaline systems, mainly due to the more favorable HER in strong acids. To suppress the competitive HER, it is often necessary to employ a diluted acidic electrolyte combined with a high concentration of alkali metal cations,^195,205,206^ aiming to achieve a high local pH at the cathode interface during electrolysis. However, this approach can exacerbate salt precipitation within the GDE.^206–208^ Additionally, the high full-cell voltages and low C_2+_ FEs in acidic environments CEM-based electrolyzers lead to greater energy intensity for ethylene production compared to conventional AEM-based electrolyzers. The acidic environment also undermines the stability of benchmark catalysts such as copper, causing erosion and loss of active sites. Current state-of-the-art results indicate that achieving high FE, eEE, SPCE, and long-term stability simultaneously in acidic eCO_2_RR systems remains a significant challenge.^108^

An alternative strategy to mitigate carbon loss is to employ a BPM rather than an AEM or CEM. During steady state operation, carbonate ions formed in the catholyte are protonated by H^+^ generated through water dissociation at the BPM interface, thereby releasing CO_2_ from the aqueous carbonate species (Figure 16d).^209–211^ Although this configuration prevents CO_2_ crossover, such configurations generally exhibit lower eEE compared to AEM cells, as the BPM introduces additional overpotential to drive water dissociation and is associated with high internal resistance (Figure 16a).^212,213^ While CO_2_ crossover is diminished when using a BPM, it is not fully avoided.^214^ Consequently, BPM cells that begin operation with a highly alkaline anolyte will eventually equilibrate to a carbonate-based electrolyte at steady state. Furthermore, CO_2_ bubbles generated at the membrane interface can cause physical separation between the acidic and basic layers of the membrane, further impacting performance.^213,215^

An alternative strategy is the use of porous solid electrolyte (PSE) reactors, which incorporate a layer of acidic polyelectrolyte particles within the electrolyzer. This design efficiently converts permeating CO_3_^–^/HCO_3_^–^ anions to CO_2_ in the middle layer for recovery, thereby preventing their migration to the anode where the OER occurs.^61,216^ While PSE reactors can recover high-purity CO_2_, challenges remain with respect to improving the structural stability of the porous middle layer and minimizing interfacial contact resistance.^217^

The tandem electrolyzer strategies are proposed to minimize carbon loss associated with CO_2_ crossover. In this approach, the first cell focuses exclusively on the simple two-electron CO_2_-to-CO conversion, producing an output gas stream rich in CO and unreacted CO_2_. After removing the excess CO_2_, the CO-rich stream is fed into the second cell, where a highly alkaline electrolyte can be used to promote C–C coupling and achieve lower ohmic loss and reduced full-cell voltage, without concern for carbonate formation and following CO_2_ crossover. Compared to the single-cell eCO_2_RR for C_2+_ product synthesis, the theoretical SPCE limit in the tandem process can reach 50%, versus only less than 25% for the single-cell approach. This is because, in the tandem configuration, the first cell carries out the conversion of CO_2_ to CO via a two-electron transfer process, during which two OH^–^ ions are generated for every CO molecule produced. These OH^–^ ions react with a single CO_2_ molecule to form one carbonate, and no further carbon loss related to carbonate formation occurs in the second cell. As a result, the theoretical SPCE limit for the tandem system is 50%. By contrast, in the direct production of C_2_H_4_ from CO_2_ in a single cell, two CO_2_ molecules are converted to one C_2_H_4_ through a 12-electron transfer process, generating 12 OH^–^ ions. These subsequently react with six CO_2_ molecules to form six carbonates, resulting in a SPCE limit of only 25%. This comparison underscores the significant advantage of the tandem strategy in reducing carbon loss associated with carbonate formation.

Furthermore, the tandem electrolyzers system offers versatile opportunities for stepwise optimization, enabling the first cell to achieve fully carbon-loss-free eCO_2_RR. By dividing the complex one-step eCO_2_RR to C_2+_ products into two simpler steps, the tandem design allows the first cell to exclusively focus on CO production, which can be accomplished with no carbon loss. This is enabled using SOECs, which circumvent carbonate formation and achieve eEEs exceeding 80% with a CO_2_ SPCE of approximately 45%.^99^ Moreover, these systems have already been scaled to industrial levels, with large SOEC stacks featuring active areas up to 8250 cm^2^ demonstrating stable operation at current densities of 450 mA/cm^2^ for more than 2000 h.^218,219^ These results indicate that current SOEC technology can economically produce CO with high productivity and eEE. However, a major obstacle for SOECs is the simultaneous occurrence of CO formation (2CO_2_ (g) → 2CO (g) + O_2_ (g)) and undesired carbon deposition (CO_2_ (g) → C (s) + O_2_ (g)). This side reaction undermines operational stability and restricts the CO_2_ SPCE, keeping it under 50%.^220^ As shown in Figure 5e,f, the tandem electrolyzers configuration for eCO_2_RR demonstrates significant potential for profitable and commercial-scale ethylene production. This is attributed to the absence of carbonate formation in the SOEC during the initial CO_2_-to-CO step and the high efficiency attainable in the subsequent eCORR step.

In addition to using SOECs for carbon-loss-free CO production, there are promising strategies to improve MEA systems and mitigate CO_2_ crossover-induced carbon loss. Operating at near-neutral pH increases solution resistance and the overpotential required for the OER at the anode, resulting in high cell voltages and low eEE. At the anode, the OER requires high thermodynamic potential and proceeds with slow kinetics, while the resulting O_2_ byproduct has relatively low economic value.^221–225^ Furthermore, OER is always accompanied by proton production, which exacerbates carbon loss and further increases the energy intensity, as discussed above. Thus, the concept of “paired electrolysis” has emerged as a prominent research topic.^224,226–229^ In paired electrolysis, eCO_2_RR can be integrated with anodic reactions that are thermodynamically and/or kinetically more favorable than OER, thereby avoiding producing O_2_ or gas mix with crossover CO_2_. Various organic electrooxidation processes, such as those involving aldehydes, glycerol, ethanol, isopropanol, and 1,2-propanediol, have been explored as potential anodic reactions.^224,229–232^ These anodic reactions enhance overall eEE and simultaneously allow the coproduction of value-added chemicals.^233^

Xie et al.^234^ demonstrated that using a liquid–liquid anodic reaction allows efficient capture of crossover CO_2_ through simple gas–liquid separation, eliminating the requirement for extra energy consumption. The recovered anode gas was directly recycled into the cathode stream together with fresh CO_2_ feed. The system achieved a full-cell voltage as low as 1.9 V and reached a total carbon efficiency of 48%. This is achieved by simply replacing the OER at the anode with organic oxidation reactions (OORs), such as the GOR, in which glucose is oxidized to liquid products like gluconate, glucuronate, and glucarate. As a result, no gaseous oxidation product such as O_2_ is generated to mix with the CO_2_ stream. Also GOR can proceed more favorably than OER under mild operating conditions, achieving reaction rates that are more relevant for industrial applications (1 M KHCO_3_ in this study).^235^ The mechanism by which the CO_2_RR-OOR MEA electrolyzer facilitates efficient CO_2_ recovery from the anode tail gas mixture is illustrated in Figure 18a. Furthermore, GOR significantly reduced the full-cell voltage, with a thermodynamic potential of just 0.05 V,^224^ much lower than that of OER for 1.23 V. The principle of eCO_2_RR-OOR paired electrolysis, integrating reduced energy input with efficient carbon utilization, is shown in Figure 18b. While the work by Xie et al. focused on a single-cell CO_2_-to-C_2+_ process paired with GOR, this approach of replacing OER with GOR can also be implemented in the first step of a tandem electrolyzers eCO_2_RR system for CO production. In doing so, the carbon loss problem at this stage can be nearly eliminated, while retaining the original advantages of GOR pairing, namely, lower overpotential losses, reduced energy input, and the coproduction of value-added products.

**Figure 18:**
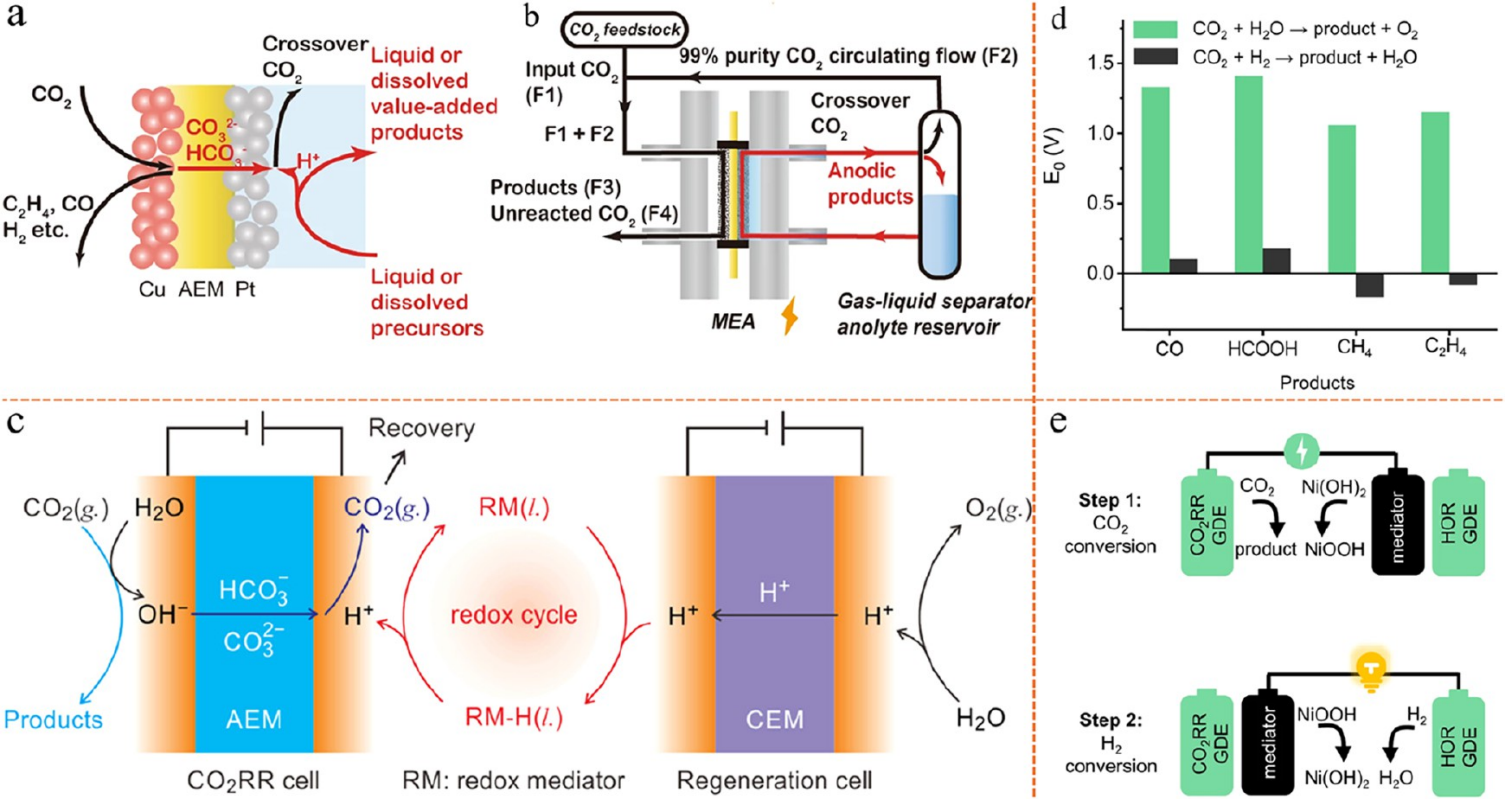
Mechanistic studies of CO_2_ crossover behavior in neutral MEA systems and cost of ethylene production in tandem electrolyzers eCO_2_RR. (a) Mass balance of the electrochemical process in the CO_2_RR-OOR MEA electrolyzer. Reproduced from ref 234. Copyright 2022 Springer Nature. (b) Operating principle of the CO_2_RR-OOR electrolysis system combines low-energy input and high carbon utilization in CO_2_-to-C_2+_ conversion. Reproduced from ref 234. Copyright 2022 Springer Nature. (c) Working principle of redox mediator (RM)-coupled electrolysis mode. Reproduced from ref 189. Copyright 2025 John Wiley and Sons. (d) Comparison of Nernst potentials (*E*_0_) between conventional and H_2_-integrated eCO_2_RR. Reproduced from ref 237. Copyright 2024 Springer Nature. (e) Detailed working principles of the H_2_-integrated eCO_2_RR cell. Reproduced from ref 237. Copyright 2024 Springer Nature.

However, when comparing the use of GOR to replace OER in a single-cell eCO_2_RR process with the tandem electrolyzer strategies, the latter offers clear advantages and superior performance. In the single-cell configuration, CO_2_-to-C_2+_ conversion paired with GOR at the anode does confer benefits, but similar or greater advantages can be realized in the tandem approach, where GOR can be employed in both cells’ anode reactions. As previously discussed, single-cell MEA eCO_2_RR operating in mild electrolyte (such as KHCO_3_) is currently the preferred configuration when the input is CO_2_, since alkaline systems incur a significant energy penalty for regenerating (bi)­carbonate back to CO_2_ and alkaline. Additionally, the use of low conductivity of KHCO_3_ across the entire CO_2_-to-C_2+_ process increases the full-cell voltage and lowers the selectivity and EE, especially compared to the tandem design, where the first cell (MEA, near-neutral electrolyte) performs CO_2_-to-CO conversion and the second cell (highly alkaline electrolyte, e.g., 6 M KOH) carries out CO-to-C_2+_ conversion with much higher efficiency. Importantly, coupling eCORR with OOR, specifically GOR, has been demonstrated in an MEA electrolyzer.^99^ In the second step (eCORR), the use of a KOH-glucose electrolyte lowers the cell voltage by nearly 1 V at industrially relevant current densities, resulting in about a 35% reduction in energy demand compared with an MEA cell employing OER at the anode under similar conditions.

While OORs can reduce cell voltage and improve laboratory-scale efficiency, their practical, large-scale techno-economic feasibility is highly limited by feedstock purity, cost, inconsistent supply, product separation complexity, and limited market demand for byproducts. The choice of anodic reaction must carefully consider these economic and logistical factors, not just theoretical energy savings.^236^ In addition, there are prominent potential limitations to the single-cell paired strategy, particularly regarding market size mismatches between cathodic and anodic products.^237^ For instance, the volume of industrial CO_2_ emissions that could be captured and utilized is on the gigatonne scale per year, while the annual market demand for most possible anodic coproducts from paired electrolysis is typically on the million-tonne scale or less. In practice, for the production of one mole of C_2_H_4_ in a single-cell configuration, 12 electrons must be transferred from glucose for every two CO_2_ molecules reduced. In contrast, in the tandem system, only four electrons from glucose are required for the same amount of CO_2_ processed, a two-thirds reduction, representing a significant improvement in efficiency. Furthermore, the second step in the tandem configuration can be paired with a wider range of anodic reactions without concerns about the physical state of the anodic products because no CO_2_ crossover loss in the anode tail gas in eCORR, further illustrating the advantages of the tandem electrolyzers design.

Other strategies have also been explored to address CO_2_ crossover-related carbon loss, similar in concept to pairing CO_2_ reduction at the cathode with alternative anodic reactions such as GOR. These approaches involve using anodic reactions that do not generate gaseous oxidation products. Ideally, the oxidation product could also serve as a substrate for a subsequent reduction process, creating a cyclical redox system within the eCO_2_RR framework. For example, Yu et al.^189^ proposed a redox mediator (RM)-coupled electrolysis strategy to mitigate transmembrane CO_2_ loss in alkaline CO_2_ electrolysis. By incorporating a highly reversible redox couple, this method spatially separates cathodic CO_2_ reduction and anodic OER into two distinct electrolyzers, thereby enabling recovery and reuse of transmembrane CO_2_. The working principle of redox mediator (RM)-coupled electrolysis mode is illustrated in Figure 18c. However, the overall energy intensity and cost implications of employing a redox-mediated two-electrolyzer system have yet to be fully evaluated.

Jiang et al.^237^ coupled CO_2_ electrolysis with the hydrogen oxidation reaction (HOR) in a single electrolyzer, where a mediator was applied to eliminate anodic carbon loss and to protect the hydrogen oxidation catalyst from deactivation caused by crossover reaction products. Compared with conventional eCO_2_RR, the Nernst potentials of H_2_-integrated systems under standard conditions are significantly reduced for four representative conversions (Figure 18d). The reduction potentials for CH_4_ and C_2_H_4_ formation drop to −0.17 and −0.08 V, respectively, suggesting that these pathways could even allow for electricity generation. To realize efficient H_2_-assisted eCO_2_RR, the single-cell system employs a Ni­(OH)_2_/NiOOH mediator positioned between a eCO_2_RR GDE and a HOR GDE, thereby avoiding the limitations of OER. In Step 1, CO_2_ is reduced to CO or formate at the eCO_2_RR GDE, while Ni­(OH)_2_ is oxidized to NiOOH. Upon depletion of Ni­(OH)_2_, the Ni electrode subsequently operates with the HOR GDE in Step 2, which functions analogously to a Ni–H_2_ battery, generating electricity that partially offsets the power consumed in Step 1 (Figure 18e). Notably, even after accounting for the energy required to generate hydrogen, the H_2_-integrated CO_2_RR process was found to reduce total energy consumption by 42%, owing to the transfer of the OER to a separate water electrolyzer operating under more favorable conditions. However, this system operates through temporally and spatially separated Step 1 and Step 2 in an alternating sequence, which raises concerns regarding process continuity and scalability.

### 6.2 Product Separation

Product separation is a critical, yet often underestimated, challenge in the commercialization of eCO_2_RR technologies, particularly as systems move from proof-of-concept toward integrated tandem designs. Downstream purification not only dictates the final product quality but also has a profound impact on overall energy consumption and process economics.^130^

In current single-cell eCO_2_RR systems, the gas stream exiting the electrolyzer is a complex mixture of target products (e.g., ethylene), unreacted CO_2_, and various byproducts such as CH_4_, H_2_, and CO. Achieving industry-grade purity (e.g., polymer-grade C_2_H_4_, >99.9 vol %) typically relies on a series of mature, energy-intensive separation technologies. The article highlights^130^ that CO_2_ removal is the most energy-intensive step in downstream processing. For example, the energy required for CO_2_ recapture from the anode tail gas can be up to 1.6 times higher than the electricity input for the entire electrolysis step itself. As such, any unreacted CO_2_ or CO_2_ crossover from cathode to anode not only lowers overall carbon efficiency but also imposes a substantial energy and cost penalty on the separation process.

These separation challenges are further magnified in tandem electrolyzers systems, where the product stream between the first and second cell, or intermediate purification steps, may include a mixture of CO, CO_2_, H_2_, and other reaction intermediates. Each step’s output must be carefully managed to avoid the buildup of undesired byproducts that could compromise the selectivity or performance of downstream reactors. In addition, the design of tandem systems often incorporates intermediate purification units, such as NaOH or amine absorbers, to remove unreacted CO_2_ or other species prior to the subsequent electroreduction step, adding further energy and operational complexity.

Sensitivity analyses indicate that reducing CO_2_ crossover, increasing product selectivity (FE), and boosting SPCE are all pivotal for lowering separation energy demands. Improvements in these parameters can collectively decrease the separation heating load by more than 10-fold, from over 500 GJ/tonne product to as low as 22 GJ/tonne for C_2_H_4_ under optimized conditions.^130^ Tandem systems offer a unique advantage by allowing versatile integration and optimization of different tandem devices and techniques. For example, an innovative indirect reduction strategy for CO_2_-to-ethylene conversion utilizes 2-BrEtOH as an intermediate.^84^ 2-BrEtOH is first generated from CO_2_ electroreduction and then selectively reduced to ethylene, effectively bypassing energy-intensive product separation steps. More broadly, the tandem electrolyzer strategies enhance FE and reduce the formation of undesired byproducts compared to direct CO_2_ electroreduction, as each step can be independently optimized. This reduction in byproduct complexity directly translates to lower separation costs and improved overall process efficiency when compared to traditional single-cell eCO_2_RR systems.

In summary, efficient product separation remains critical, not only for meeting industrial specifications but also as a decisive factor influencing the energy and carbon footprint of eCO_2_RR processes. Overcoming this challenge will require continued innovation in both electrolyzer and tandem design to minimize downstream separation needs, as well as the development of separation technologies specifically tailored to the unique product streams generated by tandem electrolyzers eCO_2_RR systems.

### 6.3 Scale-Up

Transitioning eCO_2_RR from laboratory-scale demonstrations to industrially relevant scales is critical to achieving its commercialization potential. Tandem electrolyzers eCO_2_RR systems, which decouple the reaction into two sequential steps, offer a particularly attractive pathway for scale-up due to their modularity and operational flexibility. Unlike single-cell systems that must simultaneously optimize multiple competing reactions within one environment, tandem architecture allows independent optimization of each stage, facilitating targeted performance improvements and the use of standardized, mass producible components. This modular design also simplifies load balancing, enhances system control, and is more compatible with fluctuating renewable energy inputs, key advantages for practical deployment. Although tandem electrolyzers eCO_2_RR has demonstrated significant promise at the laboratory scale, its development remains largely restricted to watt-scale studies. For this technology to move toward real-world deployment, research must shift from isolated performance assessments of individual electrolyzers to the integrated operation of full tandem systems. Critically, advancing tandem electrolyzers eCO_2_RR to the kilowatt scale is a necessary step toward commercialization. This scale-up is not a simple matter of enlarging system components but rather involves addressing a host of new technical challenges, including effective thermal management, uniform reactant distribution over extended electrode surfaces, and long-term material stability under continuous operation.

Crandall et al.^50^ illustrates significant progress in this direction, particularly by scaling tandem CO_2_ electrolysis systems up to the kilowatt level. In their study, a tandem system composed of a 500 cm^2^ CO_2_ electrolyzer operating at 0.40 kW and a 1000 cm^2^ CO electrolyzer at 0.71 kW was successfully developed and demonstrated (Figure 19a,b). When scaling up to the kilowatt level, a stable acetate FE of approximately 30–35% was maintained throughout the experimental duration (Figure 19c). By the end of a 120 h test, a total of 98 L of ∼1.2 M acetate solution, with a molar purity exceeding 96%, was produced. However, within the initial 10 h of operation, ethylene selectivity rapidly declined to less than 2%. Postelectrolysis XPS characterization revealed a small amount of Fe on the spent cathode surface, which may have contributed to the decreased ethylene selectivity and increased hydrogen evolution. This phenomenon was also observed in small-scale experiments but did not lead to such pronounced effects. The significant drop in acetate FE (from approximately 50% in smaller-scale tests down to about 30%) and the near-complete disappearance of ethylene highlight that scaling up involves more than simply increasing the electrode area or stacking multiple cells.

**Figure 19:**
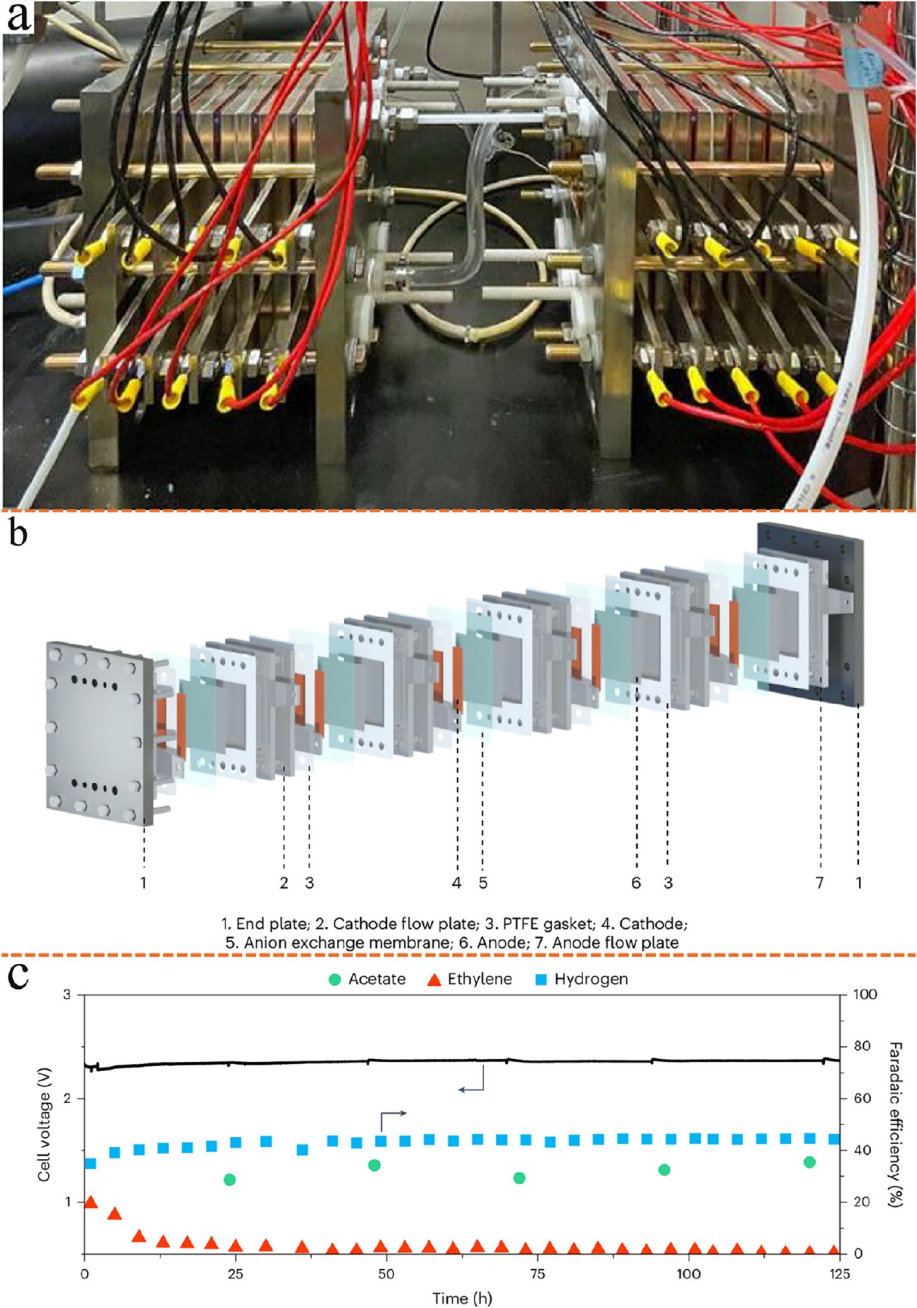
Kilowatt-scale tandem CO_2_ electrolysis for enhanced acetate production. Reproduced from ref 50. Copyright 2024 Springer Nature. (a) Photograph of kW-scale electrolyzer stacks (a total electrode area of 1000 cm^2^). (b) Schematic of a five-cell, 500 cm^2^ CO_2_/CO electrolyzer stack with polytetrafluoroethylene (PTFE) gaskets (cooling channels not pictured). (c) CO electrolysis stability for 1000 cm^2^ stack.

Such quantitative changes lead to qualitative shifts in reaction performance.^50^ Beyond the catalyst stability issues (Cu cathode and NiFeO_*x*_ anode) identified by the authors, the substantial increase in reactor size from 5 to 100 cm^2^ likely introduced pronounced gradients in reactant and product distribution across the MEA. Differences in the microenvironment between inlet and outlet regions result in averaging effects on performance metrics. Ethylene formation, requiring a higher number of charge transfers and enough intermediates for C–C coupling, might be particularly sensitive to local CO availability, necessitating further investigation to fully understand and mitigate these scale-related challenges. The pH and electrolyte composition across the flow field of a large-scale electrolyzer are highly nonuniform, resulting in a vicious cycle from the inlet to the outlet. As acetate/acetic acid accumulates and the catalyst surface pH likely drops even lower along the length of a much larger cell (e.g., 100 versus 5 cm^2^), the pH tends to drop progressively across the flow field. This further promotes Fe leaching from the NiFeO_*x*_ catalyst, which is only stable under alkaline conditions. Such large-scale effects are significantly more pronounced than in small-scale cells and may account for the sharply decreased ethylene selectivity and increased hydrogen evolution observed during scale-up. Additionally, the electrolyzer exhibited resilience against typical industrial impurities, maintaining high acetate selectivity and system efficiency, marking an important advancement toward commercially viable electrochemical acetate synthesis.

Notably, the transition from initial watt-scale setups using 5 cm^2^ electrodes to kilowatt-scale systems, comprising two five-cell stacks with a total electrode area of 1000 cm^2^ introduced several engineering challenges. Their research highlights critical factors, such as flow field design, uniform current distribution, thermal management, and system robustness that must be addressed to achieve stable and efficient operation at industrially relevant scales.^50^

Flow field channel pattern plays a critical role in determining reactant distribution and managing internal pressure gradients in large-area electrolysis systems. To address this, the impact of flow field patterning on pressure drop at various flow rates was investigated. The study revealed that a triple serpentine flow pattern induces significantly less pressure drop than a single-serpentine design. This finding is especially relevant for larger-scale electrolyzers, where longer flow channel lengths can amplify pressure differentials. Therefore, selecting an appropriate flow configuration that minimizes pressure drop is essential for ensuring uniform reactant delivery and maintaining high overall electrolysis efficiency.

Uniform current distribution across large electrode surfaces is a critical requirement when scaling tandem electrolysis systems from laboratory to industrial scales. To ensure consistent current distribution, flow plates were meticulously fabricated for maximum flatness, and silicon gaskets were incorporated between individual cells to minimize variations caused by mechanical compression. These measures were crucial for maintaining electrochemical stability and performance in large-scale operations.

Thermal management becomes increasingly critical as electrode area scales up, due to elevated resistive heating. When the single cell electrode area was increased from 5 to 100 cm^2^, a 45% rise in temperature was observed under identical current densities. This thermal buildup poses a significant challenge, especially considering the limited thermal stability of AEM. To address this, cooling channels were integrated into the cell design, enabling effective temperature control. Without active cooling, cell temperatures exceeded 55 °C at 300 mA/cm^2^, but with the cooling system activated, the temperature was rapidly reduced to approximately 35 °C within 15 min. These findings underscore the importance of thermal regulation in maintaining system stability and membrane integrity during high-current operations.

### 6.4 Prospects

Tandem electrolyzer strategies have emerged as transformative strategies in eCO_2_RR, offering a systematic pathway to overcome the intrinsic limitations of single-cell systems. ECO_2_RR has evolved from single-cell systems, typically employing copper-based catalysts for direct CO_2_-to-product conversion, to more sophisticated tandem catalyst and reactor configurations. With mature CO_2_-to-CO technology and rapid advances in eCORR, the next trend is to integrate these approaches, enabling the efficient commercialization of multicarbon products. As the representative of the trend, tandem electrolyzer strategies are attracting increasing attention, as evidenced by a dramatic rise in publications over the past decade. Building upon conventional single-cell eCO_2_RR, it offers unique advantages through the coupling of sequential electrolyzers. By breaking down a complex single-step reaction into multiple, simpler steps, this approach allows each stage to be individually optimized, resulting in synergistic improvements in overall performance.

Grounded in the developments of first-class two-electron reduction products, such as CO and formate (HCOO^–^), which have already reached market production, the tandem methodology is poised to drive the field toward the next stage of sustainable electrocatalysis, enabling higher-order reduction products such as ethylene and other multicarbon compounds. Through careful tandem design, persistent challenges such as carbonate formation/carbon loss, which are unavoidable in single-cell eCO_2_RR with alkaline media, will no longer stop the transition of eCO_2_RR from laboratory research to industrial practice. This ultimately lowers energy input and cost for the production of multicarbon products. Importantly, the overall energy intensity of tandem processes for CO_2_ conversion is expected to fall below that of conventional fossil-fuel-based production, with costs decreasing further as renewable energy becomes more widely available and electricity prices continue to drop. TEA has already demonstrated the potential profitability and negative carbon emissions associated with renewable energy-powered production of multicarbon fuels and chemicals from CO_2_ using tandem electrolyzers eCO_2_RR strategy. Taken together, tandem electrolyzer strategies represent an environmentally friendly and economically viable path for CO_2_ utilization, bringing the vision of artificial photosynthesis ever closer to realization for human benefit.

However, limitations remain, particularly in terms of catalyst performance and the scarcity of scale-up demonstrations, which cast doubt on whether the trade-offs of tandem configurations truly outweigh those of single-cell strategies. Advancement in this field will require both technical and engineering efforts, along with thoughtful experimental design for integrated tandem systems. At the same time, the introduction of additional electrolyzers unlocks new opportunities and innovations for CO_2_ electrolysis, opening pathways to a new era of fully electrified synthesis routes, offering a promising alternative to conventional, energy-intensive, and fossil-fuel-dependent production processes across the chemical industry. However, this added complexity also means that, unlike single-cell eCO_2_RR, tandem electrolyzer approaches urgently need standardized metrics and benchmarks to support further growth and acceptance across academia and industry. The next sections expand on these points, offering perspectives on technical, engineering, system, extended product range, and standardization as critical pillars for the advancement of tandem electrolyzer strategies.

From a technical perspective, the critical challenges facing tandem electrolyzers eCO_2_RR largely originate from those encountered in single-electrolyzer systems. Catalyst stability, selectivity, and activity for both steps continue to limit the widespread deployment of tandem systems. Although tandem design reduces some of the constraints for catalyst development, achieving the requirements for commercialization, including multiyear stability, high activity at the A/cm^2^ scale, and near-unity selectivity, remains a significant hurdle. This is particularly pronounced for the second cell (CO-to-C_2+_ conversion), where multielectron transfer per carbon atom and the need for precise proton-coupling/carbon coupling render it intrinsically difficult to simultaneously attain high selectivity, activity, and stability. The development of advanced catalysts tailored for tandem applications is therefore urgently needed.

From an engineering standpoint, there are currently no reports of large-scale (kilowatt-scale) tandem electrolyzer stack operation. This challenge extends beyond simply stacking more cells or increasing electrode area; quantitative scaling can lead to qualitative changes in system behavior. For each individual step, maintaining a uniform and stable reaction environment across the entire electrolyzer and entire operation period is essential. For tandem integration, efficient recycling of CO_2_ from the cathode gas stream, immediate removal of CO_2_ to enable near-pure CO feed to the second cell, and recovery of CO_2_ from the anode tail gas all require real-time precise process control and matching of reaction rates between the two steps. Additional engineering challenges include salt precipitation (salting out) in the first cell, which can disrupt operation, and complex water management in MEAs, especially given the demands of the zero-gap architecture.

From a system perspective, the overall balance and trade-offs associated with tandem electrolyzer eCO_2_RR strategies remain to be fully elucidated. While tandem electrolyzers offer distinct advantages in addressing carbon loss, they also introduce additional capital investment, increased process complexity, and extra separation steps. As single-cell strategies continue to advance, such as with tandem catalyst architectures and acidic eCO_2_RR, the cost-benefit analysis between single-cell and tandem designs becomes increasingly nuanced. Within tandem electrolyzer systems themselves, the interplay among performance metrics, total cost, energy input, and carbon emissions is complex and not yet fully understood. Key factors, including the matching of reaction rates between the two steps, removal of intermediate CO_2_, recovery of CO_2_ from the anode, and the separation of final products from complex gas mixtures, are all interconnected and can significantly affect system efficiency and economics. To date, no single study has demonstrated a comprehensive, cradle-to-gate experimental assessment of tandem systems; most analyses rely on assumptions and calculations rather than full process demonstration. Future research should aim to provide integrated experimental and TEA to guide the rational design and optimization of tandem electrolyzer strategies.

Beyond conventional targets such as ethylene and ethanol, the future of tandem electrolyzer eCO_2_RR systems is exceptionally promising for the synthesis of a broader spectrum of chemicals. By combining multiple electrolyzers in sequence, tandem approaches can facilitate the production of compounds that are challenging or even unattainable in single-cell systems. For instance, this strategy enables access to value-added products like ethylene carbonate and succinic acid, and potentially many more, through versatile combinations of reaction types. Moreover, the modularity and flexibility inherent to tandem designs create opportunities to integrate emerging fields such as electrochemical organic synthesis, thereby expanding the range of accessible products. Freed from the limitations of a single cell, tandem systems can be specifically engineered to meet advanced industrial requirements, such as the direct production of polymer-grade ethylene. As research continues to advance, the innovative coupling of diverse electrolyzer configurations and reaction environments is poised to unlock sustainable, electrochemically driven manufacturing routes for both commodity and specialty chemicals derived from CO_2_.

As tandem electrolyzer systems usher in a new era for CO_2_ utilization, the need for rigorous standardization and benchmarking becomes increasingly critical. Owing to the complexity inherent in integrating multiple electrolyzers, performance comparisons and reported metrics must be more comprehensive and detailed than those used for single-cell systems. It is essential that benchmarking extends beyond conventional figures of merit to include metrics such as overall process cost, energy intensity, and carbon emissions, encompassing the entire system from input to final product. Given the practical and integrated nature of tandem designs, experimental studies and analyses should strive to capture all relevant process steps, from reactant handling to product separation and recycling. The development of well-considered standards and benchmarks is urgently needed to ensure that progress in this field is both transparent and comparable, thereby facilitating broader acceptance and adoption within both academic and industrial communities.
